# A Genome-Wide Association Study of Age-Related Hearing Impairment in Middle- and Old-Aged Chinese Twins

**DOI:** 10.1155/2021/3629624

**Published:** 2021-07-17

**Authors:** Haiping Duan, Wanxue Song, Weijing Wang, Hainan Cao, Bingling Wang, Yan Liu, Chunsheng Xu, Yili Wu, Zengchang Pang, Dongfeng Zhang

**Affiliations:** ^1^Department of Epidemiology and Health Statistics, Public Health College, Qingdao University, No. 38 Dengzhou Road, Shibei District, Qingdao, 266021 Shandong Province, China; ^2^Qingdao Municipal Center for Disease Control and Prevention, No. 175 Shandong Road, Shibei District, Qingdao, 266033 Shandong Province, China; ^3^Qingdao Institute of Preventive Medicine, No. 175 Shandong Road, Shibei District, Qingdao, 266033 Shandong Province, China; ^4^Department of Otorhinolaryngology, Qingdao Municipal Hospital, Qingdao, 266011 Shandong Province, China

## Abstract

**Background:**

Age-related hearing impairment (ARHI) is considered an unpreventable disorder. We aimed to detect specific genetic variants that are potentially related to ARHI via genome-wide association study (GWAS).

**Methods:**

A sample of 131 dizygotic twins was genotyped for single-nucleotide polymorphism- (SNP-) based GWAS. Gene-based test was performed using VEGAS2. Pathway enrichment analysis was conducted by PASCAL.

**Results:**

The twins are with a median age of 49 years, of which 128 were females and 134 were males. rs6633657 was the only SNP that reached the genome-wide significance level for better ear hearing level (BEHL) at 2.0 kHz (*P* = 1.19 × 10^−8^). Totally, 9, 10, 42, 7, 17, and 5 SNPs were suggestive evidence level for (*P* < 1 × 10^−5^) BEHLs at 0.5, 1.0, 2.0, 4.0, and 8.0 kHz and pure tone average (PTA), respectively. Several promising genetic regions in chromosomes (near the C20orf196, AQPEP, UBQLN3, OR51B5, OR51I2, OR52D1, GLTP, GIT2, and PARK2) nominally associated with ARHI were identified. Gene-based analysis revealed 165, 173, 77, 178, 170, and 145 genes nominally associated with BEHLs at 0.5, 1.0, 2.0, 4.0, and 8.0 kHz and PTA, respectively (*P* < 0.05). For BEHLs at 0.5, 1.0, and 2.0 kHz, the main enriched pathways were phosphatidylinositol signaling system, regulation of ornithine decarboxylase, eukaryotic translation initiation factor (EIF) pathway, amine compound solute carrier (SLC) transporters, synthesis of phosphoinositides (PIPS) at the plasma membrane, and phosphatidylinositols (PI) metabolism.

**Conclusions:**

The genetic variations reported herein are significantly involved in functional genes and regulatory domains that mediate ARHI pathogenesis. These findings provide clues for the further unraveling of the molecular physiology of hearing functions and identifying novel diagnostic biomarkers and therapeutic targets of ARHI.

## 1. Introduction

Hearing impairment is the most prevalent sensory deficit, affecting over 50% of middle-aged people and the elderly in China [1, 2]. Age-related hearing impairment (ARHI) or presbycusis is the most common type of sensorineural hearing loss caused by the natural aging of the auditory system. It is considered an unpreventable and incurable disorder [3]. The typical characteristics of ARHI are gradual progression in later life and bilaterally symmetrical sensorineural hearing impairment, which starts at high frequencies in the early stages and then extends to medium and low frequencies over time. However, early-stage ARHI is often underrecognized. ARHI is a complex, multifactorial disease that is attributable to confounding genetic and environmental factors [4, 5]. The genetic predisposition to hearing impairment variations approximately have accounted for 25%-75% [5–11]. Genome-wide association study (GWAS) has had an enormous impact on our understanding of the molecular physiology of hearing impairment and has allowed the identification of several genetic loci located at or near the *GRM7* [12, 13], *DCLK1*, *PTPRD*, *GRM8*, *CMIP*, *ISG20*, *ACAN*, and *TRIOBP* genes [14].

Only a small part of the genetic variants is explained by known genetic variation and many potential genes to be further discovered. As modern medicine cannot cure ARHI, the active prevention of it is particularly important. The twin study design relies on study twins raising in the same family environment. Simultaneously, on average, dizygotic (DZ) twins share 50% of the same genes, which can not only be regarded as ordinary sibling pairs but also have perfectly matched ages, prenatal intrauterine environment, and very similar life environment. Therefore, association analysis using DZ twins is more conducive for interpretation of the results. However, the molecular physiology of hearing impairment in middle- and old-aged Chinese population have not been investigated via GWAS yet. This undertaking is important because this population differs from other ethnic populations worldwide in terms of genetic constitutions and lifestyles.

Investigations into genetically related individuals, such as twins, will enhance genetic association studies, and the use of twin-based designs can efficiently identify both common and rare genetic variants underlying complex traits or diseases [15]. A previous study explored the magnitude of genetic impact on better ear hearing levels (BEHLs) and variations in pure tone average (PTA) via twin modelling analyses. Results indicated that heritability estimates range from 47.08% to 54.20% for BEHLs within 2.0-12.5 kHz [16]. Owing to the lack of studies on ARHI among middle- and old-aged Chinese twins via GWAS, we further conducted a GWAS to detect the specific genetic variants potentially associated with ARHI. We expect to be able to identify genetic mutations associated with ARHI and elucidate biological processes.

## 2. Materials and Methods

Twin Samples CollectionSamples of twins were collected from the latest genetic epidemiology survey (2012-2013) on previously described aging phenotypes [16–18]. In brief, information was collected via questionnaires and health examination, including anthropometric and laboratory measurements by well-trained clinicians. Participants were excluded if they were unconscious; unable or unwilling to participate; suffering from heart failure, kidney failure, cancer, or severe mental disorders; and currently pregnant or breast feeding; incomplete cotwin pairs were also dropped. Zygosity was determined using 16 multiple short-tandem sequence repeat DNA markers [19–21]. Finally, the samples consisted of 131 complete DZ twins with a median age of 49 years (95% range: 41–67 years), of which 128 were females and 134 were males.

This study was approved by the Regional Ethics Committee of the Qingdao CDC Institutional Review Boards. Prior written informed consent was obtained from all participants. The ethical principles of Helsinki Declaration were followed.

### 2.1. Audiometric Examination

Audiometric examination was performed following the method described in a previous study [16]. In brief, the twins underwent otoscopy, and then, the pure-tone air-conducted hearing thresholds in each ear were separately measured at 0.5, 1.0, 2.0, 4.0, and 8.0 kHz by using a diagnostic audiometer. BEHL was then calculated as the lower value of both ears at each frequency. Finally, the PTA at 0.5, 1.0, 2.0, 4.0, and 8.0 kHz was separately calculated for the left and the right ear, and the better ear (i.e., the one with the lower value) was selected.

### 2.2. Genotyping and Quality Control

Genomic DNA was first extracted from the whole peripheral blood of the 131 DZ twins by using QIAamp DNA Blood Mini Kit (Qiagen GmbH, Hilden, Germany). Quantity and integrity of genomic DNA were then determined. Subsequently, DNA samples were genotyped on the Illumina's Infinium Omni2.5Exome-8v1.2 Bead Chip platform (Illumina, San Diego, CA, USA). Autosome and chromosome X data were analyzed. Quality control was applied using the following criteria: call rate > 0.98, minor allele frequency > 0.01, Hardy–Weinberg Equilibrium > 1 × 10^−4^, and locus missing < 0.05 according to genome-wide efficient mixed-model association (GEMMA) [22]. Linear-Mixed Models were used to test the genotype-phenotype association by using GEMMA. Genetic relationship matrix was included in the model analyses because of our twin pedigree data. Finally, a total of 1,365,315 single-nucleotide polymorphisms (SNPs) qualified for subsequent analyses.

### 2.3. Statistical Analysis

#### 2.3.1. Basic Characteristics Analysis

Descriptive statistics were computed using SPSS version 22.0. Square-root transformation for BEHLs and rank transformation for PTA were performed for normality. We first performed a normality test for basic characteristics. For those that did not conform to the normal distribution, the Mann-Whitney test was used for comparison.

#### 2.3.2. SNP-Based Analysis

The association between ARHI and SNP genotypes across the genome was tested using the GEMMA software [22]. Sex, age, educational level, and the first five principal components served as covariates in model fitting. SNPs that reached a suggestive evidence level (*P* < 1 × 10^−5^) rather than the conventional genome-wide significance level (*P* < 5 × 10^−8^) for the association were detected [23, 24]. The chromosome X-wide association study (XWAS) was used to find the possible trait association signals from chromosome X. Functional elaboration of the detected SNPs was further performed, and likely, cell types of action were predicted using the HaploReg v4.1 software [25, 26]. Enrichment results of cell type enhancers were reported (uncorrected *P* < 0.05).

#### 2.3.3. Gene-Based Analysis

Gene-based analysis was implemented using SNP-set association test via the versatile gene-based association study-2 (VEGAS2) approach, which incorporated information from a full set of GWAS summary data within one gene and accounts for linkage disequilibrium between them [27, 28]. SNPs from “1000G East Asian Population” were adopted. *P* < 0.05 was considered as nominal significance level [29].

#### 2.3.4. Pathway Enrichment Analysis

Pathway enrichment analysis was conducted using pathway scoring algorithm (PASCAL) [30, 31]. First, the location of genetic marker SNPs in the genes was determined, and the related scores of all genes in the pathway were calculated. Chi-squared or empirical scores were used to evaluate the pathway enrichment of high-scoring (possibly fused) genes, avoiding any standard binary enrichment test with inherent *P* value threshold. The pathway and its corresponding genes were selected KEGG, Reactome, and Biocarta.

## 3. Results

### 3.1. Basic Characteristics

The basic characteristics of the 131 DZ twins were summarized in Table 1. The males showed a higher moderate and high BEHLs (4.0 and 8.0 kHz) and PTA than the females (*P* < 0.001), whereas no difference was found in terms of low BEHLs (0.5 and 1.0 kHz).

### 3.2. SNP-Based Analysis

**Figure 1:**
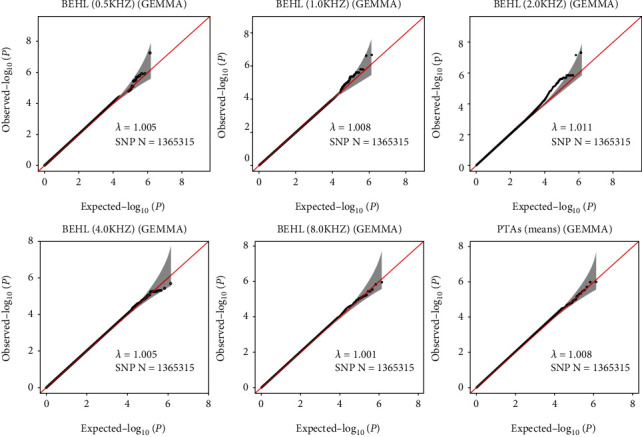
Quantile–quantile plots for GWAS of age-related hearing impairment measured by better ear hearing levels and pure tone average. The *x*-axis shows the -log10 of expected *P* values of association from Chi-square distribution, and the *y*-axis shows the -log10 of *P* values from the observed Chi-square distribution. Black dots represent the observed data with the top hit single-nucleotide polymorphism (SNP) being colored, and the red line is the expectation under the null hypothesis of no association. Gene at the best SNP is indicated.

A total of 1,365,315 SNPs genotyped from the current sample were included in the GWAS. The relationships between the observed and expected GWAS *P* values for BEHLs and PTA were illustrated in quantile–quantile (*Q*–*Q*) plots (Figure 1). The values of *λ*-statistic were close to one (0.9906–1.0110), suggesting no evidence of bias from population stratification or genomic inflation of the test statistics. The slight deviation in the upper right tail from null distribution crudely suggested some form of associations.

**Figure 2:**
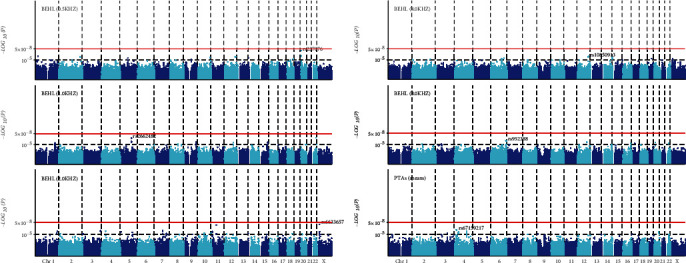
Manhattan plots for GWAS of age-related hearing impairment measured by better ear hearing levels (BEHLs) and pure tone average (PTA). The *x*-axis shows the numbers of autosomes and the X chromosome, and the *y*-axis shows the -log10 of *P* values for statistical significance. The dots represent the single-nucleotide polymorphisms (SNPs). Except for the strongest association being detected with *rs6633657* (*P* = 1.19 × 10^−8^) located on chromosome 23 for BEHL (2.0 kHz), no other SNP reached the genome-wide significance level (*P* < 5 × 10^−8^). However, several SNPs were suggestive of association (*P* < 1 × 10^−5^) for BEHLs and PTA.

As illustrated in Manhattan plots (Figure 2), *rs6633657* was the only SNP that reached the genome-wide significance level (*P* = 1.19 × 10^−8^). This SNP was located in the intron region of *PTCHD1-AS* on chromosome 23 for BEHL at 2.0 kHz. Particular for the trait association signals from chromosome X, then we ascertained by using the XWAS. By analyzing the associations of rs6633657, we identified this SNP was associated with BEHL_2.0_ (Additional file 1). No other SNP reached the genome-wide significance level (*P* < 5 × 10^−8^) for BEHLs at the other frequencies and PTA. However, 9, 10, 42, 7, and 17 SNPs were suggestive of association (*P* < 1 × 10^−5^) for BEHLs at 0.5, 1.0, 2.0, 4.0, and 8.0 kHz, respectively; by comparison, five SNPs were suggestive of association for PTA (Table 2). No consistent SNPs were observed for BEHLs at 0.5, 1.0, 2.0, 4.0, and 8.0 kHz frequencies.

**Figure 3:**
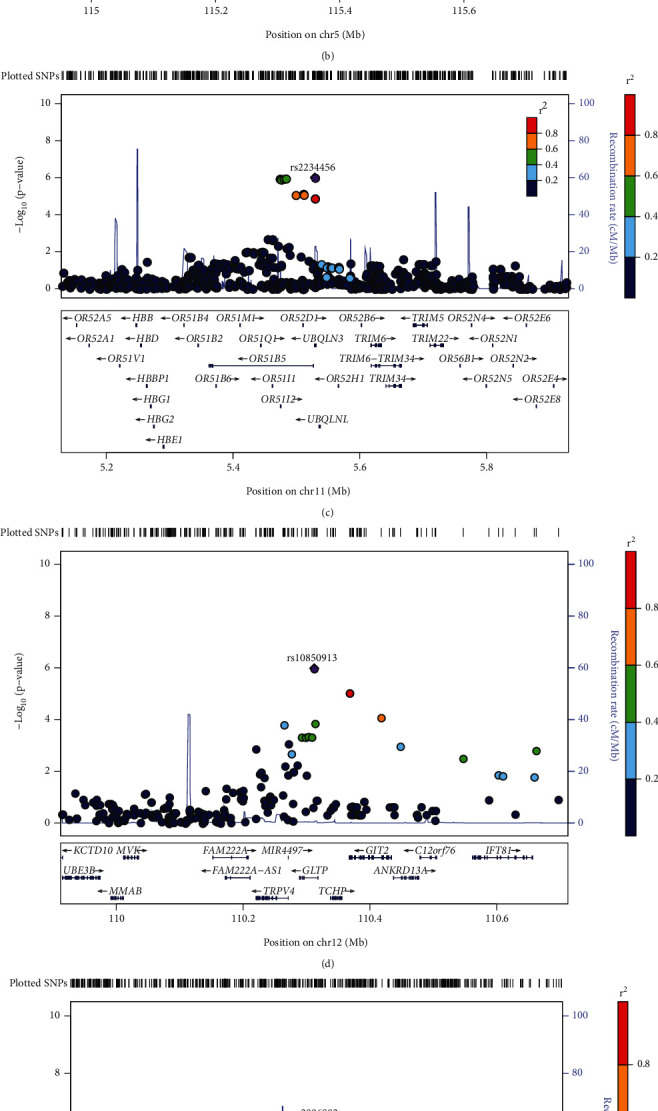
Regional association plots showing strong signals for GWAS of age-related hearing impairment measured by better ear hearing levels (BEHLs) and pure tone average (PTA). (a) BEHL_(0.5 kHz)_, (b) BEHL_(1.0 kHz)_, (c) BEHL_(2.0 kHz)_, (d) BEHL_(4.0 kHz)_, (e) BEHL_(8.0 kHz)_, and (f) PTA.

As illustrated by the regional association plots (Figure 3), several chromosomal loci showed nominal association with ARHI. Among these top signals (Table 2), three SNPs (*P* = 6.25 × 10^−7^ − 2.15 × 10^−6^) were located at or near the *C20orf196* gene on chromosome 20p12.3 for BEHL at 0.5 kHz (Figure 3(a)); four SNPs (*P* = 2.93 × 10^−7^ − 7.90 × 10^−6^) at or near the *AQPEP* gene on chromosome 5q23.1 for BEHL at 1.0 kHz (Figure 3(b)); nine SNPs (*P* = 9.81 × 10^−7^ − 8.33 × 10^−6^) at or near the *UBQLN3*, *OR51B5*, *OR51I2*, and *OR52D1* genes on chromosome 11p15.4 for BEHL at 2.0 kHz (Figure 3(c)); two SNPs (*P* = 1.05 × 10^−6^ and 9.08 × 10^−6^) at or near the *GLTP* and *GIT2* genes on chromosome 12q24.11 for BEHL at 4.0 kHz (Figure 3(d)); and six SNPs (*P* = 5.44 × 10^−7^ − 7.38 × 10^−6^) at or near the *PARK2* gene on chromosome 6q26 for BEHL at 8.0 kHz (Figure 3(e)). For PTA, two SNPs (*P* = 2.34 × 10^−6^ and 5.02 × 10^−6^) were positioned within or closest to the *GLTP* and *GIT2* genes on chromosome 12q24.11 (Figure 3(f)).

The primary T helper memory/regulatory cells from peripheral blood was identified for BEHL at 0.5 kHz by using the HaploReg v4.1 software (Additional file 2). The results were compared with meaningful ARHI-associated SNPs previously reported by other GWAS. No evidence of replication was found.

### 3.3. Gene-Based Analysis

A total of 165, 173, 77, 178, 170, and 145 genes were observed to be nominally associated with BEHLs at 0.5 kHz, 1.0 kHz, 2.0 kHz, 4.0 kHz, and 8.0 kHz and PTA, respectively (*P* < 0.05) (Additional file 3). The top 20 genes for BEHL_0.5_ ranked by *P* values are listed in Table 3 (BEHL_1.0_: Additional file 4, BEHL_2.0_: Additional file 5, BEHL_4.0_: Additional file 6, BEHL_8.0_: Additional file 7, PTA: Additional file 8). *C20orf196* gene for 0.5 kHz, S*LC16A9*, *UBQLN3*, and *OR51I2A* genes for 2.0 kHz and *FAM184A* and *TBC1D1*genes for 8.0 kHz had already been shown in suggestive level SNP-based.

These results were compared with significant ARHI-associated genes previously reported by other GWAS. Two replicable genes were identified, namely, *ACAN* [14] for BEHL at 4.0 kHz (*P* = 4.90 × 10^−2^) and *CMIP* [32] for BEHL at 8.0 kHz (*P* = 1.30 × 10^−2^). Although the well-known *GRM7* gene [12, 13] was also identified for BEHL at 8.0 kHz and PTA, the *P* values did not reach the nominal significance level.

### 3.4. Pathway Enrichment Analysis

The top 20 pathways for BEHL_0.5_ were sorted according to their empirical *P* values in Table 4 (BEHL_1.0_: Additional file 9, BEHL_2.0_: Additional file 10, BEHL_4.0_: Additional file 11, BEHL_8.0_: Additional file 12, PTA: Additional file 13). For BEHLs at 0.5, 1.0, and 2.0 kHz, the main enriched pathways were phosphatidylinositol signaling system; regulation of ornithine decarboxylase; EIF pathway; amine compound SLC transporters; synthesis of PIPS at the plasma membrane; PI metabolism; O glycan biosynthesis; transport of glucose and other sugars; and transport of bile salts and organic acids, metal ions, and amine compounds. By comparison, the main enriched pathways for BEHLs at 4.0 and 8.0 kHz were cysteine and methionine metabolism and adherens junction.

## 4. Discussion

We explored the specific genetic variants in 131 DZ twin pairs that underlie ARHI. VEGAS2 analysis suggested that several genes were nominally associated with BEHLs and PTA. Five consistent genes, namely, *C20orf196*, *GALNT9*, *INPP4B*, *SEMA7A*, and *ARID3B*, were observed for BEHLs at 0.5, 1.0, and 2.0 kHz. The *SEMA7A* gene encodes a member of the semaphorin family of proteins that have been found in activated lymphocytes and erythrocytes and which may play a crucial role in immunomodulatory and neuronal processes [33]. Although their functions in ARHI are uncertain, the other genes can also serve as latent candidates for future work. Our comparison of the ARHI-related genes found herein with those reported by previous GWAS obtained two replicable genes, namely, *ACAN* [14] for BEHL at 4.0 kHz and *CMIP* [32] for BEHL at 8.0 kHz. Using the Shared Harvard Inner Ear Database, Hoffman et al. found that *ACAN* is expressed in the auditory tissues of mouse [14]. In several developmental phases of mouse, it is mainly expressed in the cochlea and cysts, inner and outer hair cells of the cochlea, and spiral and vestibular ganglia [34–36]. By comparison, *CMIP* is expressed in the inner ear. Furthermore, Girotto et al. found that this gene is associated with hearing ability at 0.25, 1.0, and 2.0 kHz [32]. In contrast to our findings, a GWAS meta-analysis of ARHI using pure tone audiometry from multiple cohorts reported seven completely different associated loci. This may be explained by the different ethnic and genetic background [37].

Among the enriched ARHI-related pathways, amine compound SLC transporters [38]; phosphatidylinositol signaling system [39]; synthesis of PIPS at the plasma membrane [39–42]; transport of glucose and other sugars bile salts and organic acids metal ions and amine compounds [43–46]; cysteine and methionine metabolism [47, 48]; and adherens junction [49] have been previously reported to be associated with ARHI. Aside from these pathways, other pathways that may be related to ARHI were found, including EIF pathway, PI metabolism, O glycan biosynthesis, and regulation of ornithine decarboxylase. To the best of our knowledge, regulation of ornithine decarboxylase had not been reported as associated with ARHI. Ornithine decarboxylase is a key enzyme in the process of polyamine anabolism in the human body. Polyamines have various biological functions, such as antioxidation, free radical scavenging, and intracellular calcium regulation, all of which reportedly have an impact on hearing [50, 51]. Accumulating evidence shows that ornithine decarboxylase is associated with disordered cell growth regulation [52–54]. Aside from this pathway, PI metabolism had not been reported to be associated with ARHI. With the discovery of the high expression of the *TRPM7* gene in the organ of Corti and cochlea, as well as the detection of *TRPM4* immunoreactivity in the inner ear, researchers gradually realized that the TRP channel plays an important role in auditory functions [55–57]. However, TRP channels require PI metabolism to be activated [58]. Therefore, PI metabolism is also closely related to the production of hearing.

We also measured these variants by BEHLs and PTA via GWAS. Several SNPs were found to be suggestively associated with ARHI. We compared these SNPs with significant ARHI-associated SNPs previously reported by other GWAS [12–14, 32, 59]. Nevertheless, we found several promising genetic regions on chromosomes that were nominally associated with ARHI. The association between the genes involved in these promising genetic regions and ARHI could serve as candidates for further research and validation. Furthermore, the enhancer of primary T helper memory/regulatory cells from the peripheral blood for BEHL at 0.5 kHz was found. Genes involved in immunity and apoptosis are probably related to ARHI [60, 61], and the maintenance of systemic immune functions can prevent accelerated ARHI [62]. Hence, T helper memory/regulatory cells may serve as candidate tissues for further investigation of gene expression in animal models.

Investigations into genetically related individuals, such as twins, will enhance genetic association studies, and the use of twin-based designs can efficiently identify both common and rare genetic variants underlying complex traits or diseases. We conducted this GWAS on ARHI in a sample of middle and old-aged Chinese twins, and the utilization of twin-based design will empower genetic association studies and efficiently identify genetic variants underlying ARHI. However, the present GWAS has several limitations. First, owing to the challenges in recruiting and confirming qualified twin participants, we obtained a relatively small sample size. Thus, a GWAS meta-analysis with a larger sample is warranted. In addition, we could not distinguish sensorineural hearing loss from conductive hearing loss because bone conduction test was not performed in this study. Finally, lack of replication of identified signals was performed.

## 5. Conclusions

We identified lists of SNPs reached the suggestive evidence level and found several promising genetic regions on chromosomes associated with ARHI measured by BEHLs and PTA. And sets of genes nominally associated with ARHI were involved in significant biological pathways potentially related to pathogenesis of auditory development and hearing impairment. Nevertheless, the potential candidate biomarkers of ARHI reported here should merit further verifications.

## Figures and Tables

**Table 1:** Descriptive statistics for dizygotic twin pairs by gender.

Traits^#^	Male (*n* = 134)	Female (*n* = 128)	All (*n* = 262)
Age, years	50.00 (41.00-67.80)	49.00 (41.00-68.40)	49.00 (41.00-67.28)
*BEHL, dB*			
0.5 kHz	20.00 (5.00-43.00)	20.00 (5.00-40.00)	20.00 (5.00-40.00)
1.0 kHz	15.00 (0.00-38.00)	15.00 (0.00-34.00)	15.00 (0.00-35.00)
2.0 kHz	10.00 (0.00-51.00)	10.00 (0.00-34.00)	10.00 (0.00-35.00)
4.0 kHz	25.00 (2.00-78.00)	15.00 (0.00-50.00)^∗^	15.00 (0.00-70.00)
8.0 kHz	25.00 (5.00-83.00)	15.00 (0.00-60.00)^∗^	20.00 (0.00-77.13)
PTA	20.00 (7.00-46.00)	15.00 (3.20-38.60)^∗^	17.00 (5.00-43.85)

BEHL: better ear hearing level; PTA: pure tone average ^∗^*P* < 0.001^#^traits were described as median (2.5%-97.5% quantiles).

**Table 2:** Summary of SNPs associated with ARHI measured by BEHLs and PTA in GWAS (*P* value < 1 × 10^−5^).

Traits	SNP	CHR	BP	*P* value	Closest genes or genes
BEHL, by frequency (kHz)					

0.5	rs237076^#^	20	5810831	6.25*E*-07	*C20orf196*
	rs6790988	3	170263320	1.80*E*-06	*CLDN11*
	rs55705402^∗^	20	5828630	2.15*E*-06	*C20orf196*
	rs55827353^∗^	20	5826245	2.15*E*-06	*C20orf196*
	kgp8299849 (rs77570135)	10	123472528	2.84*E*-06	*LOC440700*
	rs10753110	1	175869554	4.68*E*-06	*LOC107985228*
	rs9293725	5	77177468	5.48*E*-06	*LOC101929154*
	rs1908968	4	143268702	7.89*E*-06	*INPP4B*
	rs12194558	6	153106506	9.11*E*-06	*LOC105378065*

1.0	rs2662482^#^	5	115358663	2.93*E*-07	*LVRN* (also known as *AQPEP*)
	kgp928895 (rs201156432)	22	43742432	3.35*E*-07	*SCUBE1*
	rs2560690^∗^	5	115354702	4.07*E*-06	*LVRN* (also known as *AQPEP*)
	rs2662464^∗^	5	115356017	4.07*E*-06	*LVRN* (also known as *AQPEP*)
	rs73166130	22	43746132	4.17*E*-06	*SCUBE1*
	rs35577903	1	209277772	5.68*E*-06	*LOC105372895*; *LOC107985255*
	rs9460076	6	170517603	7.49*E*-06	*RPL12P23*
	rs11106865	12	93515552	7.73*E*-06	*LOC643339*
	rs2560687∗	5	115360342	7.90*E*-06	*LVRN* (also known as *AQPEP*)
	rs11108973	12	97721824	1.01*E*-05	*LINC02409*

2.0	rs6633657	23	22836669	1.19*E*-08	*PTCHD1-AS*
	rs1974517	23	22837661	6.64*E*-07	*PTCHD1-AS*
	rs2234456^#^	11	5529139	9.81*E*-07	*UBQLN3*
	rs1498482^∗^	11	5483235	1.08*E*-06	*OR51B5*
	rs10838135^∗^	11	5473913	1.11*E*-06	*OR51B5; OR51I2*
	rs12420260^∗^	11	5473418	1.11*E*-06	*OR51B5; OR51I2*
	rs1603776∗	11	5479891	1.11*E*-06	*OR51B5*
	rs7801592	7	85187817	1.13*E*-06	*LINC00972*
	rs66808307	7	85199500	1.13*E*-06	*LINC00972*
	rs73189269	7	85199160	1.13*E*-06	*LINC00972*
	rs112837279	7	85203112	1.13*E*-06	*LINC00972*
	rs11037503^∗^	11	5475597	1.23*E*-06	*OR51B5; OR51I2*
	rs5029981	3	186438314	1.47*E*-06	*KNG1*
	rs6950989	7	85197091	1.52*E*-06	*LINC00972*
	kgp22739604 (rs6629497)	23	22846422	1.53*E*-06	*PTCHD1-AS*
	rs630428	4	38121363	1.64*E*-06	*TBC1D1*
	rs17584191	5	125533130	1.79*E*-06	*LINC02039*
	rs3763747	10	61412335	1.94*E*-06	*SLC16A9*
	kgp22746837 (rs12688139)	23	22834788	2.04*E*-06	*PTCHD1-AS*
	rs2242206	10	61414011	3.36*E*-06	*SLC16A9*
	rs1007490	23	22835253	3.73*E*-06	*PTCHD1-AS*
	rs12412363	10	130571475	4.23*E*-06	*LOC105378555*
	rs1972703	3	186463343	5.49*E*-06	*KNG1*
	kgp9174891 (rs201551669)	5	125549376	6.32*E*-06	*LINC02039*
	rs3827672	9	115924811	6.50*E*-06	*SLC31A2*
	rs12106130	20	15616247	6.65*E*-06	*MACROD2*
	rs4948351	10	61425189	6.70*E*-06	*SLC16A9*
	kgp22769801 (rs6629500)	23	22847169	6.77*E*-06	*PTCHD1-AS*
	rs17160047	7	85203213	6.88*E*-06	*LINC00972*
	rs7729369	5	125544313	6.92*E*-06	*LINC02039*
	rs7101919^∗^	11	5510688	7.30*E*-06	*OR51B5*; *OR52D1*
	rs10826342	10	61433292	7.40*E*-06	*SLC16A9*
	rs1171606	10	61434519	7.40*E*-06	*SLC16A9*
	kgp1872947 (rs199963695)	8	96237951	7.64*E*-06	*LINC01298*
	rs7728158	5	125602203	7.88*E*-06	*LOC101927488*
	rs6135472	20	15618014	7.98*E*-06	*MACROD2*
	rs4638331^∗^	11	5511431	8.21*E*-06	*OR51B5*; *OR52D1*
	rs72881227^∗^	11	5498649	8.33*E*-06	*OR51B5*
	rs4509290	8	96237596	8.49*E*-06	*LINC01298*
	rs56394481	13	110019360	8.53*E*-06	*LOC105370359*
	rs4293213	12	23923620	9.62*E*-06	*SOX5*
	rs11592061	10	3132945	9.70*E*-06	*PFKP*

4.0	rs10850913^#^	12	110312232	1.05*E*-06	GLTP
	rs35515683	6	113848824	5.20*E*-06	*LINC02541*
	rs10872099	6	113926484	5.39*E*-06	*LINC02541*
	rs80050647	10	7877888	5.40*E*-06	*TAF3*
	rs7145420	14	101915881	7.77*E*-06	*LINC02314*
	kgp3400527 (rs79297719)	10	91681180	8.39*E*-06	*LINC01375; LOC105378425*

8.0	rs2292354^∗^	12	110368201	9.08*E*-06	*GIT2*
	rs2096982^#^	6	162660989	5.44*E*-07	*PARK2*
	rs952388^∗^	6	162716541	7.04*E*-07	*PARK2*
	rs2309938	2	101699594	1.01*E*-06	*TBC1D8*
	rs13388167	2	101724667	1.89*E*-06	*TBC1D8*
	rs718772	22	30504207	2.61*E*-06	*HORMAD2*
	rs9625919	22	30500958	2.84*E*-06	*HORMAD2*
	rs11080090	17	27502029	3.70*E*-06	*MYO18A*
	rs10945825^∗^	6	162809009	4.27*E*-06	*PARK2*
	rs10945826^∗^	6	162811902	4.31*E*-06	*PARK2*
	rs10455904^∗^	6	162820082	4.31*E*-06	*PARK2*
	rs2247304	12	96424665	4.36*E*-06	*LTA4H*
	rs12598984	16	82521687	6.10*E*-06	*LOC101928392*
	rs12143791	1	178071170	6.55*E*-06	*RASAL2*
	rs10455908^∗^	6	162843312	7.38*E*-06	*PARK2*
	rs7745460	6	119396199	7.79*E*-06	*FAM184A*
	rs10871427	16	82521530	8.35*E*-06	*LOC101928392*
	rs2357161	6	119412952	8.63*E*-06	*FAM184A*

PTA	rs10850913^#^	12	110312232	2.34*E*-06	*GLTP*
	rs4565962	12	110264903	2.57*E*-06	*TRPV4*
	rs2292354^∗^	12	110368201	5.02*E*-06	*GIT2*
	rs11931969	4	22235368	5.07*E*-06	*LOC100505912*
	rs2309938	2	101699594	9.56*E*-06	*TBC1D8*

kgp: 1000 genomes project; ^#^: represented the top signals illustrated in the regional association plots for BEHLs at each frequency and for PTA; ^∗^: represented the SNPs showing linkage disequilibrium (LD) with the top signals for BEHLs at each frequency and for PTA.

**Table 3:** Top 20 genes from VEGAS2 gene-based analysis showing the strongest association with BEHL_0.5_.

Chr	Gene	nSNPs	Start position	Stop position	Gene-based test statistic	*P* value	Top-SNP	Top-SNP *P* value
17	*RNASEK-C17orf49*	6	6915735	6920843	70.85	2.00*E*-05	rs7338	8.40*E*-05
17	*C17orf49*	5	6918055	6920843	55.38	2.20*E*-05	rs14309	1.70*E*-04
20	*C20orf196*	72	5731042	5844559	273.82	6.00*E*-05	rs237076	6.20*E*-07
17	*MIR497HG*	3	6919136	6922973	30.88	1.50*E*-04	rs11078662	2.20*E*-04
19	*MYO1F*	26	8585673	8642331	115.33	2.80*E*-04	rs3213834	2.60*E*-05
16	*ITGAM*	11	31271287	31344213	135.61	3.70*E*-04	rs4594268	9.00*E*-05
3	*SLC7A14*	64	170177341	170303863	263.01	5.10*E*-04	rs6790988	1.80*E*-06
12	*CAPS2*	28	75669758	75784702	157.74	5.20*E*-04	rs12367329	8.10*E*-05
7	*SAMD9*	14	92728825	92747336	75.2	5.60*E*-04	rs76427362	4.80*E*-03
4	*MGARP*	7	140187316	140201492	37.55	5.80*E*-04	rs13120574	1.20*E*-03
12	*GLIPR1L1*	8	75728462	75764169	48.74	6.10*E*-04	rs12367329	8.10*E*-05
22	*MLC1*	24	50497819	50524358	132.45	6.10*E*-04	rs5771144	1.10*E*-04
18	*RBFA*	7	77794345	77810652	40.58	6.20*E*-04	rs3744873	1.60*E*-03
23	*GPR50*	2	150345055	150349937	14.76	6.50*E*-04	rs2072621	3.30*E*-03
14	*GPR132*	8	105515725	105531887	34.35	6.70*E*-04	rs7147439	3.40*E*-03
15	*STARD9*	66	42867856	43013196	253.11	6.80*E*-04	rs61192504	6.40*E*-04
11	*LGR4*	38	27387507	27494334	173.89	7.20*E*-04	rs11029994	9.10*E*-04
12	*GLIPR1*	7	75874512	75895716	44.38	7.50*E*-04	rs11180546	1.40*E*-04
18	*FLJ44087*	44	43018148	43087001	211.76	8.40*E*-04	rs72912678	5.30*E*-05
18	*LINC-ROR*	8	54721803	54739350	45.04	8.60*E*-04	rs1942348	8.10*E*-04

**Table 4:** Top 20 KEGG, Reactome, and Biocarta (emp-*P* < 0.05) pathway results for BEHL_0.5_ in the typed GWAS data.

Pathway	Chisq-*P*	Emp-*P*	Log (chisq*P*)	Log (emp*P*)
KEGG_PHOSPHATIDYLINOSITOL_SIGNALING_SYSTEM	8.42*E*-05	6.60*E*-06	4.07483	5.18046
REACTOME_PHOSPHOLIPID_METABOLISM	4.90*E*-04	1.86*E*-04	3.30987	3.73049
REACTOME_DIABETES_PATHWAYS	4.59*E*-04	6.40*E*-04	3.33838	3.19382
REACTOME_REGULATION_OF_ORNITHINE_DECARBOXYLASE_ODC	2.49*E*-03	1.53*E*-03	2.60460	2.81531
REACTOME_UNWINDING_OFDNA	3.69*E*-03	1.75*E*-03	2.43340	2.75696
REACTOME_SYNTHESIS_OF_DNA	3.69*E*-03	1.98*E*-03	2.43340	2.70333
KEGG_ARACHIDONIC_ACID_METABOLISM	1.56*E*-03	2.04*E*-03	2.80687	2.69037
REACTOME_DNA_STRAND_ELONGATION	3.69*E*-03	2.23*E*-03	2.43340	2.65170
BIOCARTA_EDG1_PATHWAY	2.35*E*-03	2.50*E*-03	2.62907	2.60206
REACTOME_REGULATION_OF_GENE_EXPRESSION_IN_BETA_CELLS	2.53*E*-03	2.56*E*-03	2.59605	2.59176
REACTOME_METABOLISM_OF_POLYAMINES	2.78*E*-03	2.61*E*-03	2.55570	2.58336
KEGG_MATURITY_ONSET_DIABETES_OF_THE_YOUNG	2.53*E*-03	2.65*E*-03	2.59605	2.57675
BIOCARTA_EIF_PATHWAY	3.68*E*-03	2.66*E*-03	2.43389	2.57512
BIOCARTA_CD40_PATHWAY	8.21*E*-03	2.85*E*-03	2.08554	2.54516
KEGG_RIG_I_LIKE_RECEPTOR_SIGNALING_PATHWAY	8.21*E*-03	3.04*E*-03	2.08554	2.51713
REACTOME_RIG_I_MDA5_MEDIATED_INDUCTION_OF_IFN_ALPHA_BETA_PATHWAYS	8.21*E*-03	3.14*E*-03	2.08554	2.50307
REACTOME_DESTABILIZATION_OF_MRNA_BY_KSRP	1.86*E*-03	3.19*E*-03	2.73138	2.49621
REACTOME_EFFECTS_OF_PIP2_HYDROLYSIS	7.77*E*-03	3.19*E*-03	2.10930	2.49621
REACTOME_AMINE_COMPOUND_SLC_TRANSPORTERS	3.22*E*-03	3.20*E*-03	2.49276	2.49485
BIOCARTA_AT1R_PATHWAY	7.46*E*-03	3.21*E*-03	2.12734	2.49349

## Data Availability

The SNPs datasets for this study have been deposited in the European Variation Archive (EVA) (Accession No. PRJEB23749).

## Supplementary Materials

Supplementary 1 Additional file 1: summarised results for chromosome X SNP rs6633657 with trait BEHL 2.0 kHz.

Supplementary 2 Additional file 2: query SNP enhancer summary for BEHLs and PTA.

Supplementary 3 Additional file 3: the genes nominally associated with BEHLs at each frequency and PTA from VEGAS2 gene-based analysis (*P* < 0.05).

Supplementary 4 Additional file 4: top 20 genes from VEGAS2 gene-based analysis showing the strongest association with BEHL_1.0_.

Supplementary 5 Additional file 5: top 20 genes from VEGAS2 gene-based analysis showing the strongest association with BEHL_2.0_.

Supplementary 6 Additional file 6: top 20 genes from VEGAS2 gene-based analysis showing the strongest association with BEHL_4.0_.

Supplementary 7 Additional file 7: top 20 genes from VEGAS2 gene-based analysis showing the strongest association with BEHL_8.0_.

Supplementary 8 Additional file 8: top 20 genes from VEGAS2 gene-based analysis showing the strongest association with PTA.

Supplementary 9 Additional file 9: top 20 KEGG, Reactome, and Biocarta pathway results for BEHL_1.0_ in the typed GWAS data.

Supplementary 10 Additional file 10: top 20 KEGG, Reactome, and Biocarta pathway results for BEHL_2.0_ in the typed GWAS data.

Supplementary 11 Additional file 11: top 20 KEGG, Reactome, and Biocarta pathway results for BEHL_4.0_ in the typed GWAS data.

Supplementary 12 Additional file 12: top 20 KEGG, Reactome, and Biocarta pathway results for BEHL_8.0_ in the typed GWAS data.

Supplementary 13 Additional file 13: top 20 KEGG, Reactome, and Biocarta pathway results for PTA in the typed GWAS data.
