# Supplementary material for: A Genome-Wide Association Study of Age-Related Hearing Impairment in Middle- and Old-Aged Chinese Twins
Source: Biomed Res Int. 2021 Jul 17;2021:3629624. doi: 10.1155/2021/3629624 (PMC8314043; doi:10.1155/2021/3629624)
Supplement: Supplementary 1 — Additional file 1: summarised results for chromosome X SNP rs6633657 with trait BEHL 2.0 kHz. [file 3629624.f1.docx]

**Additional file 1** Summarized results for chromosome X SNP rs6633657 with trait BEHL 2.0kHz

| XWAS |  | CHR | SNP | BP | TEST | *P* |
| --- | --- | --- | --- | --- | --- | --- |
| sex-difference test | Male | 23 | rs6633657 | 22836669 | SexDiff | 1.33E-10 |
|  | Female | 23 | rs6633657 | 22836669 | SexDiff | 0.8011 |
|  | Total | 23 | rs6633657 | 22836669 | SexDiff | 3.85E-07 |
| variance-heterogeneity test |  | 23 | rs6633657 | 22836669 | variance-heterogeneity test | 0.327358 |
| linear regression using 0/1 coding |  | 23 | rs6633657 | 22836669 | ADD | 1.09E-05 |
| for males |  | 23 | rs6633657 | 22836669 | SEX | 0.002135 |
| linear regression using 0/2 coding |  | 23 | rs6633657 | 22836669 | ADD | 3.60E-09 |
| for males |  | 23 | rs6633657 | 22836669 | SEX | 0.9356 |
