# Supplementary material for: A Genome-Wide Association Study of Age-Related Hearing Impairment in Middle- and Old-Aged Chinese Twins
Source: Biomed Res Int. 2021 Jul 17;2021:3629624. doi: 10.1155/2021/3629624 (PMC8314043; doi:10.1155/2021/3629624)
Supplement: Supplementary 2 — Additional file 2: query SNP enhancer summary for BEHLs and PTA. [file 3629624.f2.docx]

**Supplementary Table S2-1**. Query SNP enhancer summary for BEHL at the frequency of 0.5 kHz

| Cell | Observed | Expected | Expected | Binomial *P* | Binomial *P* |
| --- | --- | --- | --- | --- | --- |
|  |  | (all SNPs) | (GWAS SNPs) | (all SNPs) | (GWAS SNPs) |
| E017 LNG.IMR90 (IMR90 fetal lung fibroblasts Cell Line) | 1 | 0.4 | 0.7 | 0.332083 | 0.535895 |
| E002 ESC.WA7 (ES-WA7 Cells) | 0 | 0.1 | 0.2 | 1 | 1 |
| E008 ESC.H9 (H9 Cells) | 0 | 0.2 | 0.3 | 1 | 1 |
| E001 ESC.I3 (ES-I3 Cells) | 0 | 0.3 | 0.6 | 1 | 1 |
| E015 ESC.HUES6 (HUES6 Cells) | 0 | 0.4 | 0.5 | 1 | 1 |
| E014 ESC.HUES48 (HUES48 Cells) | 0 | 0.3 | 0.5 | 1 | 1 |
| E016 ESC.HUES64 (HUES64 Cells) | 0 | 0.3 | 0.5 | 1 | 1 |
| E003 ESC.H1 (H1 Cells) | 0 | 0.3 | 0.5 | 1 | 1 |
| E024 ESC.4STAR (ES-UCSF4 Cells) | 0 | 0.4 | 0.6 | 1 | 1 |
| E020 IPSC.20B (iPS-20b Cells) | 0 | 0.3 | 0.4 | 1 | 1 |
| E019 IPSC.18 (iPS-18 Cells) | 0 | 0.3 | 0.5 | 1 | 1 |
| E018 IPSC.15b (iPS-15b Cells) | 0 | 0.3 | 0.5 | 1 | 1 |
| E021 IPSC.DF.6.9 (iPS DF 6.9 Cells) | 0 | 0.1 | 0.3 | 1 | 1 |
| E022 IPSC.DF.19.11 (iPS DF 19.11 Cells) | 0 | 0.3 | 0.6 | 1 | 1 |
| E007 ESDR.H1.NEUR.PROG (H1 Derived Neuronal Progenitor Cultured Cells) | 0 | 0.2 | 0.4 | 1 | 1 |
| E009 ESDR.H9.NEUR.PROG (H9 Derived Neuronal Progenitor Cultured Cells) | 0 | 0.3 | 0.4 | 1 | 1 |
| E010 ESDR.H9.NEUR (H9 Derived Neuron Cultured Cells) | 0 | 0.3 | 0.5 | 1 | 1 |
| E013 ESDR.CD56.MESO (hESC Derived CD56+ Mesoderm Cultured Cells) | 0 | 0.3 | 0.6 | 1 | 1 |
| E012 ESDR.CD56.ECTO (hESC Derived CD56+ Ectoderm Cultured Cells) | 0 | 0.3 | 0.5 | 1 | 1 |
| E011 ESDR.CD184.ENDO (hESC Derived CD184+ Endoderm Cultured Cells) | 1 | 0.3 | 0.5 | 0.253376 | 0.426354 |
| E004 ESDR.H1.BMP4.MESO (H1 BMP4 Derived Mesendoderm Cultured Cells) | 0 | 0.2 | 0.3 | 1 | 1 |
| E005 ESDR.H1.BMP4.TROP (H1 BMP4 Derived Trophoblast Cultured Cells) | 0 | 0.3 | 0.6 | 1 | 1 |
| E006 ESDR.H1.MSC (H1 Derived Mesenchymal Stem Cells) | 0 | 0.4 | 0.6 | 1 | 1 |
| E062 BLD.PER.MONUC.PC (Primary mononuclear cells from peripheral blood) | 0 | 0.1 | 0.2 | 1 | 1 |
| E034 BLD.CD3.PPC (Primary T cells from peripheral blood) | 1 | 0.3 | 0.5 | 0.274774 | 0.407766 |
| E045 BLD.CD4.CD25I.CD127.TMEMPC (Primary T cells effector/memory enriched from peripheral blood) | 1 | 0.1 | 0.2 | 0.127178 | 0.222839 |
| E033 BLD.CD3.CPC (Primary T cells from cord blood) | 1 | 0.2 | 0.4 | 0.201029 | 0.307755 |
| E044 BLD.CD4.CD25.CD127M.TREGPC (Primary T regulatory cells from peripheral blood) | 2 | 0.2 | 0.3 | 0.012778 | 0.039727 |
| E043 BLD.CD4.CD25M.TPC (Primary T helper cells from peripheral blood) | 2 | 0.3 | 0.5 | 0.034962 | 0.084824 |
| E039 BLD.CD4.CD25M.CD45RA.NPC (Primary T helper naive cells from peripheral blood) | 1 | 0.3 | 0.4 | 0.241345 | 0.354422 |
| E041 BLD.CD4.CD25M.IL17M.PL.TPC (Primary T helper cells PMA-I stimulated) | 2 | 0.3 | 0.5 | 0.038148 | 0.083835 |
| E042 BLD.CD4.CD25M.IL17P.PL.TPC (Primary T helper 17 cells PMA-I stimulated) | 2 | 0.2 | 0.4 | 0.022645 | 0.069494 |
| E040 BLD.CD4.CD25M.CD45RO.MPC (Primary T helper memory cells from peripheral blood 1) | 3 | 0.3 | 0.4 | 0.001741 | 0.00788 |
| E037 BLD.CD4.MPC (Primary T helper memory cells from peripheral blood 2) | 3 | 0.3 | 0.5 | 0.002415 | 0.010216 |
| E048 BLD.CD8.MPC (Primary T CD8+ memory cells from peripheral blood) | 1 | 0.2 | 0.4 | 0.203274 | 0.33378 |
| E038 BLD.CD4.NPC (Primary T helper naive cells from peripheral blood) | 1 | 0.2 | 0.4 | 0.215799 | 0.331451 |
| E047 BLD.CD8.NPC (Primary T CD8+ naive cells from peripheral blood) | 1 | 0.3 | 0.5 | 0.239302 | 0.391868 |
| E029 BLD.CD14.PC (Primary monocytes from peripheral blood) | 1 | 0.4 | 0.7 | 0.32928 | 0.512622 |
| E031 BLD.CD19.CPC (Primary B cells from cord blood) | 0 | 0.2 | 0.4 | 1 | 1 |
| E035 BLD.CD34.PC (Primary hematopoietic stem cells) | 1 | 0.3 | 0.5 | 0.233753 | 0.391868 |
| E051 BLD.MOB.CD34.PC.M (Primary hematopoietic stem cells G-CSF-mobilized Male) | 2 | 0.4 | 0.6 | 0.047204 | 0.121699 |
| E050 BLD.MOB.CD34.PC.F (Primary hematopoietic stem cells G-CSF-mobilized Female) | 1 | 0.4 | 0.7 | 0.316715 | 0.497443 |
| E036 BLD.CD34.CC (Primary hematopoietic stem cells short term culture) | 0 | 0.3 | 0.6 | 1 | 1 |
| E032 BLD.CD19.PPC (Primary B cells from peripheral blood) | 0 | 0.4 | 0.6 | 1 | 1 |
| E046 BLD.CD56.PC (Primary Natural Killer cells from peripheral blood) | 0 | 0.3 | 0.5 | 1 | 1 |
| E030 BLD.CD15.PC (Primary neutrophils from peripheral blood) | 0 | 0.3 | 0.4 | 1 | 1 |
| E026 STRM.MRW.MSC (Bone Marrow Derived Cultured Mesenchymal Stem Cells) | 0 | 0.3 | 0.6 | 1 | 1 |
| E049 STRM.CHON.MRW.DR.MSC (Mesenchymal Stem Cell Derived Chondrocyte Cultured Cells) | 0 | 0.4 | 0.7 | 1 | 1 |
| E025 FAT.ADIP.DR.MSC (Adipose Derived Mesenchymal Stem Cell Cultured Cells) | 1 | 0.5 | 0.9 | 0.410135 | 0.5932 |
| E023 FAT.MSC.DR.ADIP (Mesenchymal Stem Cell Derived Adipocyte Cultured Cells) | 0 | 0.4 | 0.6 | 1 | 1 |
| E052 MUS.SAT (Muscle Satellite Cultured Cells) | 0 | 0.4 | 0.6 | 1 | 1 |
| E055 SKIN.PEN.FRSK.FIB.01 (Foreskin Fibroblast Primary Cells skin01) | 0 | 0.4 | 0.7 | 1 | 1 |
| E056 SKIN.PEN.FRSK.FIB.02 (Foreskin Fibroblast Primary Cells skin02) | 0 | 0.3 | 0.5 | 1 | 1 |
| E059 SKIN.PEN.FRSK.MEL.01 (Foreskin Melanocyte Primary Cells skin01) | 0 | 0.2 | 0.4 | 1 | 1 |
| E061 SKIN.PEN.FRSK.MEL.03 (Foreskin Melanocyte Primary Cells skin03) | 1 | 0.4 | 0.6 | 0.326688 | 0.466771 |
| E057 SKIN.PEN.FRSK.KER.02 (Foreskin Keratinocyte Primary Cells skin02) | 1 | 0.3 | 0.6 | 0.299518 | 0.474356 |
| E058 SKIN.PEN.FRSK.KER.03 (Foreskin Keratinocyte Primary Cells skin03) | 1 | 0.4 | 0.6 | 0.313166 | 0.478112 |
| E028 BRST.HMEC.35 (Breast variant Human Mammary Epithelial Cells (vHMEC)) | 2 | 0.4 | 0.7 | 0.05423 | 0.1468 |
| E027 BRST.MYO (Breast Myoepithelial Primary Cells) | 0 | 0.5 | 0.9 | 1 | 1 |
| E054 BRN.GANGEM.DR.NRSPHR (Ganglion Eminence derived primary cultured neurospheres) | 0 | 0.2 | 0.4 | 1 | 1 |
| E053 BRN.CRTX.DR.NRSPHR (Cortex derived primary cultured neurospheres) | 0 | 0.3 | 0.5 | 1 | 1 |
| E112 THYM (Thymus) | 0 | 0.2 | 0.3 | 1 | 1 |
| E093 THYM.FET (Fetal Thymus) | 0 | 0.3 | 0.6 | 1 | 1 |
| E071 BRN.HIPP.MID (Brain Hippocampus Middle) | 1 | 0.3 | 0.5 | 0.271055 | 0.432434 |
| E074 BRN.SUB.NIG (Brain Substantia Nigra) | 1 | 0.3 | 0.5 | 0.2464 | 0.409857 |
| E068 BRN.ANT.CAUD (Brain Anterior Caudate) | 0 | 0.3 | 0.5 | 1 | 1 |
| E069 BRN.CING.GYR (Brain Cingulate Gyrus) | 1 | 0.3 | 0.5 | 0.232281 | 0.37669 |
| E072 BRN.INF.TMP (Brain Inferior Temporal Lobe) | 1 | 0.2 | 0.4 | 0.221213 | 0.348746 |
| E067 BRN.ANG.GYR (Brain Angular Gyrus) | 0 | 0.2 | 0.4 | 1 | 1 |
| E073 BRN.DL.PRFRNTL.CRTX (Brain Dorsolateral Prefrontal Cortex) | 1 | 0.2 | 0.4 | 0.175276 | 0.318509 |
| E070 BRN.GRM.MTRX (Brain Germinal Matrix) | 0 | 0.2 | 0.3 | 1 | 1 |
| E082 BRN.FET.F (Fetal Brain Female) | 0 | 0.2 | 0.3 | 1 | 1 |
| E081 BRN.FET.M (Fetal Brain Male) | 1 | 0.3 | 0.4 | 0.271433 | 0.363413 |
| E063 FAT.ADIP.NUC (Adipose Nuclei) | 0 | 0.4 | 0.7 | 1 | 1 |
| E100 MUS.PSOAS (Psoas Muscle) | 0 | 0.2 | 0.3 | 1 | 1 |
| E108 MUS.SKLT.F (Skeletal Muscle Female) | 0 | 0.4 | 0.7 | 1 | 1 |
| E107 MUS.SKLT.M (Skeletal Muscle Male) | 0 | 0.4 | 0.6 | 1 | 1 |
| E089 MUS.TRNK.FET (Fetal Muscle Trunk) | 0 | 0.4 | 0.7 | 1 | 1 |
| E090 MUS.LEG.FET (Fetal Muscle Leg) | 0 | 0.5 | 0.9 | 1 | 1 |
| E083 HRT.FET (Fetal Heart) | 2 | 0.5 | 0.8 | 0.074837 | 0.17855 |
| E104 HRT.ATR.R (Right Atrium) | 0 | 0.2 | 0.4 | 1 | 1 |
| E095 HRT.VENT.L (Left Ventricle) | 0 | 0.3 | 0.6 | 1 | 1 |
| E105 HRT.VNT.R (Right Ventricle) | 0 | 0.2 | 0.5 | 1 | 1 |
| E065 VAS.AOR (Aorta) | 0 | 0.1 | 0.2 | 1 | 1 |
| E078 GI.DUO.SM.MUS (Duodenum Smooth Muscle) | 0 | 0.2 | 0.3 | 1 | 1 |
| E076 GI.CLN.SM.MUS (Colon Smooth Muscle) | 1 | 0.3 | 0.5 | 0.24792 | 0.406717 |
| E103 GI.RECT.SM.MUS (Rectal Smooth Muscle) | 0 | 0.2 | 0.4 | 1 | 1 |
| E111 GI.STMC.MUS (Stomach Smooth Muscle) | 1 | 0.2 | 0.4 | 0.187743 | 0.308957 |
| E092 GI.STMC.FET (Fetal Stomach) | 0 | 0.3 | 0.6 | 1 | 1 |
| E085 GI.S.INT.FET (Fetal Intestine Small) | 1 | 0.3 | 0.6 | 0.29446 | 0.46486 |
| E084 GI.L.INT.FET (Fetal Intestine Large) | 1 | 0.3 | 0.6 | 0.283505 | 0.45909 |
| E109 GI.S.INT (Small Intestine) | 1 | 0.1 | 0.2 | 0.120568 | 0.210754 |
| E106 GI.CLN.SIG (Sigmoid Colon) | 0 | 0.2 | 0.4 | 1 | 1 |
| E075 GI.CLN.MUC (Colonic Mucosa) | 0 | 0.1 | 0.2 | 1 | 1 |
| E101 GI.RECT.MUC.29 (Rectal Mucosa Donor 29) | 0 | 0.1 | 0.3 | 1 | 1 |
| E102 GI.RECT.MUC.31 (Rectal Mucosa Donor 31) | 0 | 0.2 | 0.4 | 1 | 1 |
| E110 GI.STMC.MUC (Stomach Mucosa) | 1 | 0.3 | 0.5 | 0.27549 | 0.431424 |
| E077 GI.DUO.MUC (Duodenum Mucosa) | 0 | 0.2 | 0.5 | 1 | 1 |
| E079 GI.ESO (Esophagus) | 0 | 0.2 | 0.4 | 1 | 1 |
| E094 GI.STMC.GAST (Gastric) | 0 | 0.2 | 0.3 | 1 | 1 |
| E099 PLCNT.AMN (Placenta Amnion) | 0 | 0.2 | 0.4 | 1 | 1 |
| E086 KID.FET (Fetal Kidney) | 0 | 0.2 | 0.3 | 1 | 1 |
| E088 LNG.FET (Fetal Lung) | 1 | 0.4 | 0.7 | 0.30734 | 0.497443 |
| E097 OVRY (Ovary) | 1 | 0.2 | 0.4 | 0.21435 | 0.354422 |
| E087 PANC.ISLT (Pancreatic Islets) | 1 | 0.1 | 0.2 | 0.107896 | 0.165002 |
| E080 ADRL.GLND.FET (Fetal Adrenal Gland) | 1 | 0.4 | 0.8 | 0.360828 | 0.574059 |
| E091 PLCNT.FET (Placenta) | 0 | 0.4 | 0.8 | 1 | 1 |
| E066 LIV.ADLT (Liver) | 1 | 0.3 | 0.6 | 0.298639 | 0.474356 |
| E098 PANC (Pancreas) | 0 | 0.3 | 0.5 | 1 | 1 |
| E096 LNG (Lung) | 0 | 0.2 | 0.4 | 1 | 1 |
| E113 SPLN (Spleen) | 0 | 0.3 | 0.6 | 1 | 1 |
| E114 LNG.A549.ETOH002.CNCR (A549 EtOH 0.02pct Lung Carcinoma Cell Line) | 3 | 0.3 | 0.5 | 0.002101 | 0.011316 |
| E115 BLD.DND41.CNCR (Dnd41 TCell Leukemia Cell Line) | 0 | 0.2 | 0.3 | 1 | 1 |
| E116 BLD.GM12878 (GM12878 Lymphoblastoid Cells) | 1 | 0.3 | 0.4 | 0.236771 | 0.348746 |
| E117 CRVX.HELAS3.CNCR (HeLa-S3 Cervical Carcinoma Cell Line) | 2 | 0.3 | 0.5 | 0.024983 | 0.076069 |
| E118 LIV.HEPG2.CNCR (HepG2 Hepatocellular Carcinoma Cell Line) | 3 | 0.4 | 0.7 | 0.008221 | 0.032104 |
| E119 BRST.HMEC (HMEC Mammary Epithelial Primary Cells) | 2 | 0.4 | 0.7 | 0.051583 | 0.134659 |
| E120 MUS.HSMM (HSMM Skeletal Muscle Myoblasts Cells) | 0 | 0.3 | 0.6 | 1 | 1 |
| E121 MUS.HSMMT (HSMM cell derived Skeletal Muscle Myotubes Cells) | 0 | 0.3 | 0.5 | 1 | 1 |
| E122 VAS.HUVEC (HUVEC Umbilical Vein Endothelial Primary Cells) | 1 | 0.3 | 0.5 | 0.25843 | 0.396144 |
| E123 BLD.K562.CNCR (K562 Leukemia Cells) | 1 | 0.3 | 0.4 | 0.248725 | 0.364529 |
| E124 BLD.CD14.MONO (Monocytes-CD14+ RO01746 Primary Cells) | 1 | 0.3 | 0.4 | 0.234103 | 0.364529 |
| E125 BRN.NHA (NH-A Astrocytes Primary Cells) | 0 | 0.3 | 0.5 | 1 | 1 |
| E126 SKIN.NHDFAD (NHDF-Ad Adult Dermal Fibroblast Primary Cells) | 0 | 0.4 | 0.7 | 1 | 1 |
| E127 SKIN.NHEK (NHEK-Epidermal Keratinocyte Primary Cells) | 2 | 0.3 | 0.6 | 0.045072 | 0.110205 |
| E128 LNG.NHLF (NHLF Lung Fibroblast Primary Cells) | 0 | 0.3 | 0.5 | 1 | 1 |
| E129 BONE.OSTEO (Osteoblast Primary Cells) | 0 | 0.3 | 0.6 | 1 | 1 |

**Supplementary Table S1-2.** Query SNP enhancer summary for BEHL at the frequency of 1.0 kHz

| Cell | Observed | Expected | Expected | Binomial *P* | Binomial *P* |
| --- | --- | --- | --- | --- | --- |
|  |  | (all SNPs) | (GWAS SNPs) | (all SNPs) | (GWAS SNPs) |
| E017 LNG.IMR90 (IMR90 fetal lung fibroblasts Cell Line) | 0 | 0.4 | 0.8 | 1 | 1 |
| E002 ESC.WA7 (ES-WA7 Cells) | 0 | 0.1 | 0.2 | 1 | 1 |
| E008 ESC.H9 (H9 Cells) | 0 | 0.2 | 0.3 | 1 | 1 |
| E001 ESC.I3 (ES-I3 Cells) | 0 | 0.4 | 0.6 | 1 | 1 |
| E015 ESC.HUES6 (HUES6 Cells) | 1 | 0.4 | 0.6 | 0.331104 | 0.45964 |
| E014 ESC.HUES48 (HUES48 Cells) | 1 | 0.4 | 0.5 | 0.312003 | 0.418902 |
| E016 ESC.HUES64 (HUES64 Cells) | 0 | 0.4 | 0.6 | 1 | 1 |
| E003 ESC.H1 (H1 Cells) | 1 | 0.3 | 0.6 | 0.279378 | 0.440154 |
| E024 ESC.4STAR (ES-UCSF4 Cells) | 0 | 0.4 | 0.7 | 1 | 1 |
| E020 IPSC.20B (iPS-20b Cells) | 0 | 0.3 | 0.4 | 1 | 1 |
| E019 IPSC.18 (iPS-18 Cells) | 0 | 0.4 | 0.5 | 1 | 1 |
| E018 IPSC.15b (iPS-15b Cells) | 0 | 0.4 | 0.6 | 1 | 1 |
| E021 IPSC.DF.6.9 (iPS DF 6.9 Cells) | 1 | 0.2 | 0.3 | 0.151838 | 0.298654 |
| E022 IPSC.DF.19.11 (iPS DF 19.11 Cells) | 0 | 0.4 | 0.7 | 1 | 1 |
| E007 ESDR.H1.NEUR.PROG (H1 Derived Neuronal Progenitor Cultured Cells) | 0 | 0.2 | 0.4 | 1 | 1 |
| E009 ESDR.H9.NEUR.PROG (H9 Derived Neuronal Progenitor Cultured Cells) | 0 | 0.3 | 0.5 | 1 | 1 |
| E010 ESDR.H9.NEUR (H9 Derived Neuron Cultured Cells) | 0 | 0.3 | 0.6 | 1 | 1 |
| E013 ESDR.CD56.MESO (hESC Derived CD56+ Mesoderm Cultured Cells) | 0 | 0.3 | 0.6 | 1 | 1 |
| E012 ESDR.CD56.ECTO (hESC Derived CD56+ Ectoderm Cultured Cells) | 0 | 0.3 | 0.5 | 1 | 1 |
| E011 ESDR.CD184.ENDO (hESC Derived CD184+ Endoderm Cultured Cells) | 3 | 0.3 | 0.6 | 0.003304 | 0.018738 |
| E004 ESDR.H1.BMP4.MESO (H1 BMP4 Derived Mesendoderm Cultured Cells) | 0 | 0.2 | 0.3 | 1 | 1 |
| E005 ESDR.H1.BMP4.TROP (H1 BMP4 Derived Trophoblast Cultured Cells) | 0 | 0.4 | 0.7 | 1 | 1 |
| E006 ESDR.H1.MSC (H1 Derived Mesenchymal Stem Cells) | 0 | 0.4 | 0.7 | 1 | 1 |
| E062 BLD.PER.MONUC.PC (Primary mononuclear cells from peripheral blood) | 0 | 0.1 | 0.3 | 1 | 1 |
| E034 BLD.CD3.PPC (Primary T cells from peripheral blood) | 1 | 0.4 | 0.6 | 0.300205 | 0.441253 |
| E045 BLD.CD4.CD25I.CD127.TMEMPC (Primary T cells effector/memory enriched from peripheral blood) | 0 | 0.2 | 0.3 | 1 | 1 |
| E033 BLD.CD3.CPC (Primary T cells from cord blood) | 0 | 0.2 | 0.4 | 1 | 1 |
| E044 BLD.CD4.CD25.CD127M.TREGPC (Primary T regulatory cells from peripheral blood) | 1 | 0.2 | 0.4 | 0.180666 | 0.308028 |
| E043 BLD.CD4.CD25M.TPC (Primary T helper cells from peripheral blood) | 0 | 0.3 | 0.6 | 1 | 1 |
| E039 BLD.CD4.CD25M.CD45RA.NPC (Primary T helper naive cells from peripheral blood) | 1 | 0.3 | 0.5 | 0.264274 | 0.385062 |
| E041 BLD.CD4.CD25M.IL17M.PL.TPC (Primary T helper cells PMA-I stimulated) | 0 | 0.4 | 0.5 | 1 | 1 |
| E042 BLD.CD4.CD25M.IL17P.PL.TPC (Primary T helper 17 cells PMA-I stimulated) | 0 | 0.3 | 0.5 | 1 | 1 |
| E040 BLD.CD4.CD25M.CD45RO.MPC (Primary T helper memory cells from peripheral blood 1) | 0 | 0.3 | 0.5 | 1 | 1 |
| E037 BLD.CD4.MPC (Primary T helper memory cells from peripheral blood 2) | 0 | 0.3 | 0.5 | 1 | 1 |
| E048 BLD.CD8.MPC (Primary T CD8+ memory cells from peripheral blood) | 0 | 0.2 | 0.4 | 1 | 1 |
| E038 BLD.CD4.NPC (Primary T helper naive cells from peripheral blood) | 0 | 0.3 | 0.4 | 1 | 1 |
| E047 BLD.CD8.NPC (Primary T CD8+ naive cells from peripheral blood) | 0 | 0.3 | 0.5 | 1 | 1 |
| E029 BLD.CD14.PC (Primary monocytes from peripheral blood) | 2 | 0.4 | 0.8 | 0.067249 | 0.175958 |
| E031 BLD.CD19.CPC (Primary B cells from cord blood) | 1 | 0.3 | 0.5 | 0.238259 | 0.379051 |
| E035 BLD.CD34.PC (Primary hematopoietic stem cells) | 1 | 0.3 | 0.5 | 0.256089 | 0.424564 |
| E051 BLD.MOB.CD34.PC.M (Primary hematopoietic stem cells G-CSF-mobilized Male) | 1 | 0.4 | 0.7 | 0.333362 | 0.506695 |
| E050 BLD.MOB.CD34.PC.F (Primary hematopoietic stem cells G-CSF-mobilized Female) | 1 | 0.4 | 0.7 | 0.345026 | 0.534431 |
| E036 BLD.CD34.CC (Primary hematopoietic stem cells short term culture) | 0 | 0.4 | 0.7 | 1 | 1 |
| E032 BLD.CD19.PPC (Primary B cells from peripheral blood) | 1 | 0.4 | 0.7 | 0.327754 | 0.501766 |
| E046 BLD.CD56.PC (Primary Natural Killer cells from peripheral blood) | 1 | 0.3 | 0.6 | 0.29535 | 0.45964 |
| E030 BLD.CD15.PC (Primary neutrophils from peripheral blood) | 2 | 0.3 | 0.5 | 0.033475 | 0.08355 |
| E026 STRM.MRW.MSC (Bone Marrow Derived Cultured Mesenchymal Stem Cells) | 1 | 0.4 | 0.6 | 0.300958 | 0.476446 |
| E049 STRM.CHON.MRW.DR.MSC (Mesenchymal Stem Cell Derived Chondrocyte Cultured Cells) | 1 | 0.4 | 0.8 | 0.346639 | 0.557206 |
| E025 FAT.ADIP.DR.MSC (Adipose Derived Mesenchymal Stem Cell Cultured Cells) | 1 | 0.6 | 1 | 0.443736 | 0.631889 |
| E023 FAT.MSC.DR.ADIP (Mesenchymal Stem Cell Derived Adipocyte Cultured Cells) | 0 | 0.4 | 0.7 | 1 | 1 |
| E052 MUS.SAT (Muscle Satellite Cultured Cells) | 1 | 0.4 | 0.7 | 0.332153 | 0.512553 |
| E055 SKIN.PEN.FRSK.FIB.01 (Foreskin Fibroblast Primary Cells skin01) | 1 | 0.4 | 0.8 | 0.354891 | 0.556315 |
| E056 SKIN.PEN.FRSK.FIB.02 (Foreskin Fibroblast Primary Cells skin02) | 1 | 0.3 | 0.6 | 0.25972 | 0.444538 |
| E059 SKIN.PEN.FRSK.MEL.01 (Foreskin Melanocyte Primary Cells skin01) | 0 | 0.2 | 0.5 | 1 | 1 |
| E061 SKIN.PEN.FRSK.MEL.03 (Foreskin Melanocyte Primary Cells skin03) | 0 | 0.4 | 0.7 | 1 | 1 |
| E057 SKIN.PEN.FRSK.KER.02 (Foreskin Keratinocyte Primary Cells skin02) | 1 | 0.4 | 0.7 | 0.326685 | 0.510607 |
| E058 SKIN.PEN.FRSK.KER.03 (Foreskin Keratinocyte Primary Cells skin03) | 1 | 0.4 | 0.7 | 0.341244 | 0.514491 |
| E028 BRST.HMEC.35 (Breast variant Human Mammary Epithelial Cells (vHMEC)) | 1 | 0.4 | 0.8 | 0.355069 | 0.548219 |
| E027 BRST.MYO (Breast Myoepithelial Primary Cells) | 1 | 0.5 | 1 | 0.405426 | 0.635643 |
| E054 BRN.GANGEM.DR.NRSPHR (Ganglion Eminence derived primary cultured neurospheres) | 0 | 0.3 | 0.4 | 1 | 1 |
| E053 BRN.CRTX.DR.NRSPHR (Cortex derived primary cultured neurospheres) | 0 | 0.4 | 0.6 | 1 | 1 |
| E112 THYM (Thymus) | 0 | 0.2 | 0.3 | 1 | 1 |
| E093 THYM.FET (Fetal Thymus) | 0 | 0.4 | 0.7 | 1 | 1 |
| E071 BRN.HIPP.MID (Brain Hippocampus Middle) | 0 | 0.3 | 0.6 | 1 | 1 |
| E074 BRN.SUB.NIG (Brain Substantia Nigra) | 0 | 0.3 | 0.6 | 1 | 1 |
| E068 BRN.ANT.CAUD (Brain Anterior Caudate) | 0 | 0.3 | 0.5 | 1 | 1 |
| E069 BRN.CING.GYR (Brain Cingulate Gyrus) | 0 | 0.3 | 0.5 | 1 | 1 |
| E072 BRN.INF.TMP (Brain Inferior Temporal Lobe) | 0 | 0.3 | 0.5 | 1 | 1 |
| E067 BRN.ANG.GYR (Brain Angular Gyrus) | 0 | 0.2 | 0.4 | 1 | 1 |
| E073 BRN.DL.PRFRNTL.CRTX (Brain Dorsolateral Prefrontal Cortex) | 0 | 0.2 | 0.4 | 1 | 1 |
| E070 BRN.GRM.MTRX (Brain Germinal Matrix) | 0 | 0.2 | 0.4 | 1 | 1 |
| E082 BRN.FET.F (Fetal Brain Female) | 1 | 0.2 | 0.3 | 0.16125 | 0.248618 |
| E081 BRN.FET.M (Fetal Brain Male) | 1 | 0.3 | 0.5 | 0.296623 | 0.39457 |
| E063 FAT.ADIP.NUC (Adipose Nuclei) | 1 | 0.4 | 0.8 | 0.328692 | 0.554527 |
| E100 MUS.PSOAS (Psoas Muscle) | 0 | 0.2 | 0.4 | 1 | 1 |
| E108 MUS.SKLT.F (Skeletal Muscle Female) | 1 | 0.4 | 0.7 | 0.33077 | 0.52881 |
| E107 MUS.SKLT.M (Skeletal Muscle Male) | 1 | 0.4 | 0.7 | 0.331724 | 0.526923 |
| E089 MUS.TRNK.FET (Fetal Muscle Trunk) | 1 | 0.4 | 0.8 | 0.354915 | 0.553631 |
| E090 MUS.LEG.FET (Fetal Muscle Leg) | 1 | 0.6 | 1.1 | 0.437601 | 0.672008 |
| E083 HRT.FET (Fetal Heart) | 0 | 0.5 | 0.9 | 1 | 1 |
| E104 HRT.ATR.R (Right Atrium) | 0 | 0.3 | 0.5 | 1 | 1 |
| E095 HRT.VENT.L (Left Ventricle) | 0 | 0.3 | 0.6 | 1 | 1 |
| E105 HRT.VNT.R (Right Ventricle) | 0 | 0.3 | 0.5 | 1 | 1 |
| E065 VAS.AOR (Aorta) | 0 | 0.1 | 0.2 | 1 | 1 |
| E078 GI.DUO.SM.MUS (Duodenum Smooth Muscle) | 0 | 0.2 | 0.4 | 1 | 1 |
| E076 GI.CLN.SM.MUS (Colon Smooth Muscle) | 0 | 0.3 | 0.6 | 1 | 1 |
| E103 GI.RECT.SM.MUS (Rectal Smooth Muscle) | 0 | 0.2 | 0.4 | 1 | 1 |
| E111 GI.STMC.MUS (Stomach Smooth Muscle) | 2 | 0.2 | 0.4 | 0.02078 | 0.058752 |
| E092 GI.STMC.FET (Fetal Stomach) | 0 | 0.4 | 0.7 | 1 | 1 |
| E085 GI.S.INT.FET (Fetal Intestine Small) | 1 | 0.4 | 0.7 | 0.32128 | 0.500774 |
| E084 GI.L.INT.FET (Fetal Intestine Large) | 1 | 0.4 | 0.7 | 0.309561 | 0.49479 |
| E109 GI.S.INT (Small Intestine) | 0 | 0.1 | 0.3 | 1 | 1 |
| E106 GI.CLN.SIG (Sigmoid Colon) | 0 | 0.2 | 0.4 | 1 | 1 |
| E075 GI.CLN.MUC (Colonic Mucosa) | 1 | 0.1 | 0.3 | 0.121273 | 0.229775 |
| E101 GI.RECT.MUC.29 (Rectal Mucosa Donor 29) | 0 | 0.2 | 0.3 | 1 | 1 |
| E102 GI.RECT.MUC.31 (Rectal Mucosa Donor 31) | 0 | 0.3 | 0.5 | 1 | 1 |
| E110 GI.STMC.MUC (Stomach Mucosa) | 0 | 0.4 | 0.6 | 1 | 1 |
| E077 GI.DUO.MUC (Duodenum Mucosa) | 0 | 0.3 | 0.5 | 1 | 1 |
| E079 GI.ESO (Esophagus) | 0 | 0.2 | 0.4 | 1 | 1 |
| E094 GI.STMC.GAST (Gastric) | 0 | 0.2 | 0.4 | 1 | 1 |
| E099 PLCNT.AMN (Placenta Amnion) | 0 | 0.2 | 0.5 | 1 | 1 |
| E086 KID.FET (Fetal Kidney) | 1 | 0.2 | 0.3 | 0.157195 | 0.286435 |
| E088 LNG.FET (Fetal Lung) | 0 | 0.4 | 0.7 | 1 | 1 |
| E097 OVRY (Ovary) | 0 | 0.3 | 0.5 | 1 | 1 |
| E087 PANC.ISLT (Pancreatic Islets) | 1 | 0.1 | 0.2 | 0.119141 | 0.181566 |
| E080 ADRL.GLND.FET (Fetal Adrenal Gland) | 0 | 0.5 | 0.9 | 1 | 1 |
| E091 PLCNT.FET (Placenta) | 1 | 0.5 | 0.9 | 0.374221 | 0.611804 |
| E066 LIV.ADLT (Liver) | 1 | 0.4 | 0.7 | 0.325745 | 0.510607 |
| E098 PANC (Pancreas) | 0 | 0.3 | 0.5 | 1 | 1 |
| E096 LNG (Lung) | 0 | 0.2 | 0.5 | 1 | 1 |
| E113 SPLN (Spleen) | 1 | 0.3 | 0.6 | 0.273378 | 0.479545 |
| E114 LNG.A549.ETOH002.CNCR (A549 EtOH 0.02pct Lung Carcinoma Cell Line) | 1 | 0.3 | 0.6 | 0.267358 | 0.436846 |
| E115 BLD.DND41.CNCR (Dnd41 TCell Leukemia Cell Line) | 0 | 0.2 | 0.3 | 1 | 1 |
| E116 BLD.GM12878 (GM12878 Lymphoblastoid Cells) | 2 | 0.3 | 0.5 | 0.033614 | 0.076002 |
| E117 CRVX.HELAS3.CNCR (HeLa-S3 Cervical Carcinoma Cell Line) | 0 | 0.3 | 0.5 | 1 | 1 |
| E118 LIV.HEPG2.CNCR (HepG2 Hepatocellular Carcinoma Cell Line) | 1 | 0.5 | 0.8 | 0.39935 | 0.576413 |
| E119 BRST.HMEC (HMEC Mammary Epithelial Primary Cells) | 1 | 0.4 | 0.7 | 0.347101 | 0.52881 |
| E120 MUS.HSMM (HSMM Skeletal Muscle Myoblasts Cells) | 0 | 0.3 | 0.6 | 1 | 1 |
| E121 MUS.HSMMT (HSMM cell derived Skeletal Muscle Myotubes Cells) | 0 | 0.3 | 0.6 | 1 | 1 |
| E122 VAS.HUVEC (HUVEC Umbilical Vein Endothelial Primary Cells) | 0 | 0.3 | 0.5 | 1 | 1 |
| E123 BLD.K562.CNCR (K562 Leukemia Cells) | 1 | 0.3 | 0.5 | 0.272222 | 0.395749 |
| E124 BLD.CD14.MONO (Monocytes-CD14+ RO01746 Primary Cells) | 1 | 0.3 | 0.5 | 0.256466 | 0.395749 |
| E125 BRN.NHA (NH-A Astrocytes Primary Cells) | 0 | 0.3 | 0.6 | 1 | 1 |
| E126 SKIN.NHDFAD (NHDF-Ad Adult Dermal Fibroblast Primary Cells) | 1 | 0.4 | 0.7 | 0.349445 | 0.529751 |
| E127 SKIN.NHEK (NHEK-Epidermal Keratinocyte Primary Cells) | 1 | 0.4 | 0.6 | 0.326399 | 0.485692 |
| E128 LNG.NHLF (NHLF Lung Fibroblast Primary Cells) | 0 | 0.3 | 0.5 | 1 | 1 |
| E129 BONE.OSTEO (Osteoblast Primary Cells) | 1 | 0.4 | 0.6 | 0.318884 | 0.488741 |

**Supplementary Table S2-3**. Query SNP enhancer summary for BEHL at the frequency of 2.0 kHz

| Cell | Observed | Expected | Expected | Binomial *P* | Binomial *P* |
| --- | --- | --- | --- | --- | --- |
|  |  | (all SNPs) | (GWAS SNPs) | (all SNPs) | (GWAS SNPs) |
| E017 LNG.IMR90 (IMR90 fetal lung fibroblasts Cell Line) | 4 | 1.8 | 3.4 | 0.111166 | 0.453012 |
| E002 ESC.WA7 (ES-WA7 Cells) | 3 | 0.6 | 1 | 0.019896 | 0.078873 |
| E008 ESC.H9 (H9 Cells) | 1 | 0.7 | 1.3 | 0.511717 | 0.733102 |
| E001 ESC.I3 (ES-I3 Cells) | 5 | 1.6 | 2.6 | 0.021772 | 0.112984 |
| E015 ESC.HUES6 (HUES6 Cells) | 4 | 1.7 | 2.5 | 0.08269 | 0.240594 |
| E014 ESC.HUES48 (HUES48 Cells) | 2 | 1.5 | 2.2 | 0.459363 | 0.65804 |
| E016 ESC.HUES64 (HUES64 Cells) | 4 | 1.5 | 2.4 | 0.060378 | 0.212136 |
| E003 ESC.H1 (H1 Cells) | 2 | 1.4 | 2.4 | 0.394129 | 0.693096 |
| E024 ESC.4STAR (ES-UCSF4 Cells) | 1 | 1.6 | 2.9 | 0.813533 | 0.950276 |
| E020 IPSC.20B (iPS-20b Cells) | 4 | 1.2 | 1.8 | 0.032716 | 0.10795 |
| E019 IPSC.18 (iPS-18 Cells) | 3 | 1.6 | 2.3 | 0.201982 | 0.401085 |
| E018 IPSC.15b (iPS-15b Cells) | 1 | 1.5 | 2.3 | 0.776464 | 0.910333 |
| E021 IPSC.DF.6.9 (iPS DF 6.9 Cells) | 2 | 0.7 | 1.5 | 0.150048 | 0.432784 |
| E022 IPSC.DF.19.11 (iPS DF 19.11 Cells) | 1 | 1.6 | 3 | 0.804686 | 0.954651 |
| E007 ESDR.H1.NEUR.PROG (H1 Derived Neuronal Progenitor Cultured Cells) | 3 | 0.8 | 1.7 | 0.049402 | 0.227937 |
| E009 ESDR.H9.NEUR.PROG (H9 Derived Neuronal Progenitor Cultured Cells) | 5 | 1.2 | 2.1 | 0.005986 | 0.05603 |
| E010 ESDR.H9.NEUR (H9 Derived Neuron Cultured Cells) | 3 | 1.5 | 2.5 | 0.180983 | 0.447512 |
| E013 ESDR.CD56.MESO (hESC Derived CD56+ Mesoderm Cultured Cells) | 6 | 1.3 | 2.6 | 0.002044 | 0.044556 |
| E012 ESDR.CD56.ECTO (hESC Derived CD56+ Ectoderm Cultured Cells) | 3 | 1.2 | 2.2 | 0.126761 | 0.383965 |
| E011 ESDR.CD184.ENDO (hESC Derived CD184+ Endoderm Cultured Cells) | 4 | 1.3 | 2.5 | 0.04453 | 0.242298 |
| E004 ESDR.H1.BMP4.MESO (H1 BMP4 Derived Mesendoderm Cultured Cells) | 1 | 0.8 | 1.3 | 0.532513 | 0.726592 |
| E005 ESDR.H1.BMP4.TROP (H1 BMP4 Derived Trophoblast Cultured Cells) | 4 | 1.5 | 3 | 0.063105 | 0.353959 |
| E006 ESDR.H1.MSC (H1 Derived Mesenchymal Stem Cells) | 3 | 1.6 | 3 | 0.223875 | 0.58098 |
| E062 BLD.PER.MONUC.PC (Primary mononuclear cells from peripheral blood) | 0 | 0.5 | 1.1 | 1 | 1 |
| E034 BLD.CD3.PPC (Primary T cells from peripheral blood) | 0 | 1.5 | 2.4 | 1 | 1 |
| E045 BLD.CD4.CD25I.CD127.TMEMPC (Primary T cells effector/memory enriched from peripheral blood) | 0 | 0.6 | 1.2 | 1 | 1 |
| E033 BLD.CD3.CPC (Primary T cells from cord blood) | 0 | 1 | 1.7 | 1 | 1 |
| E044 BLD.CD4.CD25.CD127M.TREGPC (Primary T regulatory cells from peripheral blood) | 0 | 0.8 | 1.5 | 1 | 1 |
| E043 BLD.CD4.CD25M.TPC (Primary T helper cells from peripheral blood) | 0 | 1.4 | 2.3 | 1 | 1 |
| E039 BLD.CD4.CD25M.CD45RA.NPC (Primary T helper naive cells from peripheral blood) | 0 | 1.3 | 2 | 1 | 1 |
| E041 BLD.CD4.CD25M.IL17M.PL.TPC (Primary T helper cells PMA-I stimulated) | 1 | 1.5 | 2.3 | 0.779533 | 0.90656 |
| E042 BLD.CD4.CD25M.IL17P.PL.TPC (Primary T helper 17 cells PMA-I stimulated) | 1 | 1.1 | 2.1 | 0.679046 | 0.880448 |
| E040 BLD.CD4.CD25M.CD45RO.MPC (Primary T helper memory cells from peripheral blood 1) | 0 | 1.2 | 2.1 | 1 | 1 |
| E037 BLD.CD4.MPC (Primary T helper memory cells from peripheral blood 2) | 0 | 1.4 | 2.3 | 1 | 1 |
| E048 BLD.CD8.MPC (Primary T CD8+ memory cells from peripheral blood) | 0 | 1 | 1.9 | 1 | 1 |
| E038 BLD.CD4.NPC (Primary T helper naive cells from peripheral blood) | 0 | 1.1 | 1.8 | 1 | 1 |
| E047 BLD.CD8.NPC (Primary T CD8+ naive cells from peripheral blood) | 0 | 1.3 | 2.3 | 1 | 1 |
| E029 BLD.CD14.PC (Primary monocytes from peripheral blood) | 2 | 1.8 | 3.2 | 0.549391 | 0.843047 |
| E031 BLD.CD19.CPC (Primary B cells from cord blood) | 2 | 1.1 | 2 | 0.311684 | 0.587805 |
| E035 BLD.CD34.PC (Primary hematopoietic stem cells) | 2 | 1.2 | 2.3 | 0.347319 | 0.667553 |
| E051 BLD.MOB.CD34.PC.M (Primary hematopoietic stem cells G-CSF-mobilized Male) | 2 | 1.7 | 2.9 | 0.501353 | 0.790476 |
| E050 BLD.MOB.CD34.PC.F (Primary hematopoietic stem cells G-CSF-mobilized Female) | 3 | 1.7 | 3.1 | 0.2519 | 0.606007 |
| E036 BLD.CD34.CC (Primary hematopoietic stem cells short term culture) | 4 | 1.6 | 2.9 | 0.074403 | 0.335879 |
| E032 BLD.CD19.PPC (Primary B cells from peripheral blood) | 4 | 1.6 | 2.8 | 0.079861 | 0.312491 |
| E046 BLD.CD56.PC (Primary Natural Killer cells from peripheral blood) | 0 | 1.4 | 2.5 | 1 | 1 |
| E030 BLD.CD15.PC (Primary neutrophils from peripheral blood) | 4 | 1.2 | 2.1 | 0.034869 | 0.15001 |
| E026 STRM.MRW.MSC (Bone Marrow Derived Cultured Mesenchymal Stem Cells) | 5 | 1.5 | 2.6 | 0.015515 | 0.120625 |
| E049 STRM.CHON.MRW.DR.MSC (Mesenchymal Stem Cell Derived Chondrocyte Cultured Cells) | 5 | 1.8 | 3.3 | 0.029687 | 0.229331 |
| E025 FAT.ADIP.DR.MSC (Adipose Derived Mesenchymal Stem Cell Cultured Cells) | 4 | 2.4 | 4 | 0.215869 | 0.575206 |
| E023 FAT.MSC.DR.ADIP (Mesenchymal Stem Cell Derived Adipocyte Cultured Cells) | 7 | 1.8 | 3 | 0.00185 | 0.028642 |
| E052 MUS.SAT (Muscle Satellite Cultured Cells) | 2 | 1.7 | 2.9 | 0.498997 | 0.798094 |
| E055 SKIN.PEN.FRSK.FIB.01 (Foreskin Fibroblast Primary Cells skin01) | 3 | 1.8 | 3.3 | 0.268484 | 0.646684 |
| E056 SKIN.PEN.FRSK.FIB.02 (Foreskin Fibroblast Primary Cells skin02) | 3 | 1.2 | 2.4 | 0.127671 | 0.432863 |
| E059 SKIN.PEN.FRSK.MEL.01 (Foreskin Melanocyte Primary Cells skin01) | 0 | 1 | 2 | 1 | 1 |
| E061 SKIN.PEN.FRSK.MEL.03 (Foreskin Melanocyte Primary Cells skin03) | 6 | 1.8 | 2.8 | 0.008758 | 0.061352 |
| E057 SKIN.PEN.FRSK.KER.02 (Foreskin Keratinocyte Primary Cells skin02) | 3 | 1.6 | 2.9 | 0.222152 | 0.560673 |
| E058 SKIN.PEN.FRSK.KER.03 (Foreskin Keratinocyte Primary Cells skin03) | 5 | 1.7 | 2.9 | 0.027641 | 0.166209 |
| E028 BRST.HMEC.35 (Breast variant Human Mammary Epithelial Cells (vHMEC)) | 4 | 1.8 | 3.2 | 0.104798 | 0.401028 |
| E027 BRST.MYO (Breast Myoepithelial Primary Cells) | 5 | 2.1 | 4 | 0.059954 | 0.377855 |
| E054 BRN.GANGEM.DR.NRSPHR (Ganglion Eminence derived primary cultured neurospheres) | 3 | 1.1 | 1.8 | 0.103752 | 0.263537 |
| E053 BRN.CRTX.DR.NRSPHR (Cortex derived primary cultured neurospheres) | 3 | 1.6 | 2.5 | 0.217336 | 0.455823 |
| E112 THYM (Thymus) | 0 | 0.7 | 1.4 | 1 | 1 |
| E093 THYM.FET (Fetal Thymus) | 2 | 1.5 | 2.9 | 0.457635 | 0.80058 |
| E071 BRN.HIPP.MID (Brain Hippocampus Middle) | 4 | 1.4 | 2.6 | 0.056239 | 0.252586 |
| E074 BRN.SUB.NIG (Brain Substantia Nigra) | 2 | 1.3 | 2.4 | 0.374706 | 0.698362 |
| E068 BRN.ANT.CAUD (Brain Anterior Caudate) | 4 | 1.2 | 2.2 | 0.032062 | 0.170864 |
| E069 BRN.CING.GYR (Brain Cingulate Gyrus) | 4 | 1.2 | 2.1 | 0.032817 | 0.166307 |
| E072 BRN.INF.TMP (Brain Inferior Temporal Lobe) | 4 | 1.2 | 2 | 0.027594 | 0.130268 |
| E067 BRN.ANG.GYR (Brain Angular Gyrus) | 1 | 1 | 1.8 | 0.639038 | 0.835655 |
| E073 BRN.DL.PRFRNTL.CRTX (Brain Dorsolateral Prefrontal Cortex) | 0 | 0.9 | 1.8 | 1 | 1 |
| E070 BRN.GRM.MTRX (Brain Germinal Matrix) | 1 | 1 | 1.6 | 0.630843 | 0.803549 |
| E082 BRN.FET.F (Fetal Brain Female) | 2 | 0.7 | 1.2 | 0.166184 | 0.332351 |
| E081 BRN.FET.M (Fetal Brain Male) | 0 | 1.5 | 2.1 | 1 | 1 |
| E063 FAT.ADIP.NUC (Adipose Nuclei) | 3 | 1.6 | 3.3 | 0.225335 | 0.643404 |
| E100 MUS.PSOAS (Psoas Muscle) | 2 | 0.9 | 1.5 | 0.223894 | 0.461998 |
| E108 MUS.SKLT.F (Skeletal Muscle Female) | 3 | 1.7 | 3 | 0.22865 | 0.595395 |
| E107 MUS.SKLT.M (Skeletal Muscle Male) | 3 | 1.7 | 3 | 0.230177 | 0.59182 |
| E089 MUS.TRNK.FET (Fetal Muscle Trunk) | 3 | 1.8 | 3.3 | 0.268526 | 0.641757 |
| E090 MUS.LEG.FET (Fetal Muscle Leg) | 4 | 2.3 | 4.4 | 0.206708 | 0.659645 |
| E083 HRT.FET (Fetal Heart) | 4 | 2.2 | 3.6 | 0.168442 | 0.495091 |
| E104 HRT.ATR.R (Right Atrium) | 2 | 1.1 | 2 | 0.287537 | 0.609471 |
| E095 HRT.VENT.L (Left Ventricle) | 3 | 1.4 | 2.6 | 0.173217 | 0.498631 |
| E105 HRT.VNT.R (Right Ventricle) | 3 | 1.1 | 2.2 | 0.104628 | 0.368915 |
| E065 VAS.AOR (Aorta) | 0 | 0.4 | 0.9 | 1 | 1 |
| E078 GI.DUO.SM.MUS (Duodenum Smooth Muscle) | 0 | 0.8 | 1.5 | 1 | 1 |
| E076 GI.CLN.SM.MUS (Colon Smooth Muscle) | 3 | 1.3 | 2.4 | 0.142334 | 0.424437 |
| E103 GI.RECT.SM.MUS (Rectal Smooth Muscle) | 1 | 0.9 | 1.7 | 0.583783 | 0.814377 |
| E111 GI.STMC.MUS (Stomach Smooth Muscle) | 3 | 1 | 1.7 | 0.070823 | 0.238308 |
| E092 GI.STMC.FET (Fetal Stomach) | 4 | 1.5 | 2.9 | 0.06349 | 0.33227 |
| E085 GI.S.INT.FET (Fetal Intestine Small) | 3 | 1.6 | 2.8 | 0.213674 | 0.541742 |
| E084 GI.L.INT.FET (Fetal Intestine Large) | 3 | 1.5 | 2.8 | 0.19577 | 0.530178 |
| E109 GI.S.INT (Small Intestine) | 2 | 0.6 | 1.1 | 0.119399 | 0.297762 |
| E106 GI.CLN.SIG (Sigmoid Colon) | 2 | 0.8 | 1.7 | 0.202599 | 0.51783 |
| E075 GI.CLN.MUC (Colonic Mucosa) | 0 | 0.5 | 1.1 | 1 | 1 |
| E101 GI.RECT.MUC.29 (Rectal Mucosa Donor 29) | 0 | 0.7 | 1.4 | 1 | 1 |
| E102 GI.RECT.MUC.31 (Rectal Mucosa Donor 31) | 3 | 1.1 | 2.1 | 0.094492 | 0.343022 |
| E110 GI.STMC.MUC (Stomach Mucosa) | 2 | 1.5 | 2.6 | 0.437416 | 0.733228 |
| E077 GI.DUO.MUC (Duodenum Mucosa) | 2 | 1.1 | 2.1 | 0.301287 | 0.630296 |
| E079 GI.ESO (Esophagus) | 3 | 0.9 | 1.7 | 0.057097 | 0.248767 |
| E094 GI.STMC.GAST (Gastric) | 0 | 0.8 | 1.5 | 1 | 1 |
| E099 PLCNT.AMN (Placenta Amnion) | 3 | 1 | 2 | 0.070968 | 0.325738 |
| E086 KID.FET (Fetal Kidney) | 1 | 0.7 | 1.4 | 0.512411 | 0.757662 |
| E088 LNG.FET (Fetal Lung) | 3 | 1.7 | 3.1 | 0.23551 | 0.606007 |
| E097 OVRY (Ovary) | 1 | 1.1 | 2 | 0.675609 | 0.870254 |
| E087 PANC.ISLT (Pancreatic Islets) | 0 | 0.5 | 0.8 | 1 | 1 |
| E080 ADRL.GLND.FET (Fetal Adrenal Gland) | 4 | 2 | 3.8 | 0.145237 | 0.534249 |
| E091 PLCNT.FET (Placenta) | 3 | 1.9 | 3.8 | 0.302044 | 0.743484 |
| E066 LIV.ADLT (Liver) | 1 | 1.6 | 2.9 | 0.808988 | 0.950276 |
| E098 PANC (Pancreas) | 2 | 1.3 | 2.2 | 0.378736 | 0.65028 |
| E096 LNG (Lung) | 1 | 1 | 2 | 0.648571 | 0.87443 |
| E113 SPLN (Spleen) | 0 | 1.3 | 2.7 | 1 | 1 |
| E114 LNG.A549.ETOH002.CNCR (A549 EtOH 0.02pct Lung Carcinoma Cell Line) | 2 | 1.3 | 2.3 | 0.369957 | 0.687757 |
| E115 BLD.DND41.CNCR (Dnd41 TCell Leukemia Cell Line) | 1 | 1 | 1.3 | 0.632503 | 0.741547 |
| E116 BLD.GM12878 (GM12878 Lymphoblastoid Cells) | 1 | 1.2 | 2 | 0.716608 | 0.864844 |
| E117 CRVX.HELAS3.CNCR (HeLa-S3 Cervical Carcinoma Cell Line) | 4 | 1.2 | 2.2 | 0.030017 | 0.172393 |
| E118 LIV.HEPG2.CNCR (HepG2 Hepatocellular Carcinoma Cell Line) | 4 | 2.1 | 3.5 | 0.154484 | 0.458329 |
| E119 BRST.HMEC (HMEC Mammary Epithelial Primary Cells) | 3 | 1.8 | 3 | 0.255357 | 0.595395 |
| E120 MUS.HSMM (HSMM Skeletal Muscle Myoblasts Cells) | 3 | 1.3 | 2.7 | 0.149386 | 0.510578 |
| E121 MUS.HSMMT (HSMM cell derived Skeletal Muscle Myotubes Cells) | 4 | 1.3 | 2.4 | 0.044005 | 0.222063 |
| E122 VAS.HUVEC (HUVEC Umbilical Vein Endothelial Primary Cells) | 1 | 1.4 | 2.3 | 0.752234 | 0.905008 |
| E123 BLD.K562.CNCR (K562 Leukemia Cells) | 3 | 1.3 | 2.1 | 0.143457 | 0.340861 |
| E124 BLD.CD14.MONO (Monocytes-CD14+ RO01746 Primary Cells) | 1 | 1.2 | 2.1 | 0.711953 | 0.879465 |
| E125 BRN.NHA (NH-A Astrocytes Primary Cells) | 4 | 1.3 | 2.4 | 0.037603 | 0.213781 |
| E126 SKIN.NHDFAD (NHDF-Ad Adult Dermal Fibroblast Primary Cells) | 4 | 1.8 | 3.1 | 0.099313 | 0.364825 |
| E127 SKIN.NHEK (NHEK-Epidermal Keratinocyte Primary Cells) | 4 | 1.6 | 2.7 | 0.078734 | 0.284012 |
| E128 LNG.NHLF (NHLF Lung Fibroblast Primary Cells) | 3 | 1.2 | 2.3 | 0.114622 | 0.396815 |
| E129 BONE.OSTEO (Osteoblast Primary Cells) | 5 | 1.6 | 2.7 | 0.020266 | 0.13429 |

**Supplementary Table S2-4**. Query SNP enhancer summary for BEHL at the frequency of 4.0 kHz

| Cell | Observed | Expected | Expected | Binomial *P* | Binomial *P* |
| --- | --- | --- | --- | --- | --- |
|  |  | (all SNPs) | (GWAS SNPs) | (all SNPs) | (GWAS SNPs) |
| E017 LNG.IMR90 (IMR90 fetal lung fibroblasts Cell Line) | 1 | 0.3 | 0.6 | 0.269411 | 0.449571 |
| E002 ESC.WA7 (ES-WA7 Cells) | 0 | 0.1 | 0.2 | 1 | 1 |
| E008 ESC.H9 (H9 Cells) | 0 | 0.1 | 0.2 | 1 | 1 |
| E001 ESC.I3 (ES-I3 Cells) | 0 | 0.3 | 0.4 | 1 | 1 |
| E015 ESC.HUES6 (HUES6 Cells) | 0 | 0.3 | 0.4 | 1 | 1 |
| E014 ESC.HUES48 (HUES48 Cells) | 0 | 0.3 | 0.4 | 1 | 1 |
| E016 ESC.HUES64 (HUES64 Cells) | 0 | 0.2 | 0.4 | 1 | 1 |
| E003 ESC.H1 (H1 Cells) | 0 | 0.2 | 0.4 | 1 | 1 |
| E024 ESC.4STAR (ES-UCSF4 Cells) | 0 | 0.3 | 0.5 | 1 | 1 |
| E020 IPSC.20B (iPS-20b Cells) | 0 | 0.2 | 0.3 | 1 | 1 |
| E019 IPSC.18 (iPS-18 Cells) | 0 | 0.3 | 0.4 | 1 | 1 |
| E018 IPSC.15b (iPS-15b Cells) | 0 | 0.2 | 0.4 | 1 | 1 |
| E021 IPSC.DF.6.9 (iPS DF 6.9 Cells) | 0 | 0.1 | 0.2 | 1 | 1 |
| E022 IPSC.DF.19.11 (iPS DF 19.11 Cells) | 0 | 0.3 | 0.5 | 1 | 1 |
| E007 ESDR.H1.NEUR.PROG (H1 Derived Neuronal Progenitor Cultured Cells) | 0 | 0.1 | 0.3 | 1 | 1 |
| E009 ESDR.H9.NEUR.PROG (H9 Derived Neuronal Progenitor Cultured Cells) | 0 | 0.2 | 0.3 | 1 | 1 |
| E010 ESDR.H9.NEUR (H9 Derived Neuron Cultured Cells) | 0 | 0.2 | 0.4 | 1 | 1 |
| E013 ESDR.CD56.MESO (hESC Derived CD56+ Mesoderm Cultured Cells) | 0 | 0.2 | 0.4 | 1 | 1 |
| E012 ESDR.CD56.ECTO (hESC Derived CD56+ Ectoderm Cultured Cells) | 0 | 0.2 | 0.4 | 1 | 1 |
| E011 ESDR.CD184.ENDO (hESC Derived CD184+ Endoderm Cultured Cells) | 0 | 0.2 | 0.4 | 1 | 1 |
| E004 ESDR.H1.BMP4.MESO (H1 BMP4 Derived Mesendoderm Cultured Cells) | 0 | 0.1 | 0.2 | 1 | 1 |
| E005 ESDR.H1.BMP4.TROP (H1 BMP4 Derived Trophoblast Cultured Cells) | 1 | 0.3 | 0.5 | 0.225745 | 0.405333 |
| E006 ESDR.H1.MSC (H1 Derived Mesenchymal Stem Cells) | 0 | 0.3 | 0.5 | 1 | 1 |
| E062 BLD.PER.MONUC.PC (Primary mononuclear cells from peripheral blood) | 0 | 0.1 | 0.2 | 1 | 1 |
| E034 BLD.CD3.PPC (Primary T cells from peripheral blood) | 0 | 0.2 | 0.4 | 1 | 1 |
| E045 BLD.CD4.CD25I.CD127.TMEMPC (Primary T cells effector/memory enriched from peripheral blood) | 0 | 0.1 | 0.2 | 1 | 1 |
| E033 BLD.CD3.CPC (Primary T cells from cord blood) | 0 | 0.2 | 0.3 | 1 | 1 |
| E044 BLD.CD4.CD25.CD127M.TREGPC (Primary T regulatory cells from peripheral blood) | 0 | 0.1 | 0.3 | 1 | 1 |
| E043 BLD.CD4.CD25M.TPC (Primary T helper cells from peripheral blood) | 0 | 0.2 | 0.4 | 1 | 1 |
| E039 BLD.CD4.CD25M.CD45RA.NPC (Primary T helper naive cells from peripheral blood) | 0 | 0.2 | 0.3 | 1 | 1 |
| E041 BLD.CD4.CD25M.IL17M.PL.TPC (Primary T helper cells PMA-I stimulated) | 0 | 0.2 | 0.4 | 1 | 1 |
| E042 BLD.CD4.CD25M.IL17P.PL.TPC (Primary T helper 17 cells PMA-I stimulated) | 0 | 0.2 | 0.3 | 1 | 1 |
| E040 BLD.CD4.CD25M.CD45RO.MPC (Primary T helper memory cells from peripheral blood 1) | 0 | 0.2 | 0.3 | 1 | 1 |
| E037 BLD.CD4.MPC (Primary T helper memory cells from peripheral blood 2) | 0 | 0.2 | 0.4 | 1 | 1 |
| E048 BLD.CD8.MPC (Primary T CD8+ memory cells from peripheral blood) | 0 | 0.2 | 0.3 | 1 | 1 |
| E038 BLD.CD4.NPC (Primary T helper naive cells from peripheral blood) | 0 | 0.2 | 0.3 | 1 | 1 |
| E047 BLD.CD8.NPC (Primary T CD8+ naive cells from peripheral blood) | 0 | 0.2 | 0.4 | 1 | 1 |
| E029 BLD.CD14.PC (Primary monocytes from peripheral blood) | 0 | 0.3 | 0.5 | 1 | 1 |
| E031 BLD.CD19.CPC (Primary B cells from cord blood) | 0 | 0.2 | 0.3 | 1 | 1 |
| E035 BLD.CD34.PC (Primary hematopoietic stem cells) | 0 | 0.2 | 0.4 | 1 | 1 |
| E051 BLD.MOB.CD34.PC.M (Primary hematopoietic stem cells G-CSF-mobilized Male) | 0 | 0.3 | 0.5 | 1 | 1 |
| E050 BLD.MOB.CD34.PC.F (Primary hematopoietic stem cells G-CSF-mobilized Female) | 0 | 0.3 | 0.5 | 1 | 1 |
| E036 BLD.CD34.CC (Primary hematopoietic stem cells short term culture) | 0 | 0.3 | 0.5 | 1 | 1 |
| E032 BLD.CD19.PPC (Primary B cells from peripheral blood) | 0 | 0.3 | 0.5 | 1 | 1 |
| E046 BLD.CD56.PC (Primary Natural Killer cells from peripheral blood) | 0 | 0.2 | 0.4 | 1 | 1 |
| E030 BLD.CD15.PC (Primary neutrophils from peripheral blood) | 0 | 0.2 | 0.3 | 1 | 1 |
| E026 STRM.MRW.MSC (Bone Marrow Derived Cultured Mesenchymal Stem Cells) | 0 | 0.2 | 0.4 | 1 | 1 |
| E049 STRM.CHON.MRW.DR.MSC (Mesenchymal Stem Cell Derived Chondrocyte Cultured Cells) | 0 | 0.3 | 0.5 | 1 | 1 |
| E025 FAT.ADIP.DR.MSC (Adipose Derived Mesenchymal Stem Cell Cultured Cells) | 0 | 0.4 | 0.7 | 1 | 1 |
| E023 FAT.MSC.DR.ADIP (Mesenchymal Stem Cell Derived Adipocyte Cultured Cells) | 1 | 0.3 | 0.5 | 0.262795 | 0.406164 |
| E052 MUS.SAT (Muscle Satellite Cultured Cells) | 1 | 0.3 | 0.5 | 0.246169 | 0.395287 |
| E055 SKIN.PEN.FRSK.FIB.01 (Foreskin Fibroblast Primary Cells skin01) | 0 | 0.3 | 0.5 | 1 | 1 |
| E056 SKIN.PEN.FRSK.FIB.02 (Foreskin Fibroblast Primary Cells skin02) | 0 | 0.2 | 0.4 | 1 | 1 |
| E059 SKIN.PEN.FRSK.MEL.01 (Foreskin Melanocyte Primary Cells skin01) | 0 | 0.2 | 0.3 | 1 | 1 |
| E061 SKIN.PEN.FRSK.MEL.03 (Foreskin Melanocyte Primary Cells skin03) | 0 | 0.3 | 0.5 | 1 | 1 |
| E057 SKIN.PEN.FRSK.KER.02 (Foreskin Keratinocyte Primary Cells skin02) | 1 | 0.3 | 0.5 | 0.241854 | 0.393598 |
| E058 SKIN.PEN.FRSK.KER.03 (Foreskin Keratinocyte Primary Cells skin03) | 1 | 0.3 | 0.5 | 0.253367 | 0.396971 |
| E028 BRST.HMEC.35 (Breast variant Human Mammary Epithelial Cells (vHMEC)) | 1 | 0.3 | 0.5 | 0.26437 | 0.426611 |
| E027 BRST.MYO (Breast Myoepithelial Primary Cells) | 2 | 0.4 | 0.7 | 0.045463 | 0.139944 |
| E054 BRN.GANGEM.DR.NRSPHR (Ganglion Eminence derived primary cultured neurospheres) | 1 | 0.2 | 0.3 | 0.174163 | 0.261899 |
| E053 BRN.CRTX.DR.NRSPHR (Cortex derived primary cultured neurospheres) | 0 | 0.3 | 0.4 | 1 | 1 |
| E112 THYM (Thymus) | 0 | 0.1 | 0.2 | 1 | 1 |
| E093 THYM.FET (Fetal Thymus) | 1 | 0.3 | 0.5 | 0.229638 | 0.396971 |
| E071 BRN.HIPP.MID (Brain Hippocampus Middle) | 0 | 0.2 | 0.4 | 1 | 1 |
| E074 BRN.SUB.NIG (Brain Substantia Nigra) | 0 | 0.2 | 0.4 | 1 | 1 |
| E068 BRN.ANT.CAUD (Brain Anterior Caudate) | 0 | 0.2 | 0.4 | 1 | 1 |
| E069 BRN.CING.GYR (Brain Cingulate Gyrus) | 0 | 0.2 | 0.4 | 1 | 1 |
| E072 BRN.INF.TMP (Brain Inferior Temporal Lobe) | 0 | 0.2 | 0.3 | 1 | 1 |
| E067 BRN.ANG.GYR (Brain Angular Gyrus) | 0 | 0.2 | 0.3 | 1 | 1 |
| E073 BRN.DL.PRFRNTL.CRTX (Brain Dorsolateral Prefrontal Cortex) | 0 | 0.1 | 0.3 | 1 | 1 |
| E070 BRN.GRM.MTRX (Brain Germinal Matrix) | 0 | 0.2 | 0.3 | 1 | 1 |
| E082 BRN.FET.F (Fetal Brain Female) | 0 | 0.1 | 0.2 | 1 | 1 |
| E081 BRN.FET.M (Fetal Brain Male) | 0 | 0.2 | 0.3 | 1 | 1 |
| E063 FAT.ADIP.NUC (Adipose Nuclei) | 0 | 0.3 | 0.5 | 1 | 1 |
| E100 MUS.PSOAS (Psoas Muscle) | 0 | 0.1 | 0.3 | 1 | 1 |
| E108 MUS.SKLT.F (Skeletal Muscle Female) | 0 | 0.3 | 0.5 | 1 | 1 |
| E107 MUS.SKLT.M (Skeletal Muscle Male) | 0 | 0.3 | 0.5 | 1 | 1 |
| E089 MUS.TRNK.FET (Fetal Muscle Trunk) | 0 | 0.3 | 0.5 | 1 | 1 |
| E090 MUS.LEG.FET (Fetal Muscle Leg) | 0 | 0.4 | 0.7 | 1 | 1 |
| E083 HRT.FET (Fetal Heart) | 1 | 0.4 | 0.6 | 0.308984 | 0.467971 |
| E104 HRT.ATR.R (Right Atrium) | 0 | 0.2 | 0.3 | 1 | 1 |
| E095 HRT.VENT.L (Left Ventricle) | 0 | 0.2 | 0.4 | 1 | 1 |
| E105 HRT.VNT.R (Right Ventricle) | 0 | 0.2 | 0.4 | 1 | 1 |
| E065 VAS.AOR (Aorta) | 0 | 0.1 | 0.2 | 1 | 1 |
| E078 GI.DUO.SM.MUS (Duodenum Smooth Muscle) | 0 | 0.1 | 0.3 | 1 | 1 |
| E076 GI.CLN.SM.MUS (Colon Smooth Muscle) | 0 | 0.2 | 0.4 | 1 | 1 |
| E103 GI.RECT.SM.MUS (Rectal Smooth Muscle) | 0 | 0.1 | 0.3 | 1 | 1 |
| E111 GI.STMC.MUS (Stomach Smooth Muscle) | 0 | 0.2 | 0.3 | 1 | 1 |
| E092 GI.STMC.FET (Fetal Stomach) | 0 | 0.3 | 0.5 | 1 | 1 |
| E085 GI.S.INT.FET (Fetal Intestine Small) | 0 | 0.3 | 0.5 | 1 | 1 |
| E084 GI.L.INT.FET (Fetal Intestine Large) | 1 | 0.3 | 0.5 | 0.228408 | 0.379945 |
| E109 GI.S.INT (Small Intestine) | 0 | 0.1 | 0.2 | 1 | 1 |
| E106 GI.CLN.SIG (Sigmoid Colon) | 0 | 0.1 | 0.3 | 1 | 1 |
| E075 GI.CLN.MUC (Colonic Mucosa) | 0 | 0.1 | 0.2 | 1 | 1 |
| E101 GI.RECT.MUC.29 (Rectal Mucosa Donor 29) | 1 | 0.1 | 0.2 | 0.108896 | 0.206157 |
| E102 GI.RECT.MUC.31 (Rectal Mucosa Donor 31) | 0 | 0.2 | 0.3 | 1 | 1 |
| E110 GI.STMC.MUC (Stomach Mucosa) | 0 | 0.2 | 0.4 | 1 | 1 |
| E077 GI.DUO.MUC (Duodenum Mucosa) | 0 | 0.2 | 0.4 | 1 | 1 |
| E079 GI.ESO (Esophagus) | 1 | 0.1 | 0.3 | 0.137212 | 0.254868 |
| E094 GI.STMC.GAST (Gastric) | 0 | 0.1 | 0.3 | 1 | 1 |
| E099 PLCNT.AMN (Placenta Amnion) | 1 | 0.2 | 0.3 | 0.14945 | 0.290425 |
| E086 KID.FET (Fetal Kidney) | 0 | 0.1 | 0.2 | 1 | 1 |
| E088 LNG.FET (Fetal Lung) | 1 | 0.3 | 0.5 | 0.248446 | 0.414416 |
| E097 OVRY (Ovary) | 1 | 0.2 | 0.3 | 0.171082 | 0.288488 |
| E087 PANC.ISLT (Pancreatic Islets) | 0 | 0.1 | 0.1 | 1 | 1 |
| E080 ADRL.GLND.FET (Fetal Adrenal Gland) | 0 | 0.3 | 0.6 | 1 | 1 |
| E091 PLCNT.FET (Placenta) | 1 | 0.3 | 0.6 | 0.279731 | 0.484373 |
| E066 LIV.ADLT (Liver) | 0 | 0.3 | 0.5 | 1 | 1 |
| E098 PANC (Pancreas) | 1 | 0.2 | 0.4 | 0.199046 | 0.312371 |
| E096 LNG (Lung) | 0 | 0.2 | 0.3 | 1 | 1 |
| E113 SPLN (Spleen) | 0 | 0.2 | 0.4 | 1 | 1 |
| E114 LNG.A549.ETOH002.CNCR (A549 EtOH 0.02pct Lung Carcinoma Cell Line) | 1 | 0.2 | 0.4 | 0.195688 | 0.33098 |
| E115 BLD.DND41.CNCR (Dnd41 TCell Leukemia Cell Line) | 0 | 0.2 | 0.2 | 1 | 1 |
| E116 BLD.GM12878 (GM12878 Lymphoblastoid Cells) | 0 | 0.2 | 0.3 | 1 | 1 |
| E117 CRVX.HELAS3.CNCR (HeLa-S3 Cervical Carcinoma Cell Line) | 1 | 0.2 | 0.4 | 0.181078 | 0.311429 |
| E118 LIV.HEPG2.CNCR (HepG2 Hepatocellular Carcinoma Cell Line) | 1 | 0.3 | 0.6 | 0.300101 | 0.4519 |
| E119 BRST.HMEC (HMEC Mammary Epithelial Primary Cells) | 2 | 0.3 | 0.5 | 0.031801 | 0.086418 |
| E120 MUS.HSMM (HSMM Skeletal Muscle Myoblasts Cells) | 1 | 0.2 | 0.4 | 0.202915 | 0.371278 |
| E121 MUS.HSMMT (HSMM cell derived Skeletal Muscle Myotubes Cells) | 0 | 0.2 | 0.4 | 1 | 1 |
| E122 VAS.HUVEC (HUVEC Umbilical Vein Endothelial Primary Cells) | 0 | 0.2 | 0.4 | 1 | 1 |
| E123 BLD.K562.CNCR (K562 Leukemia Cells) | 1 | 0.2 | 0.3 | 0.19943 | 0.297167 |
| E124 BLD.CD14.MONO (Monocytes-CD14+ RO01746 Primary Cells) | 0 | 0.2 | 0.3 | 1 | 1 |
| E125 BRN.NHA (NH-A Astrocytes Primary Cells) | 0 | 0.2 | 0.4 | 1 | 1 |
| E126 SKIN.NHDFAD (NHDF-Ad Adult Dermal Fibroblast Primary Cells) | 0 | 0.3 | 0.5 | 1 | 1 |
| E127 SKIN.NHEK (NHEK-Epidermal Keratinocyte Primary Cells) | 1 | 0.3 | 0.5 | 0.241629 | 0.372149 |
| E128 LNG.NHLF (NHLF Lung Fibroblast Primary Cells) | 1 | 0.2 | 0.4 | 0.181472 | 0.32173 |
| E129 BONE.OSTEO (Osteoblast Primary Cells) | 0 | 0.3 | 0.5 | 1 | 1 |

**Supplementary Table S2-5.** Query SNP enhancer summary for BEHL at the frequency of 8.0 kHz

| Cell | Observed | Expected | Expected | Binomial *P* | Binomial *P* |
| --- | --- | --- | --- | --- | --- |
|  |  | (all SNPs) | (GWAS SNPs) | (all SNPs) | (GWAS SNPs) |
| E017 LNG.IMR90 (IMR90 fetal lung fibroblasts Cell Line) | 0 | 0.7 | 1.4 | 1 | 1 |
| E002 ESC.WA7 (ES-WA7 Cells) | 0 | 0.2 | 0.4 | 1 | 1 |
| E008 ESC.H9 (H9 Cells) | 0 | 0.3 | 0.5 | 1 | 1 |
| E001 ESC.I3 (ES-I3 Cells) | 0 | 0.7 | 1 | 1 | 1 |
| E015 ESC.HUES6 (HUES6 Cells) | 0 | 0.7 | 1 | 1 | 1 |
| E014 ESC.HUES48 (HUES48 Cells) | 0 | 0.6 | 0.9 | 1 | 1 |
| E016 ESC.HUES64 (HUES64 Cells) | 0 | 0.6 | 1 | 1 | 1 |
| E003 ESC.H1 (H1 Cells) | 0 | 0.5 | 1 | 1 | 1 |
| E024 ESC.4STAR (ES-UCSF4 Cells) | 0 | 0.7 | 1.2 | 1 | 1 |
| E020 IPSC.20B (iPS-20b Cells) | 0 | 0.5 | 0.7 | 1 | 1 |
| E019 IPSC.18 (iPS-18 Cells) | 0 | 0.6 | 0.9 | 1 | 1 |
| E018 IPSC.15b (iPS-15b Cells) | 0 | 0.6 | 0.9 | 1 | 1 |
| E021 IPSC.DF.6.9 (iPS DF 6.9 Cells) | 0 | 0.3 | 0.6 | 1 | 1 |
| E022 IPSC.DF.19.11 (iPS DF 19.11 Cells) | 0 | 0.6 | 1.2 | 1 | 1 |
| E007 ESDR.H1.NEUR.PROG (H1 Derived Neuronal Progenitor Cultured Cells) | 1 | 0.3 | 0.7 | 0.286404 | 0.49421 |
| E009 ESDR.H9.NEUR.PROG (H9 Derived Neuronal Progenitor Cultured Cells) | 0 | 0.5 | 0.8 | 1 | 1 |
| E010 ESDR.H9.NEUR (H9 Derived Neuron Cultured Cells) | 0 | 0.6 | 1 | 1 | 1 |
| E013 ESDR.CD56.MESO (hESC Derived CD56+ Mesoderm Cultured Cells) | 0 | 0.5 | 1.1 | 1 | 1 |
| E012 ESDR.CD56.ECTO (hESC Derived CD56+ Ectoderm Cultured Cells) | 0 | 0.5 | 0.9 | 1 | 1 |
| E011 ESDR.CD184.ENDO (hESC Derived CD184+ Endoderm Cultured Cells) | 0 | 0.5 | 1 | 1 | 1 |
| E004 ESDR.H1.BMP4.MESO (H1 BMP4 Derived Mesendoderm Cultured Cells) | 0 | 0.3 | 0.5 | 1 | 1 |
| E005 ESDR.H1.BMP4.TROP (H1 BMP4 Derived Trophoblast Cultured Cells) | 0 | 0.6 | 1.2 | 1 | 1 |
| E006 ESDR.H1.MSC (H1 Derived Mesenchymal Stem Cells) | 0 | 0.7 | 1.2 | 1 | 1 |
| E062 BLD.PER.MONUC.PC (Primary mononuclear cells from peripheral blood) | 0 | 0.2 | 0.5 | 1 | 1 |
| E034 BLD.CD3.PPC (Primary T cells from peripheral blood) | 0 | 0.6 | 1 | 1 | 1 |
| E045 BLD.CD4.CD25I.CD127.TMEMPC (Primary T cells effector/memory enriched from peripheral blood) | 1 | 0.3 | 0.5 | 0.226581 | 0.378863 |
| E033 BLD.CD3.CPC (Primary T cells from cord blood) | 1 | 0.4 | 0.7 | 0.345526 | 0.500807 |
| E044 BLD.CD4.CD25.CD127M.TREGPC (Primary T regulatory cells from peripheral blood) | 0 | 0.3 | 0.6 | 1 | 1 |
| E043 BLD.CD4.CD25M.TPC (Primary T helper cells from peripheral blood) | 2 | 0.6 | 0.9 | 0.110738 | 0.241136 |
| E039 BLD.CD4.CD25M.CD45RA.NPC (Primary T helper naive cells from peripheral blood) | 2 | 0.5 | 0.8 | 0.092062 | 0.191862 |
| E041 BLD.CD4.CD25M.IL17M.PL.TPC (Primary T helper cells PMA-I stimulated) | 2 | 0.6 | 0.9 | 0.119824 | 0.238762 |
| E042 BLD.CD4.CD25M.IL17P.PL.TPC (Primary T helper 17 cells PMA-I stimulated) | 2 | 0.5 | 0.8 | 0.07436 | 0.203456 |
| E040 BLD.CD4.CD25M.CD45RO.MPC (Primary T helper memory cells from peripheral blood 1) | 1 | 0.5 | 0.8 | 0.390292 | 0.573902 |
| E037 BLD.CD4.MPC (Primary T helper memory cells from peripheral blood 2) | 2 | 0.5 | 0.9 | 0.102269 | 0.231651 |
| E048 BLD.CD8.MPC (Primary T CD8+ memory cells from peripheral blood) | 2 | 0.4 | 0.8 | 0.066002 | 0.17129 |
| E038 BLD.CD4.NPC (Primary T helper naive cells from peripheral blood) | 2 | 0.5 | 0.7 | 0.074131 | 0.169031 |
| E047 BLD.CD8.NPC (Primary T CD8+ naive cells from peripheral blood) | 1 | 0.5 | 0.9 | 0.403483 | 0.609163 |
| E029 BLD.CD14.PC (Primary monocytes from peripheral blood) | 1 | 0.7 | 1.3 | 0.529721 | 0.742716 |
| E031 BLD.CD19.CPC (Primary B cells from cord blood) | 1 | 0.5 | 0.8 | 0.370388 | 0.555169 |
| E035 BLD.CD34.PC (Primary hematopoietic stem cells) | 1 | 0.5 | 0.9 | 0.395236 | 0.609163 |
| E051 BLD.MOB.CD34.PC.M (Primary hematopoietic stem cells G-CSF-mobilized Male) | 1 | 0.7 | 1.2 | 0.498105 | 0.699187 |
| E050 BLD.MOB.CD34.PC.F (Primary hematopoietic stem cells G-CSF-mobilized Female) | 1 | 0.7 | 1.3 | 0.512941 | 0.72737 |
| E036 BLD.CD34.CC (Primary hematopoietic stem cells short term culture) | 1 | 0.6 | 1.2 | 0.482274 | 0.707224 |
| E032 BLD.CD19.PPC (Primary B cells from peripheral blood) | 0 | 0.7 | 1.1 | 1 | 1 |
| E046 BLD.CD56.PC (Primary Natural Killer cells from peripheral blood) | 0 | 0.6 | 1 | 1 | 1 |
| E030 BLD.CD15.PC (Primary neutrophils from peripheral blood) | 2 | 0.5 | 0.8 | 0.088382 | 0.202292 |
| E026 STRM.MRW.MSC (Bone Marrow Derived Cultured Mesenchymal Stem Cells) | 2 | 0.6 | 1.1 | 0.118766 | 0.288899 |
| E049 STRM.CHON.MRW.DR.MSC (Mesenchymal Stem Cell Derived Chondrocyte Cultured Cells) | 1 | 0.7 | 1.3 | 0.514979 | 0.749653 |
| E025 FAT.ADIP.DR.MSC (Adipose Derived Mesenchymal Stem Cell Cultured Cells) | 3 | 1 | 1.6 | 0.06907 | 0.215628 |
| E023 FAT.MSC.DR.ADIP (Mesenchymal Stem Cell Derived Adipocyte Cultured Cells) | 1 | 0.7 | 1.2 | 0.523099 | 0.717945 |
| E052 MUS.SAT (Muscle Satellite Cultured Cells) | 2 | 0.7 | 1.2 | 0.143982 | 0.331903 |
| E055 SKIN.PEN.FRSK.FIB.01 (Foreskin Fibroblast Primary Cells skin01) | 2 | 0.7 | 1.3 | 0.163783 | 0.387269 |
| E056 SKIN.PEN.FRSK.FIB.02 (Foreskin Fibroblast Primary Cells skin02) | 1 | 0.5 | 1 | 0.400246 | 0.631946 |
| E059 SKIN.PEN.FRSK.MEL.01 (Foreskin Melanocyte Primary Cells skin01) | 0 | 0.4 | 0.8 | 1 | 1 |
| E061 SKIN.PEN.FRSK.MEL.03 (Foreskin Melanocyte Primary Cells skin03) | 0 | 0.7 | 1.1 | 1 | 1 |
| E057 SKIN.PEN.FRSK.KER.02 (Foreskin Keratinocyte Primary Cells skin02) | 0 | 0.7 | 1.2 | 1 | 1 |
| E058 SKIN.PEN.FRSK.KER.03 (Foreskin Keratinocyte Primary Cells skin03) | 0 | 0.7 | 1.2 | 1 | 1 |
| E028 BRST.HMEC.35 (Breast variant Human Mammary Epithelial Cells (vHMEC)) | 1 | 0.7 | 1.3 | 0.525569 | 0.740954 |
| E027 BRST.MYO (Breast Myoepithelial Primary Cells) | 2 | 0.9 | 1.6 | 0.211953 | 0.495707 |
| E054 BRN.GANGEM.DR.NRSPHR (Ganglion Eminence derived primary cultured neurospheres) | 0 | 0.5 | 0.7 | 1 | 1 |
| E053 BRN.CRTX.DR.NRSPHR (Cortex derived primary cultured neurospheres) | 1 | 0.7 | 1 | 0.485579 | 0.645246 |
| E112 THYM (Thymus) | 0 | 0.3 | 0.6 | 1 | 1 |
| E093 THYM.FET (Fetal Thymus) | 1 | 0.6 | 1.2 | 0.469322 | 0.707224 |
| E071 BRN.HIPP.MID (Brain Hippocampus Middle) | 0 | 0.6 | 1 | 1 | 1 |
| E074 BRN.SUB.NIG (Brain Substantia Nigra) | 0 | 0.5 | 1 | 1 | 1 |
| E068 BRN.ANT.CAUD (Brain Anterior Caudate) | 0 | 0.5 | 0.9 | 1 | 1 |
| E069 BRN.CING.GYR (Brain Cingulate Gyrus) | 0 | 0.5 | 0.9 | 1 | 1 |
| E072 BRN.INF.TMP (Brain Inferior Temporal Lobe) | 0 | 0.5 | 0.8 | 1 | 1 |
| E067 BRN.ANG.GYR (Brain Angular Gyrus) | 0 | 0.4 | 0.7 | 1 | 1 |
| E073 BRN.DL.PRFRNTL.CRTX (Brain Dorsolateral Prefrontal Cortex) | 0 | 0.4 | 0.7 | 1 | 1 |
| E070 BRN.GRM.MTRX (Brain Germinal Matrix) | 0 | 0.4 | 0.6 | 1 | 1 |
| E082 BRN.FET.F (Fetal Brain Female) | 2 | 0.3 | 0.5 | 0.034739 | 0.081655 |
| E081 BRN.FET.M (Fetal Brain Male) | 0 | 0.6 | 0.8 | 1 | 1 |
| E063 FAT.ADIP.NUC (Adipose Nuclei) | 0 | 0.7 | 1.3 | 1 | 1 |
| E100 MUS.PSOAS (Psoas Muscle) | 1 | 0.4 | 0.6 | 0.305561 | 0.472201 |
| E108 MUS.SKLT.F (Skeletal Muscle Female) | 0 | 0.7 | 1.2 | 1 | 1 |
| E107 MUS.SKLT.M (Skeletal Muscle Male) | 2 | 0.7 | 1.2 | 0.14362 | 0.349705 |
| E089 MUS.TRNK.FET (Fetal Muscle Trunk) | 0 | 0.7 | 1.3 | 1 | 1 |
| E090 MUS.LEG.FET (Fetal Muscle Leg) | 1 | 1 | 1.8 | 0.624097 | 0.849697 |
| E083 HRT.FET (Fetal Heart) | 2 | 0.9 | 1.5 | 0.216803 | 0.437637 |
| E104 HRT.ATR.R (Right Atrium) | 2 | 0.4 | 0.8 | 0.067713 | 0.197646 |
| E095 HRT.VENT.L (Left Ventricle) | 2 | 0.6 | 1.1 | 0.113537 | 0.291293 |
| E105 HRT.VNT.R (Right Ventricle) | 1 | 0.5 | 0.9 | 0.372805 | 0.593245 |
| E065 VAS.AOR (Aorta) | 0 | 0.2 | 0.4 | 1 | 1 |
| E078 GI.DUO.SM.MUS (Duodenum Smooth Muscle) | 0 | 0.3 | 0.6 | 1 | 1 |
| E076 GI.CLN.SM.MUS (Colon Smooth Muscle) | 0 | 0.5 | 1 | 1 | 1 |
| E103 GI.RECT.SM.MUS (Rectal Smooth Muscle) | 0 | 0.4 | 0.7 | 1 | 1 |
| E111 GI.STMC.MUS (Stomach Smooth Muscle) | 1 | 0.4 | 0.7 | 0.324818 | 0.502443 |
| E092 GI.STMC.FET (Fetal Stomach) | 0 | 0.6 | 1.2 | 1 | 1 |
| E085 GI.S.INT.FET (Fetal Intestine Small) | 1 | 0.6 | 1.1 | 0.482543 | 0.693024 |
| E084 GI.L.INT.FET (Fetal Intestine Large) | 1 | 0.6 | 1.1 | 0.467263 | 0.686742 |
| E109 GI.S.INT (Small Intestine) | 0 | 0.2 | 0.4 | 1 | 1 |
| E106 GI.CLN.SIG (Sigmoid Colon) | 0 | 0.3 | 0.7 | 1 | 1 |
| E075 GI.CLN.MUC (Colonic Mucosa) | 0 | 0.2 | 0.4 | 1 | 1 |
| E101 GI.RECT.MUC.29 (Rectal Mucosa Donor 29) | 0 | 0.3 | 0.6 | 1 | 1 |
| E102 GI.RECT.MUC.31 (Rectal Mucosa Donor 31) | 0 | 0.4 | 0.8 | 1 | 1 |
| E110 GI.STMC.MUC (Stomach Mucosa) | 0 | 0.6 | 1 | 1 | 1 |
| E077 GI.DUO.MUC (Duodenum Mucosa) | 0 | 0.4 | 0.9 | 1 | 1 |
| E079 GI.ESO (Esophagus) | 0 | 0.4 | 0.7 | 1 | 1 |
| E094 GI.STMC.GAST (Gastric) | 1 | 0.3 | 0.6 | 0.275172 | 0.472201 |
| E099 PLCNT.AMN (Placenta Amnion) | 0 | 0.4 | 0.8 | 1 | 1 |
| E086 KID.FET (Fetal Kidney) | 0 | 0.3 | 0.6 | 1 | 1 |
| E088 LNG.FET (Fetal Lung) | 0 | 0.7 | 1.3 | 1 | 1 |
| E097 OVRY (Ovary) | 0 | 0.4 | 0.8 | 1 | 1 |
| E087 PANC.ISLT (Pancreatic Islets) | 0 | 0.2 | 0.3 | 1 | 1 |
| E080 ADRL.GLND.FET (Fetal Adrenal Gland) | 1 | 0.8 | 1.5 | 0.570628 | 0.800528 |
| E091 PLCNT.FET (Placenta) | 0 | 0.8 | 1.5 | 1 | 1 |
| E066 LIV.ADLT (Liver) | 2 | 0.7 | 1.2 | 0.138617 | 0.329523 |
| E098 PANC (Pancreas) | 1 | 0.5 | 0.9 | 0.416683 | 0.597281 |
| E096 LNG (Lung) | 0 | 0.4 | 0.8 | 1 | 1 |
| E113 SPLN (Spleen) | 0 | 0.5 | 1.1 | 1 | 1 |
| E114 LNG.A549.ETOH002.CNCR (A549 EtOH 0.02pct Lung Carcinoma Cell Line) | 3 | 0.5 | 0.9 | 0.014163 | 0.065727 |
| E115 BLD.DND41.CNCR (Dnd41 TCell Leukemia Cell Line) | 2 | 0.4 | 0.5 | 0.059701 | 0.099829 |
| E116 BLD.GM12878 (GM12878 Lymphoblastoid Cells) | 0 | 0.5 | 0.8 | 1 | 1 |
| E117 CRVX.HELAS3.CNCR (HeLa-S3 Cervical Carcinoma Cell Line) | 0 | 0.5 | 0.9 | 1 | 1 |
| E118 LIV.HEPG2.CNCR (HepG2 Hepatocellular Carcinoma Cell Line) | 2 | 0.8 | 1.4 | 0.205863 | 0.413811 |
| E119 BRST.HMEC (HMEC Mammary Epithelial Primary Cells) | 0 | 0.7 | 1.2 | 1 | 1 |
| E120 MUS.HSMM (HSMM Skeletal Muscle Myoblasts Cells) | 2 | 0.5 | 1.1 | 0.100777 | 0.298474 |
| E121 MUS.HSMMT (HSMM cell derived Skeletal Muscle Myotubes Cells) | 0 | 0.5 | 1 | 1 | 1 |
| E122 VAS.HUVEC (HUVEC Umbilical Vein Endothelial Primary Cells) | 0 | 0.6 | 0.9 | 1 | 1 |
| E123 BLD.K562.CNCR (K562 Leukemia Cells) | 0 | 0.5 | 0.8 | 1 | 1 |
| E124 BLD.CD14.MONO (Monocytes-CD14+ RO01746 Primary Cells) | 0 | 0.5 | 0.8 | 1 | 1 |
| E125 BRN.NHA (NH-A Astrocytes Primary Cells) | 1 | 0.5 | 1 | 0.406625 | 0.629477 |
| E126 SKIN.NHDFAD (NHDF-Ad Adult Dermal Fibroblast Primary Cells) | 0 | 0.7 | 1.2 | 1 | 1 |
| E127 SKIN.NHEK (NHEK-Epidermal Keratinocyte Primary Cells) | 0 | 0.7 | 1.1 | 1 | 1 |
| E128 LNG.NHLF (NHLF Lung Fibroblast Primary Cells) | 1 | 0.5 | 0.9 | 0.385113 | 0.610463 |
| E129 BONE.OSTEO (Osteoblast Primary Cells) | 1 | 0.6 | 1.1 | 0.479434 | 0.680339 |

**Supplementary Table S2-6**. Query SNP enhancer summary for PTA

| Cell | Observed | Expected | Expected | Binomial *P* | Binomial *P* |
| --- | --- | --- | --- | --- | --- |
|  |  | (all SNPs) | (GWAS SNPs) | (all SNPs) | (GWAS SNPs) |
| E017 LNG.IMR90 (IMR90 fetal lung fibroblasts Cell Line) | 1 | 0.2 | 0.4 | 0.200859 | 0.34719 |
| E002 ESC.WA7 (ES-WA7 Cells) | 0 | 0.1 | 0.1 | 1 | 1 |
| E008 ESC.H9 (H9 Cells) | 0 | 0.1 | 0.2 | 1 | 1 |
| E001 ESC.I3 (ES-I3 Cells) | 0 | 0.2 | 0.3 | 1 | 1 |
| E015 ESC.HUES6 (HUES6 Cells) | 0 | 0.2 | 0.3 | 1 | 1 |
| E014 ESC.HUES48 (HUES48 Cells) | 0 | 0.2 | 0.3 | 1 | 1 |
| E016 ESC.HUES64 (HUES64 Cells) | 0 | 0.2 | 0.3 | 1 | 1 |
| E003 ESC.H1 (H1 Cells) | 0 | 0.2 | 0.3 | 1 | 1 |
| E024 ESC.4STAR (ES-UCSF4 Cells) | 0 | 0.2 | 0.3 | 1 | 1 |
| E020 IPSC.20B (iPS-20b Cells) | 0 | 0.1 | 0.2 | 1 | 1 |
| E019 IPSC.18 (iPS-18 Cells) | 0 | 0.2 | 0.3 | 1 | 1 |
| E018 IPSC.15b (iPS-15b Cells) | 0 | 0.2 | 0.3 | 1 | 1 |
| E021 IPSC.DF.6.9 (iPS DF 6.9 Cells) | 0 | 0.1 | 0.2 | 1 | 1 |
| E022 IPSC.DF.19.11 (iPS DF 19.11 Cells) | 0 | 0.2 | 0.4 | 1 | 1 |
| E007 ESDR.H1.NEUR.PROG (H1 Derived Neuronal Progenitor Cultured Cells) | 0 | 0.1 | 0.2 | 1 | 1 |
| E009 ESDR.H9.NEUR.PROG (H9 Derived Neuronal Progenitor Cultured Cells) | 0 | 0.1 | 0.2 | 1 | 1 |
| E010 ESDR.H9.NEUR (H9 Derived Neuron Cultured Cells) | 0 | 0.2 | 0.3 | 1 | 1 |
| E013 ESDR.CD56.MESO (hESC Derived CD56+ Mesoderm Cultured Cells) | 0 | 0.2 | 0.3 | 1 | 1 |
| E012 ESDR.CD56.ECTO (hESC Derived CD56+ Ectoderm Cultured Cells) | 0 | 0.1 | 0.3 | 1 | 1 |
| E011 ESDR.CD184.ENDO (hESC Derived CD184+ Endoderm Cultured Cells) | 0 | 0.2 | 0.3 | 1 | 1 |
| E004 ESDR.H1.BMP4.MESO (H1 BMP4 Derived Mesendoderm Cultured Cells) | 0 | 0.1 | 0.2 | 1 | 1 |
| E005 ESDR.H1.BMP4.TROP (H1 BMP4 Derived Trophoblast Cultured Cells) | 0 | 0.2 | 0.4 | 1 | 1 |
| E006 ESDR.H1.MSC (H1 Derived Mesenchymal Stem Cells) | 0 | 0.2 | 0.4 | 1 | 1 |
| E062 BLD.PER.MONUC.PC (Primary mononuclear cells from peripheral blood) | 0 | 0.1 | 0.1 | 1 | 1 |
| E034 BLD.CD3.PPC (Primary T cells from peripheral blood) | 0 | 0.2 | 0.3 | 1 | 1 |
| E045 BLD.CD4.CD25I.CD127.TMEMPC (Primary T cells effector/memory enriched from peripheral blood) | 0 | 0.1 | 0.1 | 1 | 1 |
| E033 BLD.CD3.CPC (Primary T cells from cord blood) | 0 | 0.1 | 0.2 | 1 | 1 |
| E044 BLD.CD4.CD25.CD127M.TREGPC (Primary T regulatory cells from peripheral blood) | 0 | 0.1 | 0.2 | 1 | 1 |
| E043 BLD.CD4.CD25M.TPC (Primary T helper cells from peripheral blood) | 0 | 0.2 | 0.3 | 1 | 1 |
| E039 BLD.CD4.CD25M.CD45RA.NPC (Primary T helper naive cells from peripheral blood) | 0 | 0.2 | 0.2 | 1 | 1 |
| E041 BLD.CD4.CD25M.IL17M.PL.TPC (Primary T helper cells PMA-I stimulated) | 0 | 0.2 | 0.3 | 1 | 1 |
| E042 BLD.CD4.CD25M.IL17P.PL.TPC (Primary T helper 17 cells PMA-I stimulated) | 0 | 0.1 | 0.2 | 1 | 1 |
| E040 BLD.CD4.CD25M.CD45RO.MPC (Primary T helper memory cells from peripheral blood 1) | 0 | 0.1 | 0.2 | 1 | 1 |
| E037 BLD.CD4.MPC (Primary T helper memory cells from peripheral blood 2) | 0 | 0.2 | 0.3 | 1 | 1 |
| E048 BLD.CD8.MPC (Primary T CD8+ memory cells from peripheral blood) | 0 | 0.1 | 0.2 | 1 | 1 |
| E038 BLD.CD4.NPC (Primary T helper naive cells from peripheral blood) | 0 | 0.1 | 0.2 | 1 | 1 |
| E047 BLD.CD8.NPC (Primary T CD8+ naive cells from peripheral blood) | 0 | 0.1 | 0.3 | 1 | 1 |
| E029 BLD.CD14.PC (Primary monocytes from peripheral blood) | 1 | 0.2 | 0.4 | 0.198997 | 0.329202 |
| E031 BLD.CD19.CPC (Primary B cells from cord blood) | 1 | 0.1 | 0.2 | 0.127222 | 0.211997 |
| E035 BLD.CD34.PC (Primary hematopoietic stem cells) | 0 | 0.1 | 0.3 | 1 | 1 |
| E051 BLD.MOB.CD34.PC.M (Primary hematopoietic stem cells G-CSF-mobilized Male) | 0 | 0.2 | 0.3 | 1 | 1 |
| E050 BLD.MOB.CD34.PC.F (Primary hematopoietic stem cells G-CSF-mobilized Female) | 0 | 0.2 | 0.4 | 1 | 1 |
| E036 BLD.CD34.CC (Primary hematopoietic stem cells short term culture) | 0 | 0.2 | 0.3 | 1 | 1 |
| E032 BLD.CD19.PPC (Primary B cells from peripheral blood) | 0 | 0.2 | 0.3 | 1 | 1 |
| E046 BLD.CD56.PC (Primary Natural Killer cells from peripheral blood) | 0 | 0.2 | 0.3 | 1 | 1 |
| E030 BLD.CD15.PC (Primary neutrophils from peripheral blood) | 1 | 0.1 | 0.2 | 0.139094 | 0.222664 |
| E026 STRM.MRW.MSC (Bone Marrow Derived Cultured Mesenchymal Stem Cells) | 0 | 0.2 | 0.3 | 1 | 1 |
| E049 STRM.CHON.MRW.DR.MSC (Mesenchymal Stem Cell Derived Chondrocyte Cultured Cells) | 2 | 0.2 | 0.4 | 0.015961 | 0.05218 |
| E025 FAT.ADIP.DR.MSC (Adipose Derived Mesenchymal Stem Cell Cultured Cells) | 0 | 0.3 | 0.5 | 1 | 1 |
| E023 FAT.MSC.DR.ADIP (Mesenchymal Stem Cell Derived Adipocyte Cultured Cells) | 1 | 0.2 | 0.4 | 0.195696 | 0.310819 |
| E052 MUS.SAT (Muscle Satellite Cultured Cells) | 1 | 0.2 | 0.3 | 0.182781 | 0.301826 |
| E055 SKIN.PEN.FRSK.FIB.01 (Foreskin Fibroblast Primary Cells skin01) | 0 | 0.2 | 0.4 | 1 | 1 |
| E056 SKIN.PEN.FRSK.FIB.02 (Foreskin Fibroblast Primary Cells skin02) | 0 | 0.1 | 0.3 | 1 | 1 |
| E059 SKIN.PEN.FRSK.MEL.01 (Foreskin Melanocyte Primary Cells skin01) | 0 | 0.1 | 0.2 | 1 | 1 |
| E061 SKIN.PEN.FRSK.MEL.03 (Foreskin Melanocyte Primary Cells skin03) | 1 | 0.2 | 0.3 | 0.197279 | 0.294844 |
| E057 SKIN.PEN.FRSK.KER.02 (Foreskin Keratinocyte Primary Cells skin02) | 0 | 0.2 | 0.3 | 1 | 1 |
| E058 SKIN.PEN.FRSK.KER.03 (Foreskin Keratinocyte Primary Cells skin03) | 0 | 0.2 | 0.3 | 1 | 1 |
| E028 BRST.HMEC.35 (Breast variant Human Mammary Epithelial Cells (vHMEC)) | 0 | 0.2 | 0.4 | 1 | 1 |
| E027 BRST.MYO (Breast Myoepithelial Primary Cells) | 0 | 0.3 | 0.5 | 1 | 1 |
| E054 BRN.GANGEM.DR.NRSPHR (Ganglion Eminence derived primary cultured neurospheres) | 1 | 0.1 | 0.2 | 0.127755 | 0.194998 |
| E053 BRN.CRTX.DR.NRSPHR (Cortex derived primary cultured neurospheres) | 0 | 0.2 | 0.3 | 1 | 1 |
| E112 THYM (Thymus) | 0 | 0.1 | 0.2 | 1 | 1 |
| E093 THYM.FET (Fetal Thymus) | 0 | 0.2 | 0.3 | 1 | 1 |
| E071 BRN.HIPP.MID (Brain Hippocampus Middle) | 0 | 0.2 | 0.3 | 1 | 1 |
| E074 BRN.SUB.NIG (Brain Substantia Nigra) | 0 | 0.2 | 0.3 | 1 | 1 |
| E068 BRN.ANT.CAUD (Brain Anterior Caudate) | 0 | 0.1 | 0.3 | 1 | 1 |
| E069 BRN.CING.GYR (Brain Cingulate Gyrus) | 0 | 0.1 | 0.3 | 1 | 1 |
| E072 BRN.INF.TMP (Brain Inferior Temporal Lobe) | 1 | 0.1 | 0.2 | 0.129684 | 0.211997 |
| E067 BRN.ANG.GYR (Brain Angular Gyrus) | 1 | 0.1 | 0.2 | 0.114238 | 0.193438 |
| E073 BRN.DL.PRFRNTL.CRTX (Brain Dorsolateral Prefrontal Cortex) | 0 | 0.1 | 0.2 | 1 | 1 |
| E070 BRN.GRM.MTRX (Brain Germinal Matrix) | 0 | 0.1 | 0.2 | 1 | 1 |
| E082 BRN.FET.F (Fetal Brain Female) | 0 | 0.1 | 0.1 | 1 | 1 |
| E081 BRN.FET.M (Fetal Brain Male) | 0 | 0.2 | 0.2 | 1 | 1 |
| E063 FAT.ADIP.NUC (Adipose Nuclei) | 0 | 0.2 | 0.4 | 1 | 1 |
| E100 MUS.PSOAS (Psoas Muscle) | 0 | 0.1 | 0.2 | 1 | 1 |
| E108 MUS.SKLT.F (Skeletal Muscle Female) | 0 | 0.2 | 0.4 | 1 | 1 |
| E107 MUS.SKLT.M (Skeletal Muscle Male) | 1 | 0.2 | 0.4 | 0.182518 | 0.312194 |
| E089 MUS.TRNK.FET (Fetal Muscle Trunk) | 0 | 0.2 | 0.4 | 1 | 1 |
| E090 MUS.LEG.FET (Fetal Muscle Leg) | 0 | 0.3 | 0.5 | 1 | 1 |
| E083 HRT.FET (Fetal Heart) | 0 | 0.3 | 0.4 | 1 | 1 |
| E104 HRT.ATR.R (Right Atrium) | 1 | 0.1 | 0.2 | 0.12026 | 0.219628 |
| E095 HRT.VENT.L (Left Ventricle) | 1 | 0.2 | 0.3 | 0.159827 | 0.27786 |
| E105 HRT.VNT.R (Right Ventricle) | 1 | 0.1 | 0.3 | 0.128209 | 0.232465 |
| E065 VAS.AOR (Aorta) | 0 | 0.1 | 0.1 | 1 | 1 |
| E078 GI.DUO.SM.MUS (Duodenum Smooth Muscle) | 0 | 0.1 | 0.2 | 1 | 1 |
| E076 GI.CLN.SM.MUS (Colon Smooth Muscle) | 0 | 0.2 | 0.3 | 1 | 1 |
| E103 GI.RECT.SM.MUS (Rectal Smooth Muscle) | 0 | 0.1 | 0.2 | 1 | 1 |
| E111 GI.STMC.MUS (Stomach Smooth Muscle) | 0 | 0.1 | 0.2 | 1 | 1 |
| E092 GI.STMC.FET (Fetal Stomach) | 0 | 0.2 | 0.3 | 1 | 1 |
| E085 GI.S.INT.FET (Fetal Intestine Small) | 1 | 0.2 | 0.3 | 0.176155 | 0.293441 |
| E084 GI.L.INT.FET (Fetal Intestine Large) | 2 | 0.2 | 0.3 | 0.012288 | 0.038089 |
| E109 GI.S.INT (Small Intestine) | 0 | 0.1 | 0.1 | 1 | 1 |
| E106 GI.CLN.SIG (Sigmoid Colon) | 0 | 0.1 | 0.2 | 1 | 1 |
| E075 GI.CLN.MUC (Colonic Mucosa) | 0 | 0.1 | 0.1 | 1 | 1 |
| E101 GI.RECT.MUC.29 (Rectal Mucosa Donor 29) | 1 | 0.1 | 0.2 | 0.079053 | 0.152027 |
| E102 GI.RECT.MUC.31 (Rectal Mucosa Donor 31) | 0 | 0.1 | 0.2 | 1 | 1 |
| E110 GI.STMC.MUC (Stomach Mucosa) | 0 | 0.2 | 0.3 | 1 | 1 |
| E077 GI.DUO.MUC (Duodenum Mucosa) | 0 | 0.1 | 0.3 | 1 | 1 |
| E079 GI.ESO (Esophagus) | 0 | 0.1 | 0.2 | 1 | 1 |
| E094 GI.STMC.GAST (Gastric) | 0 | 0.1 | 0.2 | 1 | 1 |
| E099 PLCNT.AMN (Placenta Amnion) | 0 | 0.1 | 0.2 | 1 | 1 |
| E086 KID.FET (Fetal Kidney) | 0 | 0.1 | 0.2 | 1 | 1 |
| E088 LNG.FET (Fetal Lung) | 1 | 0.2 | 0.4 | 0.184545 | 0.317674 |
| E097 OVRY (Ovary) | 1 | 0.1 | 0.2 | 0.125431 | 0.21582 |
| E087 PANC.ISLT (Pancreatic Islets) | 0 | 0.1 | 0.1 | 1 | 1 |
| E080 ADRL.GLND.FET (Fetal Adrenal Gland) | 0 | 0.2 | 0.5 | 1 | 1 |
| E091 PLCNT.FET (Placenta) | 1 | 0.2 | 0.5 | 0.208938 | 0.376946 |
| E066 LIV.ADLT (Liver) | 0 | 0.2 | 0.3 | 1 | 1 |
| E098 PANC (Pancreas) | 1 | 0.2 | 0.3 | 0.146608 | 0.234713 |
| E096 LNG (Lung) | 0 | 0.1 | 0.2 | 1 | 1 |
| E113 SPLN (Spleen) | 0 | 0.2 | 0.3 | 1 | 1 |
| E114 LNG.A549.ETOH002.CNCR (A549 EtOH 0.02pct Lung Carcinoma Cell Line) | 1 | 0.2 | 0.3 | 0.144055 | 0.249564 |
| E115 BLD.DND41.CNCR (Dnd41 TCell Leukemia Cell Line) | 0 | 0.1 | 0.2 | 1 | 1 |
| E116 BLD.GM12878 (GM12878 Lymphoblastoid Cells) | 0 | 0.1 | 0.2 | 1 | 1 |
| E117 CRVX.HELAS3.CNCR (HeLa-S3 Cervical Carcinoma Cell Line) | 0 | 0.1 | 0.3 | 1 | 1 |
| E118 LIV.HEPG2.CNCR (HepG2 Hepatocellular Carcinoma Cell Line) | 1 | 0.2 | 0.4 | 0.224984 | 0.349164 |
| E119 BRST.HMEC (HMEC Mammary Epithelial Primary Cells) | 0 | 0.2 | 0.4 | 1 | 1 |
| E120 MUS.HSMM (HSMM Skeletal Muscle Myoblasts Cells) | 1 | 0.2 | 0.3 | 0.149555 | 0.282136 |
| E121 MUS.HSMMT (HSMM cell derived Skeletal Muscle Myotubes Cells) | 0 | 0.2 | 0.3 | 1 | 1 |
| E122 VAS.HUVEC (HUVEC Umbilical Vein Endothelial Primary Cells) | 0 | 0.2 | 0.3 | 1 | 1 |
| E123 BLD.K562.CNCR (K562 Leukemia Cells) | 1 | 0.2 | 0.2 | 0.146901 | 0.222664 |
| E124 BLD.CD14.MONO (Monocytes-CD14+ RO01746 Primary Cells) | 1 | 0.1 | 0.2 | 0.137716 | 0.222664 |
| E125 BRN.NHA (NH-A Astrocytes Primary Cells) | 0 | 0.2 | 0.3 | 1 | 1 |
| E126 SKIN.NHDFAD (NHDF-Ad Adult Dermal Fibroblast Primary Cells) | 0 | 0.2 | 0.4 | 1 | 1 |
| E127 SKIN.NHEK (NHEK-Epidermal Keratinocyte Primary Cells) | 0 | 0.2 | 0.3 | 1 | 1 |
| E128 LNG.NHLF (NHLF Lung Fibroblast Primary Cells) | 1 | 0.1 | 0.3 | 0.133276 | 0.242168 |
| E129 BONE.OSTEO (Osteoblast Primary Cells) | 0 | 0.2 | 0.3 | 1 | 1 |
