# Supplementary material for: A Genome-Wide Association Study of Age-Related Hearing Impairment in Middle- and Old-Aged Chinese Twins
Source: Biomed Res Int. 2021 Jul 17;2021:3629624. doi: 10.1155/2021/3629624 (PMC8314043; doi:10.1155/2021/3629624)
Supplement: Supplementary 3 — Additional file 3: the genes nominally associated with BEHLs at each frequency and PTA from VEGAS2 gene-based analysis (P < 0.05). [file 3629624.f3.docx]

**Supplementary Table S3-1**. The genes nominally associated with BEHLs at the frequency of 0.5 kHz from VEGAS2 gene-based analysis (*P* < 0.05)

| Chr | Gene | nSNPs | Start position | Stop position | Gene-based test statistic | P-value | Top-SNP | Top-SNP P-value |
| --- | --- | --- | --- | --- | --- | --- | --- | --- |
| 17 | *RNASEK-C17orf49* | 6 | 6915735 | 6920843 | 70.85 | 2.00E-05 | rs7338 | 8.40E-05 |
| 17 | *C17orf49* | 5 | 6918055 | 6920843 | 55.38 | 2.20E-05 | rs14309 | 1.70E-04 |
| 20 | *C20orf196* | 72 | 5731042 | 5844559 | 273.82 | 6.00E-05 | rs237076 | 6.20E-07 |
| 17 | *MIR497HG* | 3 | 6919136 | 6922973 | 30.88 | 1.50E-04 | rs11078662 | 2.20E-04 |
| 19 | *MYO1F* | 26 | 8585673 | 8642331 | 115.33 | 2.80E-04 | rs3213834 | 2.60E-05 |
| 16 | *ITGAM* | 11 | 31271287 | 31344213 | 135.61 | 3.70E-04 | rs4594268 | 9.00E-05 |
| 3 | *SLC7A14* | 64 | 170177341 | 170303863 | 263.01 | 5.10E-04 | rs6790988 | 1.80E-06 |
| 12 | *CAPS2* | 28 | 75669758 | 75784702 | 157.74 | 5.20E-04 | rs12367329 | 8.10E-05 |
| 7 | *SAMD9* | 14 | 92728825 | 92747336 | 75.2 | 5.60E-04 | rs76427362 | 4.80E-03 |
| 4 | *MGARP* | 7 | 140187316 | 140201492 | 37.55 | 5.80E-04 | rs13120574 | 1.20E-03 |
| 12 | *GLIPR1L1* | 8 | 75728462 | 75764169 | 48.74 | 6.10E-04 | rs12367329 | 8.10E-05 |
| 22 | *MLC1* | 24 | 50497819 | 50524358 | 132.45 | 6.10E-04 | rs5771144 | 1.10E-04 |
| 18 | *RBFA* | 7 | 77794345 | 77810652 | 40.58 | 6.20E-04 | rs3744873 | 1.60E-03 |
| 23 | *GPR50* | 2 | 150345055 | 150349937 | 14.76 | 6.50E-04 | rs2072621 | 3.30E-03 |
| 14 | *GPR132* | 8 | 105515725 | 105531887 | 34.35 | 6.70E-04 | rs7147439 | 3.40E-03 |
| 15 | *STARD9* | 66 | 42867856 | 43013196 | 253.11 | 6.80E-04 | rs61192504 | 6.40E-04 |
| 11 | *LGR4* | 38 | 27387507 | 27494334 | 173.89 | 7.20E-04 | rs11029994 | 9.10E-04 |
| 12 | *GLIPR1* | 7 | 75874512 | 75895716 | 44.38 | 7.50E-04 | rs11180546 | 1.40E-04 |
| 18 | *FLJ44087* | 44 | 43018148 | 43087001 | 211.76 | 8.40E-04 | rs72912678 | 5.30E-05 |
| 18 | *LINC-ROR* | 8 | 54721803 | 54739350 | 45.04 | 8.60E-04 | rs1942348 | 8.10E-04 |
| 19 | *PIK3R2* | 4 | 18263987 | 18281343 | 28 | 8.80E-04 | rs2267922 | 4.90E-04 |
| 23 | *CT55* | 2 | 134290460 | 134305751 | 13.95 | 9.50E-04 | rs7062590 | 6.10E-03 |
| 1 | *FNDC5* | 2 | 33327868 | 33338093 | 20.71 | 9.60E-04 | rs1746661 | 7.30E-04 |
| 6 | *CAGE1* | 27 | 7326886 | 7389942 | 134.08 | 9.80E-04 | rs11243161 | 8.00E-05 |
| 4 | *CCRN4L* | 9 | 139936912 | 139967093 | 48.09 | 9.80E-04 | rs10212985 | 2.70E-04 |
| 16 | *C16orf59* | 2 | 2510114 | 2514964 | 14.94 | 1.00E-03 | rs3810794 | 1.20E-04 |
| 12 | *GALNT9* | 96 | 132680916 | 132905905 | 266.91 | 1.10E-03 | rs28505774 | 6.80E-05 |
| 1 | *YARS* | 7 | 33240839 | 33283633 | 67.47 | 1.10E-03 | rs12564655 | 6.90E-04 |
| 6 | *TRMT11* | 18 | 126307575 | 126360420 | 165.99 | 1.10E-03 | rs9375421 | 2.80E-04 |
| 4 | *INPP4B* | 295 | 142949181 | 143767604 | 726.41 | 1.10E-03 | rs1908968 | 7.90E-06 |
| 15 | *SEMA7A* | 20 | 74701629 | 74726299 | 93.54 | 1.20E-03 | rs28362912 | 1.00E-04 |
| 16 | *MPV17L* | 8 | 15489610 | 15503543 | 37.71 | 1.20E-03 | rs153002 | 1.60E-04 |
| 9 | *LOC286367* | 3 | 107536632 | 107540045 | 21.63 | 1.20E-03 | rs2482430 | 8.10E-05 |
| 6 | *HINT3* | 9 | 126277860 | 126301389 | 93.38 | 1.20E-03 | rs9375419 | 5.10E-04 |
| 20 | *PRND* | 17 | 4702499 | 4709108 | 84.37 | 1.30E-03 | rs2756264 | 4.80E-04 |
| 5 | *LOC101929412* | 9 | 10493638 | 10502840 | 47.38 | 1.30E-03 | rs2567587 | 7.30E-04 |
| 16 | *ITGAX* | 16 | 31366454 | 31394318 | 90.4 | 1.40E-03 | rs13332545 | 3.60E-04 |
| 18 | *RNMT* | 10 | 13726703 | 13764554 | 60.2 | 1.40E-03 | rs6505838 | 4.80E-04 |
| 1 | *ALDH4A1* | 34 | 19197923 | 19229293 | 119.73 | 1.40E-03 | rs7366122 | 1.40E-05 |
| 16 | *C16orf45* | 32 | 15528324 | 15682116 | 142.7 | 1.60E-03 | rs222130 | 1.70E-04 |
| 15 | *VPS39* | 16 | 42450898 | 42500502 | 73.92 | 1.70E-03 | rs16972779 | 5.70E-04 |
| 7 | *PON1* | 65 | 94927668 | 94953884 | 268.94 | 1.70E-03 | rs854551 | 1.50E-04 |
| 18 | *SPIRE1* | 51 | 12446510 | 12657912 | 219.98 | 1.80E-03 | rs17615158 | 2.00E-04 |
| 12 | *GLIPR1L2* | 12 | 75784849 | 75826177 | 64.94 | 2.00E-03 | rs10785190 | 1.90E-04 |
| 17 | *SLC16A13* | 5 | 6939393 | 6943440 | 33.18 | 2.00E-03 | rs76070643 | 6.40E-04 |
| 1 | *S100PBP* | 6 | 33283042 | 33324480 | 35.07 | 2.00E-03 | rs9500 | 9.20E-04 |
| 16 | *EMP2* | 66 | 10622278 | 10674539 | 180.96 | 2.30E-03 | rs1504884 | 9.00E-04 |
| 19 | *MPND* | 10 | 4343523 | 4360083 | 36.3 | 2.40E-03 | rs56812200 | 2.40E-04 |
| 18 | *FAM210A* | 14 | 13663345 | 13726591 | 68.73 | 2.50E-03 | rs1284200 | 5.30E-04 |
| 5 | *LOC101929154* | 54 | 77180479 | 77254920 | 168.85 | 2.50E-03 | rs12656623 | 2.10E-05 |
| 15 | *ARID3B* | 12 | 74833547 | 74890472 | 62.19 | 2.80E-03 | rs10851873 | 7.60E-04 |
| 5 | *LINC00461* | 34 | 87836596 | 87980620 | 147.79 | 2.80E-03 | rs3814424 | 1.70E-04 |
| 19 | *GMFG* | 4 | 39818998 | 39826726 | 22.94 | 3.00E-03 | rs75976955 | 1.30E-04 |
| 19 | *ACER1* | 12 | 6306509 | 6333640 | 37.65 | 3.00E-03 | rs8104835 | 4.10E-04 |
| 3 | *CCDC66* | 28 | 56591183 | 56655848 | 104.74 | 3.00E-03 | rs955247 | 2.70E-04 |
| 18 | *LOC100505474* | 31 | 53750586 | 53804767 | 124.93 | 3.40E-03 | rs11151567 | 1.20E-04 |
| 3 | *ECE2* | 19 | 183967444 | 184010819 | 67.35 | 3.40E-03 | rs73187605 | 6.70E-04 |
| 15 | *LOC101928442* | 40 | 48095580 | 48138433 | 119.99 | 3.50E-03 | rs935875 | 1.60E-04 |
| 8 | *FAM183CP* | 8 | 29779028 | 29811123 | 39.82 | 3.50E-03 | rs10503858 | 5.20E-05 |
| 7 | *MRPS17* | 2 | 56019610 | 56023033 | 11.28 | 3.70E-03 | rs4307293 | 9.20E-04 |
| 19 | *LSR* | 8 | 35739558 | 35758867 | 38.76 | 3.80E-03 | rs2073900 | 5.80E-05 |
| 9 | *BRINP1* | 80 | 121928907 | 122131739 | 269.73 | 3.90E-03 | rs12238657 | 4.80E-04 |
| 9 | *LPPR1* | 162 | 103791030 | 104087417 | 401.17 | 4.30E-03 | rs57966478 | 1.70E-04 |
| 18 | *ATP8B1* | 111 | 55313658 | 55470327 | 279.83 | 4.40E-03 | rs9944837 | 2.50E-04 |
| 20 | *LOC100130264* | 27 | 19222945 | 19265240 | 90.42 | 4.40E-03 | rs6132186 | 5.10E-04 |
| 7 | *BLVRA* | 16 | 43798271 | 43846941 | 62.09 | 4.50E-03 | rs2074795 | 5.10E-04 |
| 18 | *LOC284294* | 185 | 61771324 | 62090827 | 415.44 | 4.70E-03 | rs7232351 | 4.30E-04 |
| 18 | *SLC14A2* | 306 | 42792946 | 43263060 | 654.58 | 4.80E-03 | rs72912678 | 5.30E-05 |
| 18 | *LOC400654* | 19 | 61880317 | 61927290 | 69.02 | 4.80E-03 | rs4940617 | 6.90E-04 |
| 12 | *TMBIM4* | 14 | 66530715 | 66563852 | 68.52 | 4.90E-03 | rs17767200 | 5.30E-04 |
| 5 | *ANXA6* | 87 | 150480266 | 150537443 | 223.36 | 5.20E-03 | rs1133202 | 8.70E-04 |
| 2 | *LINC00954* | 22 | 20068614 | 20084808 | 68.4 | 5.20E-03 | rs11096626 | 9.60E-04 |
| 20 | *LOC284801* | 3 | 26167654 | 26189869 | 13.16 | 5.30E-03 | rs12625395 | 6.60E-04 |
| 22 | *MAPK12* | 10 | 50691330 | 50700089 | 39.15 | 5.30E-03 | rs34422484 | 2.00E-04 |
| 22 | *CECR6* | 14 | 17597188 | 17602257 | 54.36 | 5.50E-03 | rs974396 | 6.10E-04 |
| 1 | *FMOD* | 18 | 203309748 | 203320557 | 73.86 | 5.50E-03 | rs12077300 | 1.30E-05 |
| 7 | *RBAK-RBAKDN* | 24 | 5085451 | 5112854 | 117.46 | 5.70E-03 | rs36026500 | 5.10E-04 |
| 7 | *RBAK* | 20 | 5085451 | 5109119 | 104.5 | 5.90E-03 | rs36026500 | 5.10E-04 |
| 19 | *MAST3* | 29 | 18208602 | 18262499 | 112.55 | 6.10E-03 | rs2072490 | 1.30E-04 |
| 12 | *STYK1* | 30 | 10771537 | 10826891 | 123.82 | 6.20E-03 | rs7297091 | 5.50E-05 |
| 9 | *NIPSNAP3A* | 8 | 107509968 | 107522403 | 30.29 | 6.30E-03 | rs1046115 | 5.00E-04 |
| 12 | *ATP2B1* | 18 | 89981825 | 90049844 | 47.22 | 6.40E-03 | rs1915109 | 8.40E-04 |
| 17 | *PIK3R5* | 35 | 8782232 | 8869029 | 122.28 | 6.60E-03 | rs61759594 | 6.00E-04 |
| 15 | *TTBK2* | 37 | 43036541 | 43213007 | 123.5 | 6.70E-03 | rs61433348 | 6.00E-04 |
| 17 | *TTLL6* | 28 | 46839592 | 46894469 | 99.26 | 6.90E-03 | rs2032844 | 7.10E-04 |
| 10 | *DPYSL4* | 13 | 134000413 | 134019280 | 42.87 | 7.20E-03 | rs2101613 | 8.00E-04 |
| 1 | *ATP8B2* | 14 | 154298035 | 154323780 | 62.67 | 7.50E-03 | rs3811452 | 2.10E-04 |
| 17 | *LOC100506713* | 21 | 6888441 | 6915653 | 90.99 | 7.80E-03 | rs11571353 | 7.70E-05 |
| 1 | *BSND* | 8 | 55464616 | 55474465 | 33.83 | 7.90E-03 | rs759914 | 3.20E-04 |
| 22 | *CRYBB3* | 9 | 25595816 | 25603326 | 33.45 | 8.00E-03 | rs2252880 | 4.80E-04 |
| 14 | *LINC00871* | 119 | 46533361 | 46971104 | 322.58 | 8.70E-03 | rs1761012 | 7.60E-05 |
| 1 | *MFSD4* | 32 | 205538111 | 205572046 | 89.66 | 8.90E-03 | rs1891506 | 4.00E-05 |
| 10 | *ARID5B* | 101 | 63661012 | 63856707 | 237.12 | 9.00E-03 | rs7901348 | 2.00E-05 |
| 2 | *PPP1R1C* | 74 | 182818967 | 182996109 | 200.6 | 9.20E-03 | rs6732434 | 1.30E-04 |
| 1 | *PRDM16* | 334 | 2985741 | 3355185 | 564.54 | 9.30E-03 | rs2993497 | 6.30E-05 |
| 17 | *CRHR1* | 11 | 43697709 | 43913194 | 35.9 | 9.50E-03 | rs12950522 | 1.60E-04 |
| 19 | *MED29* | 6 | 39881962 | 39891203 | 22.74 | 9.80E-03 | rs1629174 | 9.00E-04 |
| 15 | *SCG3* | 23 | 51973549 | 52013223 | 83.13 | 1.00E-02 | rs2607120 | 8.50E-04 |
| 18 | *CDH19* | 36 | 64168423 | 64271375 | 113.42 | 1.00E-02 | rs3764472 | 1.30E-04 |
| 9 | *PRUNE2* | 225 | 79226291 | 79521003 | 483.93 | 1.00E-02 | rs11145032 | 3.40E-04 |
| 9 | *LINC00475* | 10 | 94903748 | 94921890 | 43.13 | 1.00E-02 | rs118034474 | 6.10E-04 |
| 11 | *MICAL2* | 224 | 12132122 | 12285337 | 440.34 | 1.10E-02 | rs34300747 | 1.20E-04 |
| 16 | *MPG* | 6 | 127017 | 135850 | 26.4 | 1.10E-02 | rs710079 | 7.90E-04 |
| 1 | *CNIH3* | 102 | 224804178 | 224928249 | 253.08 | 1.10E-02 | rs12117838 | 1.00E-03 |
| 3 | *CD200R1L* | 21 | 112534555 | 112564797 | 75.6 | 1.30E-02 | rs6770923 | 9.80E-04 |
| 8 | *UBR5* | 29 | 103264501 | 103424917 | 85.08 | 1.40E-02 | rs10505029 | 1.10E-04 |
| 6 | *C6orf7* | 55 | 80513303 | 80580137 | 143.28 | 1.40E-02 | rs9352779 | 8.90E-05 |
| 2 | *XIRP2* | 187 | 167744996 | 168116261 | 395.41 | 1.40E-02 | rs16853051 | 9.40E-05 |
| 5 | *WDR55* | 8 | 140044383 | 140050553 | 24.02 | 1.40E-02 | rs2563312 | 1.80E-05 |
| 3 | *MAGI1* | 422 | 65339905 | 66024509 | 695.59 | 1.40E-02 | rs77275254 | 2.40E-04 |
| 15 | *FAM189A1* | 243 | 29412454 | 29862927 | 436.72 | 1.50E-02 | rs2336949 | 9.20E-04 |
| 1 | *PLEKHA6* | 84 | 204187978 | 204329057 | 243.2 | 1.50E-02 | rs4951353 | 9.60E-05 |
| 7 | *COA1* | 22 | 43670750 | 43769140 | 64.19 | 1.50E-02 | rs4724236 | 2.60E-04 |
| 10 | *TCERG1L* | 258 | 132890654 | 133109984 | 462.21 | 1.60E-02 | rs4750854 | 1.50E-05 |
| 10 | *MIR1256_10* | 49 | 74119697 | 74336541 | 156.03 | 1.60E-02 | rs7917581 | 9.60E-04 |
| 17 | *9-Sep* | 161 | 75277491 | 75496678 | 309.26 | 1.60E-02 | rs56981813 | 5.30E-04 |
| 11 | *PIWIL4* | 33 | 94300473 | 94354587 | 98.9 | 1.70E-02 | rs10437563 | 8.70E-04 |
| 2 | *SP140* | 32 | 231090444 | 231177930 | 92.13 | 1.70E-02 | rs3769847 | 2.30E-04 |
| 7 | *PPP1R9A* | 113 | 94536948 | 94925727 | 277.67 | 1.70E-02 | rs854543 | 1.70E-05 |
| 14 | *RHOJ* | 60 | 63671101 | 63760230 | 156.92 | 1.90E-02 | rs17224419 | 3.40E-04 |
| 2 | *EIF4E2* | 17 | 233415296 | 233448355 | 55.6 | 1.90E-02 | rs11899983 | 5.90E-04 |
| 17 | *GGA3* | 13 | 73232686 | 73258474 | 42.64 | 2.00E-02 | rs60360063 | 7.70E-04 |
| 6 | *FAM83B* | 48 | 54711568 | 54809897 | 138.43 | 2.00E-02 | rs1503139 | 5.50E-04 |
| 8 | *MFHAS1* | 78 | 8641998 | 8751131 | 193.95 | 2.10E-02 | rs10046783 | 3.50E-04 |
| 17 | *TSR1* | 6 | 2225981 | 2240678 | 16.74 | 2.20E-02 | rs413016 | 5.90E-04 |
| 9 | *DDX31* | 30 | 135469675 | 135545788 | 75.46 | 2.20E-02 | rs11243866 | 8.30E-04 |
| 12 | *FGD4* | 85 | 32655040 | 32798984 | 210.62 | 2.30E-02 | rs6488066 | 5.50E-04 |
| 9 | *ABCA1* | 200 | 107543283 | 107690527 | 377.19 | 2.30E-02 | rs10121901 | 9.20E-04 |
| 1 | *CASZ1* | 123 | 10696665 | 10856733 | 217.04 | 2.40E-02 | rs540329 | 2.10E-04 |
| 19 | *CERS4* | 47 | 8274216 | 8327304 | 94.39 | 2.50E-02 | rs36253 | 2.70E-04 |
| 9 | *ADAMTSL1* | 425 | 18474078 | 18910947 | 670.49 | 2.50E-02 | rs1368771 | 4.00E-04 |
| 3 | *SCAP* | 7 | 47455183 | 47517445 | 26.83 | 2.50E-02 | rs117953394 | 2.40E-04 |
| 13 | *PCDH9* | 362 | 66876965 | 67804468 | 621.15 | 2.60E-02 | rs1925753 | 5.80E-05 |
| 8 | *ERI1* | 9 | 8860313 | 8890849 | 23.58 | 2.70E-02 | rs73195802 | 7.00E-04 |
| 9 | *UNQ6494* | 68 | 92254697 | 92334674 | 138.12 | 2.80E-02 | rs73494255 | 3.00E-04 |
| 6 | *ANKRD6* | 69 | 90142896 | 90343553 | 156.71 | 2.90E-02 | rs6454764 | 3.30E-04 |
| 14 | *FAM179B* | 21 | 45431415 | 45543634 | 80.63 | 3.00E-02 | rs17115735 | 6.10E-05 |
| 7 | *DYNC1I1* | 199 | 95401817 | 95739634 | 355.45 | 3.00E-02 | rs17776584 | 1.30E-04 |
| 9 | *LOC100130954* | 25 | 138466770 | 138478958 | 70.71 | 3.10E-02 | rs78029836 | 2.20E-04 |
| 17 | *DNAH9* | 197 | 11501747 | 11873065 | 319.43 | 3.20E-02 | rs7208472 | 3.30E-05 |
| 9 | *BNC2* | 265 | 16409500 | 16870786 | 424.14 | 3.20E-02 | rs4961492 | 2.40E-04 |
| 13 | *RAB20* | 50 | 111175412 | 111214071 | 125.1 | 3.30E-02 | rs7489376 | 1.30E-05 |
| 15 | *AQP9* | 53 | 58430407 | 58478110 | 112.67 | 3.30E-02 | rs12440410 | 6.90E-04 |
| 6 | *LOC101927314* | 79 | 97753461 | 98156793 | 192.56 | 3.30E-02 | rs117910142 | 2.30E-05 |
| 2 | *PDE11A* | 165 | 178487976 | 178973066 | 355.11 | 3.30E-02 | rs2365622 | 3.60E-04 |
| 14 | *SERPINA9* | 34 | 94929057 | 94942670 | 71.58 | 3.50E-02 | rs4900232 | 9.60E-04 |
| 7 | *STK17A* | 20 | 43622691 | 43666978 | 49.61 | 3.50E-02 | rs12531841 | 4.70E-04 |
| 2 | *LINC00276* | 69 | 14368997 | 14541082 | 157.22 | 3.50E-02 | rs1900854 | 2.30E-04 |
| 13 | *SGCG* | 147 | 23755059 | 23899304 | 259.23 | 3.60E-02 | rs11148693 | 2.10E-04 |
| 13 | *COL4A2* | 297 | 110959630 | 111165373 | 454.44 | 3.60E-02 | rs9515194 | 7.20E-04 |
| 17 | *ALOX12* | 15 | 6899383 | 6914055 | 45.09 | 3.60E-02 | rs11571340 | 1.10E-04 |
| 23 | *GABRA3* | 33 | 151335633 | 151619831 | 74.17 | 3.80E-02 | rs1109840 | 5.40E-04 |
| 1 | *HHAT* | 270 | 210501595 | 210849638 | 434.33 | 3.80E-02 | rs6683572 | 4.80E-04 |
| 12 | *TMTC2* | 175 | 83080933 | 83528067 | 306.36 | 3.90E-02 | rs11115570 | 4.30E-04 |
| 10 | *EBF3* | 103 | 131633495 | 131762091 | 167.06 | 4.00E-02 | rs117693921 | 1.80E-04 |
| 15 | *RAB27A* | 45 | 55495163 | 55582013 | 104.89 | 4.20E-02 | rs4261468 | 9.50E-04 |
| 7 | *WIPF3* | 71 | 29846169 | 29956682 | 143.64 | 4.20E-02 | rs174945 | 3.00E-04 |
| 3 | *SLC9A9* | 348 | 142984063 | 143567373 | 520.37 | 4.30E-02 | rs6768181 | 9.70E-04 |
| 4 | *FBXL5* | 14 | 15606006 | 15657035 | 37.47 | 4.40E-02 | rs28393028 | 6.80E-04 |
| 16 | *HS3ST4* | 353 | 25703346 | 26149009 | 498.21 | 4.60E-02 | rs12373053 | 1.50E-04 |
| 15 | *PLA2G4F* | 19 | 42433331 | 42448839 | 38.63 | 4.70E-02 | rs10220859 | 6.50E-04 |
| 3 | *FGF12* | 377 | 191857181 | 192445388 | 529.45 | 4.70E-02 | rs2701595 | 6.10E-05 |
| 20 | *HAO1* | 25 | 7863630 | 7921093 | 62.57 | 4.80E-02 | rs6133511 | 1.50E-04 |
| 3 | *SEMA5B* | 128 | 122628039 | 122747452 | 219.04 | 4.80E-02 | rs57683123 | 9.40E-04 |
| 3 | *RBMS3* | 573 | 29322802 | 30051886 | 815.58 | 4.80E-02 | rs35159184 | 8.50E-04 |

**Supplementary Table S3-2**. The genes nominally associated with BEHLs at the frequency of 1.0 kHz from VEGAS2 gene-based analysis (*P* < 0.05)

| Chr | Gene | nSNPs | Start position | Stop position | Gene-based test statistic | P-value | Top-SNP | Top-SNP P-value |
| --- | --- | --- | --- | --- | --- | --- | --- | --- |
| 12 | *OR6C68* | 4 | 55886161 | 55887100 | 36.29 | 1.00E-05 | rs12579181 | 2.60E-04 |
| 12 | *OR6C70* | 6 | 55862983 | 55863922 | 29.97 | 3.00E-05 | rs58920821 | 5.50E-04 |
| 1 | *ADAMTS4* | 9 | 161159537 | 161168845 | 54.23 | 6.30E-05 | rs4233367 | 2.00E-04 |
| 20 | *LOC284801* | 3 | 26167654 | 26189869 | 23.09 | 7.00E-05 | rs12625395 | 3.70E-05 |
| 5 | *LOC101927488* | 10 | 125608209 | 125620867 | 70.66 | 1.30E-04 | rs62391798 | 2.30E-05 |
| 6 | *TRMT11* | 18 | 126307575 | 126360420 | 228.45 | 1.40E-04 | rs9375421 | 3.70E-06 |
| 6 | *HINT3* | 9 | 126277860 | 126301389 | 128.47 | 1.50E-04 | rs9375419 | 9.40E-05 |
| 9 | *RABEPK* | 6 | 127962820 | 127996438 | 41.53 | 2.00E-04 | rs599063 | 4.60E-04 |
| 21 | *COL18A1* | 102 | 46825096 | 46933634 | 391.21 | 2.40E-04 | rs8126757 | 8.70E-05 |
| 12 | *TAS2R9* | 5 | 10961692 | 10962767 | 19.92 | 2.50E-04 | rs3741845 | 2.90E-03 |
| 2 | *PSMD14* | 27 | 162164785 | 162268228 | 214.13 | 2.80E-04 | rs3769968 | 3.40E-04 |
| 4 | *PRMT9* | 5 | 148559533 | 148605280 | 18.86 | 3.10E-04 | rs77293186 | 7.50E-03 |
| 3 | *IQCF6* | 3 | 51812576 | 51813203 | 16.05 | 3.20E-04 | rs60093028 | 4.40E-03 |
| 1 | *AQP10* | 3 | 154293591 | 154297801 | 16.79 | 3.30E-04 | rs6685323 | 3.60E-03 |
| 12 | *TAS2R8* | 2 | 10958649 | 10959579 | 17.61 | 4.00E-04 | rs1548803 | 3.30E-04 |
| 19 | *NLRP13* | 47 | 56407310 | 56443702 | 208.52 | 4.20E-04 | rs12610617 | 3.40E-04 |
| 17 | *PDK2* | 9 | 48172100 | 48188733 | 72.68 | 4.50E-04 | rs705966 | 8.00E-05 |
| 11 | *MRPL16* | 3 | 59573607 | 59578345 | 18.12 | 4.60E-04 | rs2298589 | 4.80E-04 |
| 9 | *SHC3* | 37 | 91620685 | 91793682 | 168.14 | 4.70E-04 | rs944482 | 2.40E-03 |
| 9 | *PPAPDC3* | 10 | 134165080 | 134184649 | 66.66 | 5.50E-04 | rs1541122 | 1.50E-03 |
| 14 | *GALNT16* | 80 | 69726680 | 69821190 | 292.91 | 7.90E-04 | rs1147474 | 9.80E-05 |
| 7 | *CHST12* | 14 | 2443194 | 2474216 | 75.12 | 8.50E-04 | rs12536223 | 4.80E-04 |
| 13 | *DCUN1D2* | 22 | 114110133 | 114145023 | 105.5 | 9.30E-04 | rs9577555 | 6.80E-04 |
| 1 | *SLAMF7* | 21 | 160708846 | 160724608 | 106.7 | 9.70E-04 | rs2295619 | 7.30E-04 |
| 16 | *ITGAM* | 11 | 31271287 | 31344213 | 110.93 | 9.90E-04 | rs11150610 | 9.00E-04 |
| 9 | *DDX31* | 30 | 135469675 | 135545788 | 128.46 | 1.00E-03 | rs11243866 | 2.20E-05 |
| 15 | *ARID3B* | 12 | 74833547 | 74890472 | 71.65 | 1.10E-03 | rs10851873 | 1.90E-04 |
| 6 | *SLC22A3* | 88 | 160769404 | 160873611 | 419.65 | 1.10E-03 | rs2665357 | 6.40E-04 |
| 1 | *MCOLN2* | 24 | 85391265 | 85462796 | 104.22 | 1.20E-03 | rs634294 | 4.80E-04 |
| 15 | *SEMA7A* | 20 | 74701629 | 74726299 | 89.06 | 1.30E-03 | rs11857558 | 5.10E-05 |
| 17 | *PIK3R5* | 35 | 8782232 | 8869029 | 153.68 | 1.40E-03 | rs4791769 | 2.90E-04 |
| 23 | *STAG2* | 5 | 123094409 | 123236505 | 27.2 | 1.40E-03 | rs6649134 | 4.60E-04 |
| 6 | *SNX14* | 20 | 86215214 | 86303629 | 89.87 | 1.40E-03 | rs4304137 | 5.70E-04 |
| 4 | *LOC401127* | 4 | 39481874 | 39483523 | 18.38 | 1.40E-03 | rs117732912 | 5.50E-05 |
| 3 | *LINC00620* | 58 | 13692220 | 13788132 | 227 | 1.40E-03 | rs7650215 | 1.20E-04 |
| 17 | *ITGA3* | 18 | 48133339 | 48167849 | 83.58 | 1.50E-03 | rs2078864 | 7.40E-04 |
| 15 | *TLE3* | 44 | 70340129 | 70390256 | 163.27 | 1.60E-03 | rs34687476 | 4.30E-04 |
| 2 | *PQLC3* | 11 | 11295497 | 11318998 | 68.13 | 1.60E-03 | rs55691236 | 9.80E-04 |
| 23 | *GPR50* | 2 | 150345055 | 150349937 | 12.74 | 1.70E-03 | rs1202873 | 5.00E-04 |
| 9 | *BRINP1* | 80 | 121928907 | 122131739 | 298.89 | 1.80E-03 | rs10118663 | 3.30E-05 |
| 12 | *ALKBH2* | 2 | 109525992 | 109531293 | 12.62 | 1.90E-03 | rs76283368 | 6.40E-04 |
| 14 | *LINC00341* | 6 | 95873603 | 95876427 | 29.18 | 2.00E-03 | rs3742343 | 5.40E-04 |
| 18 | *LINC-ROR* | 8 | 54721803 | 54739350 | 40.08 | 2.00E-03 | rs4801076 | 4.90E-04 |
| 7 | *HNRNPA2B1* | 8 | 26229555 | 26240413 | 39.92 | 2.00E-03 | rs10499571 | 4.20E-04 |
| 11 | *MYBPC3* | 14 | 47352956 | 47374253 | 50.67 | 2.30E-03 | rs3729989 | 4.20E-04 |
| 15 | *C15orf26* | 9 | 81426643 | 81441516 | 32.67 | 2.30E-03 | rs9989357 | 6.90E-05 |
| 11 | *VSIG2* | 5 | 124617369 | 124622109 | 30.09 | 2.40E-03 | rs115910083 | 1.50E-04 |
| 11 | *NFRKB* | 16 | 129733669 | 129765490 | 73.91 | 2.40E-03 | rs1016364 | 4.10E-04 |
| 2 | *SCTR* | 48 | 120197418 | 120282028 | 183.33 | 2.50E-03 | rs2254122 | 2.00E-04 |
| 18 | *LOC400654* | 19 | 61880317 | 61927290 | 75.87 | 2.60E-03 | rs58575680 | 5.30E-04 |
| 18 | *LOC284294* | 185 | 61771324 | 62090827 | 445.75 | 2.60E-03 | rs58575680 | 5.30E-04 |
| 1 | *CSF1* | 14 | 110453232 | 110473616 | 63.31 | 2.60E-03 | rs1058885 | 5.40E-04 |
| 5 | *LOC101929154* | 54 | 77180479 | 77254920 | 166.75 | 2.90E-03 | rs4704464 | 5.50E-04 |
| 4 | *SLC7A11-AS1* | 26 | 139010167 | 139099331 | 131.55 | 2.90E-03 | rs13120260 | 4.00E-04 |
| 15 | *MEX3B* | 3 | 82334118 | 82338484 | 11.69 | 3.00E-03 | rs117387338 | 8.80E-04 |
| 20 | *APMAP* | 8 | 24943579 | 24973425 | 36.58 | 3.10E-03 | rs118059533 | 2.00E-04 |
| 3 | *HRG* | 13 | 186383746 | 186396023 | 55.9 | 3.10E-03 | rs10770 | 1.30E-04 |
| 22 | *MAPK12* | 10 | 50691330 | 50700089 | 42.59 | 3.30E-03 | rs34422484 | 6.00E-04 |
| 9 | *SCAI* | 56 | 127704887 | 127905838 | 223.93 | 3.30E-03 | rs643228 | 2.80E-04 |
| 8 | *SGK223* | 42 | 8175257 | 8239344 | 207.67 | 3.30E-03 | rs35038563 | 2.10E-04 |
| 2 | *NCK2* | 77 | 106361519 | 106510730 | 272.9 | 3.40E-03 | rs3769504 | 1.80E-04 |
| 2 | *SEMA4C* | 4 | 97525472 | 97535735 | 13.53 | 3.50E-03 | rs62152866 | 6.30E-04 |
| 11 | *SCN4B* | 21 | 118004091 | 118023630 | 89.47 | 3.70E-03 | rs11216781 | 7.30E-05 |
| 8 | *ST18* | 112 | 53023391 | 53322439 | 301.11 | 4.00E-03 | rs7824696 | 8.40E-04 |
| 12 | *GALNT9* | 96 | 132680916 | 132905905 | 231.13 | 4.10E-03 | rs28505774 | 1.80E-04 |
| 1 | *LRP8* | 62 | 53708040 | 53793821 | 172.56 | 4.20E-03 | rs12035926 | 2.70E-04 |
| 3 | *C3orf22* | 9 | 126268518 | 126277758 | 46.17 | 4.40E-03 | rs6782739 | 9.40E-04 |
| 14 | *LINC00523* | 14 | 101123604 | 101139081 | 57.53 | 4.50E-03 | rs8017950 | 5.10E-04 |
| 8 | *TNFRSF10B* | 40 | 22877647 | 22926700 | 119.33 | 4.60E-03 | rs4279595 | 4.30E-04 |
| 10 | *BMPR1A* | 35 | 88516395 | 88684945 | 148.71 | 4.70E-03 | rs4934272 | 1.30E-04 |
| 8 | *LONRF1* | 17 | 12579405 | 12612992 | 82.04 | 4.70E-03 | rs3802268 | 1.10E-04 |
| 1 | *ATP8B2* | 14 | 154298035 | 154323780 | 67.68 | 4.80E-03 | rs3811452 | 6.40E-04 |
| 6 | *NT5E* | 12 | 86159301 | 86205509 | 48.58 | 5.00E-03 | rs6922 | 5.70E-04 |
| 3 | *NR1I2* | 35 | 119499330 | 119537332 | 133.84 | 5.20E-03 | rs55987931 | 6.00E-04 |
| 11 | *CAND1.11* | 63 | 10329859 | 10452220 | 174.72 | 5.60E-03 | rs7934497 | 1.80E-04 |
| 12 | *TESC* | 36 | 117476727 | 117537251 | 122.55 | 5.60E-03 | rs10850747 | 6.70E-04 |
| 9 | *PPP6C* | 10 | 127908851 | 127952218 | 43.37 | 5.70E-03 | rs1048251 | 3.80E-04 |
| 17 | *SAMD14* | 11 | 48188672 | 48207246 | 44.46 | 5.80E-03 | rs2239953 | 5.60E-05 |
| 11 | *RPS3* | 8 | 75110534 | 75133345 | 34.45 | 6.10E-03 | rs532751 | 6.80E-04 |
| 12 | *USP30* | 15 | 109490379 | 109525831 | 54.4 | 6.40E-03 | rs16939904 | 6.40E-04 |
| 15 | *ZFAND6* | 24 | 80351909 | 80430735 | 82.83 | 6.60E-03 | rs1533857 | 5.60E-04 |
| 7 | *EGFR-AS1* | 18 | 55247442 | 55256642 | 82.33 | 6.60E-03 | rs13222385 | 8.00E-04 |
| 10 | *ADARB2* | 546 | 1223252 | 1779670 | 910.25 | 7.40E-03 | rs2387665 | 2.50E-05 |
| 12 | *FAM19A2* | 355 | 62102028 | 62586620 | 719.94 | 7.60E-03 | rs1348078 | 5.90E-04 |
| 11 | *MICAL2* | 224 | 12132122 | 12285337 | 466.12 | 7.70E-03 | rs1485957 | 5.90E-05 |
| 10 | *CCNYL2* | 21 | 42903621 | 42967688 | 76.98 | 7.90E-03 | rs2900957 | 2.50E-04 |
| 14 | *EXOC5* | 20 | 57669193 | 57735617 | 104.93 | 8.00E-03 | rs11849276 | 3.50E-04 |
| 4 | *HAND2-AS1* | 16 | 174451608 | 174462981 | 56.48 | 8.30E-03 | rs17059563 | 6.60E-04 |
| 23 | *GLRA2* | 24 | 14547419 | 14749933 | 92.65 | 8.70E-03 | rs2238912 | 2.70E-04 |
| 1 | *WARS2* | 43 | 119573838 | 119683295 | 129.11 | 9.00E-03 | rs12042797 | 6.10E-05 |
| 2 | *LOC101929512* | 8 | 162079768 | 162111154 | 22.91 | 9.30E-03 | rs3731769 | 9.60E-04 |
| 10 | *LGI1* | 28 | 95517565 | 95557916 | 89.8 | 9.70E-03 | rs10450368 | 8.90E-04 |
| 19 | *NLRP7* | 18 | 55434876 | 55458873 | 54.89 | 1.00E-02 | rs269950 | 9.30E-04 |
| 16 | *EMP2* | 66 | 10622278 | 10674539 | 147.81 | 1.10E-02 | rs2279868 | 9.60E-05 |
| 16 | *CHD9* | 60 | 53088944 | 53361414 | 166.3 | 1.10E-02 | rs3852742 | 3.40E-04 |
| 4 | *ABCA11P* | 9 | 419223 | 467998 | 40.36 | 1.10E-02 | rs11944122 | 5.30E-04 |
| 4 | *INPP4B* | 295 | 142949181 | 143767604 | 576.06 | 1.10E-02 | rs3775652 | 3.50E-04 |
| 2 | *DNMT3A* | 43 | 25455829 | 25565459 | 128.68 | 1.10E-02 | rs56197924 | 9.80E-04 |
| 19 | *CCDC61* | 21 | 46498718 | 46521874 | 59.4 | 1.20E-02 | rs759623 | 9.00E-04 |
| 2 | *LRP2* | 150 | 169983618 | 170219122 | 350.24 | 1.20E-02 | rs2075252 | 5.70E-04 |
| 9 | *CRB2* | 21 | 126118445 | 126142614 | 64.94 | 1.20E-02 | rs10760283 | 4.50E-04 |
| 12 | *SOX5* | 650 | 23685230 | 24715383 | 1008.6 | 1.30E-02 | rs16915574 | 8.30E-04 |
| 15 | *CERS3* | 117 | 100940599 | 101084925 | 371.68 | 1.30E-02 | rs12592841 | 2.20E-04 |
| 17 | *CRHR1* | 11 | 43697709 | 43913194 | 33.78 | 1.30E-02 | rs12950522 | 5.10E-04 |
| 17 | *NGFR* | 25 | 47572654 | 47592382 | 64.38 | 1.30E-02 | rs2584685 | 5.20E-04 |
| 8 | *GSDMD* | 5 | 144635556 | 144645231 | 19.74 | 1.30E-02 | rs149736517 | 2.20E-04 |
| 19 | *KLK3* | 14 | 51358170 | 51364020 | 51.25 | 1.40E-02 | rs12946 | 3.90E-04 |
| 14 | *LINC00871* | 119 | 46533361 | 46971104 | 295.69 | 1.50E-02 | rs6572357 | 7.50E-04 |
| 4 | *ZNF721* | 12 | 433772 | 492960 | 48.89 | 1.50E-02 | rs11944122 | 5.30E-04 |
| 11 | *TRIM5* | 32 | 5684424 | 5706339 | 88.44 | 1.60E-02 | rs11601507 | 3.10E-04 |
| 19 | *SMARCA4* | 32 | 11071597 | 11172958 | 90.37 | 1.60E-02 | rs7248790 | 5.30E-04 |
| 19 | *NUP62* | 22 | 50410083 | 50432988 | 65.44 | 1.60E-02 | rs1152232 | 3.30E-04 |
| 20 | *C20orf196* | 72 | 5731042 | 5844559 | 152.85 | 1.60E-02 | rs237076 | 6.40E-04 |
| 2 | *SP140* | 32 | 231090444 | 231177930 | 92.46 | 1.60E-02 | rs3769847 | 1.80E-05 |
| 2 | *NPAS2* | 144 | 101436612 | 101613287 | 282.46 | 1.60E-02 | rs12472321 | 3.80E-04 |
| 1 | *PRDM16* | 334 | 2985741 | 3355185 | 538.9 | 1.60E-02 | rs2993502 | 2.10E-04 |
| 14 | *TEP1* | 59 | 20833825 | 20881579 | 131.88 | 1.70E-02 | rs55790286 | 4.40E-04 |
| 17 | *UBALD2* | 6 | 74261285 | 74267379 | 23.91 | 1.70E-02 | rs9902837 | 8.10E-04 |
| 17 | *DNAH9* | 197 | 11501747 | 11873065 | 347.13 | 1.70E-02 | rs8071438 | 5.60E-04 |
| 18 | *ATP8B1* | 111 | 55313658 | 55470327 | 235.65 | 1.70E-02 | rs67785935 | 2.00E-04 |
| 8 | *ST3GAL1* | 122 | 134467090 | 134584183 | 245.24 | 1.70E-02 | rs28445987 | 9.70E-05 |
| 13 | *SPATA13_2* | 154 | 24734854 | 24881212 | 272.47 | 1.80E-02 | rs12585910 | 3.80E-04 |
| 15 | *LOC102723320* | 24 | 100913143 | 100978119 | 85.77 | 1.80E-02 | rs2587829 | 3.90E-04 |
| 16 | *SRL* | 42 | 4239374 | 4292081 | 101.06 | 1.80E-02 | rs879150 | 3.20E-04 |
| 2 | *XIRP2* | 187 | 167744996 | 168116261 | 377.67 | 1.80E-02 | rs1511199 | 8.90E-04 |
| 6 | *HDDC2* | 10 | 125596495 | 125623282 | 34.2 | 1.80E-02 | rs3778451 | 7.60E-04 |
| 14 | *SYT16* | 99 | 62462540 | 62568427 | 225.9 | 1.90E-02 | rs1254917 | 1.50E-04 |
| 11 | *NAV2* | 631 | 19372270 | 20143147 | 923.01 | 2.00E-02 | rs9667610 | 1.50E-04 |
| 12 | *LOC643339* | 195 | 93397533 | 93771512 | 351.18 | 2.10E-02 | rs11106865 | 7.70E-06 |
| 2 | *AFF3* | 156 | 100163715 | 100759037 | 322.37 | 2.10E-02 | rs12464362 | 2.20E-04 |
| 4 | *LRBA* | 164 | 151185810 | 151936649 | 370.09 | 2.10E-02 | rs75635195 | 3.50E-04 |
| 3 | *MAGI1* | 422 | 65339905 | 66024509 | 668.56 | 2.20E-02 | rs1917527 | 2.30E-04 |
| 7 | *LINC-PINT_2* | 91 | 130626518 | 130794675 | 175.9 | 2.20E-02 | rs3929114 | 3.80E-05 |
| 19 | *U2AF2* | 11 | 56165415 | 56186082 | 35.45 | 2.30E-02 | rs2271757 | 8.70E-04 |
| 13 | *LINC00403* | 77 | 112626623 | 112762329 | 165.91 | 2.40E-02 | rs7988612 | 6.40E-04 |
| 3 | *C3orf20* | 58 | 14716605 | 14814543 | 128.48 | 2.40E-02 | rs4312639 | 1.80E-04 |
| 10 | *PFKFB3* | 131 | 6186842 | 6277507 | 220.89 | 2.50E-02 | rs4750065 | 3.00E-04 |
| 10 | *ANK3* | 337 | 61786055 | 62493284 | 576.22 | 2.50E-02 | rs7068443 | 2.10E-04 |
| 16 | *TELO2* | 13 | 1543351 | 1560460 | 31.1 | 2.50E-02 | rs2745108 | 6.30E-04 |
| 20 | *SPTLC3* | 98 | 12989626 | 13147411 | 211.93 | 2.50E-02 | rs171500 | 3.60E-04 |
| 20 | *RASSF2* | 78 | 4760668 | 4804291 | 171.13 | 2.50E-02 | rs2422993 | 5.00E-04 |
| 1 | *FMOD* | 18 | 203309748 | 203320557 | 54.03 | 2.50E-02 | rs12077300 | 3.10E-04 |
| 18 | *LDLRAD4* | 350 | 13218728 | 13652753 | 517.77 | 2.60E-02 | rs1284427 | 3.20E-04 |
| 6 | *MIR548H3_6* | 60 | 97537842 | 97862283 | 157.21 | 2.60E-02 | rs2388041 | 4.60E-05 |
| 3 | *ABI3BP* | 117 | 100468178 | 100712334 | 226.2 | 2.60E-02 | rs12330346 | 1.70E-04 |
| 9 | *MUSK* | 83 | 113431050 | 113563278 | 169.24 | 2.70E-02 | rs2766984 | 3.90E-04 |
| 12 | *ANO2* | 244 | 5671816 | 6055398 | 425.01 | 2.80E-02 | rs1558776 | 6.90E-04 |
| 13 | *CCDC168* | 25 | 103381716 | 103411422 | 82.06 | 2.80E-02 | rs12855785 | 8.10E-04 |
| 19 | *ZNF880* | 16 | 52873169 | 52889046 | 46.37 | 2.80E-02 | rs17780009 | 6.00E-04 |
| 19 | *IL4I1* | 33 | 50392912 | 50432796 | 73.27 | 2.80E-02 | rs1152232 | 3.30E-04 |
| 20 | *ADRM1* | 6 | 60877951 | 60883918 | 16.27 | 3.00E-02 | rs3088238 | 6.90E-04 |
| 9 | *FRMD3* | 226 | 85857904 | 86153348 | 400.28 | 3.00E-02 | rs10868000 | 9.90E-04 |
| 9 | *GNA14* | 171 | 80037994 | 80263232 | 286.35 | 3.00E-02 | rs17063807 | 8.70E-05 |
| 19 | *MAST3* | 29 | 18208602 | 18262499 | 77.12 | 3.10E-02 | rs11086092 | 6.50E-04 |
| 15 | *IL16* | 70 | 81489218 | 81605104 | 152.55 | 3.20E-02 | rs11556218 | 9.50E-04 |
| 7 | *EGFR* | 110 | 55086724 | 55275031 | 209.26 | 3.30E-02 | rs2293348 | 3.60E-04 |
| 5 | *RBM27* | 14 | 145583162 | 145668784 | 46.33 | 3.30E-02 | rs117259215 | 5.80E-04 |
| 10 | *ARID5B* | 101 | 63661012 | 63856707 | 188.74 | 3.40E-02 | rs12569576 | 1.40E-04 |
| 12 | *AVIL* | 5 | 58191159 | 58209852 | 15.58 | 3.40E-02 | rs2277325 | 5.50E-04 |
| 5 | *AQPEP* | 105 | 115298150 | 115363299 | 213.88 | 3.40E-02 | rs2662482 | 2.90E-07 |
| 2 | *CERKL* | 48 | 182401400 | 182521834 | 112.19 | 3.40E-02 | rs3910660 | 4.50E-04 |
| 18 | *CABLES1* | 60 | 20714527 | 20840434 | 125.95 | 3.50E-02 | rs4800451 | 8.90E-04 |
| 1 | *NDUFS2* | 10 | 161169104 | 161184184 | 26.88 | 3.50E-02 | rs10797094 | 6.40E-04 |
| 9 | *TLE1* | 95 | 84198597 | 84303596 | 161.35 | 3.60E-02 | rs57208048 | 2.30E-04 |
| 2 | *SP140L* | 53 | 231191893 | 231268445 | 117.23 | 3.80E-02 | rs16827136 | 1.90E-04 |
| 1 | *KCNH1* | 216 | 210851656 | 211307457 | 384.81 | 3.80E-02 | rs17017087 | 2.40E-05 |
| 2 | *CAPN14* | 43 | 31395921 | 31440411 | 112.74 | 3.90E-02 | rs72797144 | 2.80E-04 |
| 19 | *LOC284395* | 187 | 29777917 | 30016659 | 336.51 | 4.00E-02 | rs892073 | 5.50E-04 |
| 1 | *COL24A1* | 202 | 86194915 | 86622121 | 350.32 | 4.20E-02 | rs6665375 | 5.80E-04 |
| 10 | *CHST15* | 75 | 125767181 | 125853123 | 144.55 | 4.30E-02 | rs6588752 | 4.70E-04 |
| 9 | *GTF3C4* | 12 | 135545727 | 135565470 | 33.3 | 4.30E-02 | rs11788764 | 2.10E-05 |
| 11 | *TMEM45B* | 19 | 129685740 | 129729898 | 54.03 | 4.60E-02 | rs536393 | 8.50E-05 |
| 12 | *TMEM132C* | 397 | 128751947 | 129192460 | 535.62 | 4.60E-02 | rs4322463 | 7.80E-05 |

**Supplementary Table S3-3**. The genes nominally associated with BEHLs at the frequency of 2.0 kHz from VEGAS2 gene-based analysis (*P* < 0.05)

| Chr | Gene | nSNPs | Start position | Stop position | Gene-based test statistic | P-value | Top-SNP | Top-SNP P-value |
| --- | --- | --- | --- | --- | --- | --- | --- | --- |
| 10 | *SLC16A9* | 34 | 61410521 | 61469649 | 333.71 | 1.20E-05 | rs3763747 | 1.90E-06 |
| 11 | *UBQLN3* | 7 | 5528529 | 5531153 | 44.17 | 1.20E-05 | rs2234456 | 9.80E-07 |
| 15 | *ARID3B* | 12 | 74833547 | 74890472 | 111.9 | 4.40E-05 | rs10851873 | 3.00E-05 |
| 11 | *MYBPC3* | 14 | 47352956 | 47374253 | 82.19 | 5.80E-05 | rs11570058 | 1.40E-07 |
| 15 | *UBL7* | 5 | 74738317 | 74753529 | 76.07 | 1.10E-04 | rs11854461 | 9.50E-05 |
| 21 | *LTN1* | 26 | 30300465 | 30365277 | 146.62 | 1.20E-04 | rs2248903 | 1.20E-03 |
| 15 | *SEMA7A* | 20 | 74701629 | 74726299 | 119.02 | 1.40E-04 | rs11857558 | 2.60E-05 |
| 15 | *CLK3* | 7 | 74900712 | 74922542 | 56.58 | 2.00E-04 | rs2068982 | 7.50E-05 |
| 17 | *LLGL2* | 42 | 73521782 | 73571290 | 230.79 | 2.60E-04 | rs11652563 | 4.00E-04 |
| 15 | *EDC3* | 8 | 74922898 | 74988386 | 62.22 | 2.90E-04 | rs11072498 | 1.10E-04 |
| 18 | *LAMA3* | 67 | 21269561 | 21535029 | 375.48 | 3.20E-04 | rs12608087 | 1.60E-04 |
| 11 | *OR51I2* | 6 | 5474637 | 5475707 | 31.88 | 4.00E-04 | rs11037503 | 1.20E-06 |
| 2 | *CEBPZ* | 10 | 37428774 | 37458740 | 64.05 | 4.50E-04 | rs3213746 | 9.80E-04 |
| 21 | *MAP3K7CL* | 45 | 30449791 | 30548210 | 219.65 | 4.70E-04 | rs2832231 | 8.00E-05 |
| 1 | *TTF2* | 25 | 117602948 | 117645491 | 126.14 | 4.80E-04 | rs2274253 | 1.00E-03 |
| 3 | *CLSTN2-AS1* | 2 | 140224459 | 140227631 | 15.77 | 5.00E-04 | rs7615158 | 4.80E-03 |
| 5 | *ZDHHC11* | 5 | 795719 | 851101 | 24.71 | 5.60E-04 | rs67757774 | 3.20E-03 |
| 15 | *TMOD3* | 16 | 52121824 | 52204331 | 125.71 | 6.10E-04 | rs7163541 | 2.20E-04 |
| 1 | *FAM212B* | 28 | 112264685 | 112298419 | 124.86 | 6.10E-04 | rs41314013 | 1.40E-04 |
| 1 | *ADAMTS4* | 9 | 161159537 | 161168845 | 41.94 | 6.10E-04 | rs7512012 | 1.10E-03 |
| 1 | *MAGOH* | 7 | 53692563 | 53704282 | 52.99 | 6.30E-04 | rs2275087 | 4.60E-04 |
| 1 | *FAM212B-AS1* | 4 | 112282462 | 112290420 | 24.43 | 6.60E-04 | rs2295044 | 3.30E-04 |
| 2 | *SEMA4C* | 4 | 97525472 | 97535735 | 17.23 | 6.70E-04 | rs62152866 | 4.20E-03 |
| 1 | *C1orf123* | 3 | 53679771 | 53686289 | 34.04 | 7.20E-04 | rs1056438 | 6.40E-04 |
| 4 | *INPP4B* | 295 | 142949181 | 143767604 | 766.27 | 7.30E-04 | rs2636645 | 4.30E-04 |
| 23 | *FAM58A* | 4 | 152853382 | 152864632 | 28.57 | 7.90E-04 | rs5945317 | 9.30E-04 |
| 5 | *SRP19* | 20 | 112196884 | 112228776 | 133.03 | 8.70E-04 | rs74845632 | 1.00E-04 |
| 17 | *C17orf49* | 5 | 6918055 | 6920843 | 33.58 | 9.60E-04 | rs11078662 | 3.10E-03 |
| 15 | *SCAMP5* | 9 | 75287875 | 75313836 | 46.74 | 1.00E-03 | rs3812944 | 7.50E-04 |
| 4 | *ASB5* | 45 | 177134825 | 177190373 | 194.64 | 1.10E-03 | rs6827525 | 1.30E-04 |
| 1 | *DDX20* | 9 | 112298189 | 112310199 | 41.63 | 1.20E-03 | rs75494293 | 1.80E-04 |
| 15 | *PSTPIP1* | 28 | 77287464 | 77329671 | 144.15 | 1.30E-03 | rs35134156 | 2.70E-04 |
| 19 | *DPRX* | 3 | 54135309 | 54140263 | 19.14 | 1.30E-03 | rs11669354 | 6.70E-05 |
| 5 | *LOC101927488* | 10 | 125608209 | 125620867 | 53.35 | 1.30E-03 | rs62391798 | 1.90E-05 |
| 6 | *MIR548H3_6* | 60 | 97537842 | 97862283 | 257.94 | 1.40E-03 | rs35348105 | 1.50E-04 |
| 2 | *MARCO* | 29 | 119699744 | 119752236 | 136.25 | 1.40E-03 | rs6752783 | 3.50E-04 |
| 12 | *UNC119B* | 8 | 121148237 | 121161443 | 37.82 | 1.50E-03 | rs61229807 | 9.10E-04 |
| 19 | *ZNF816* | 10 | 53452631 | 53466164 | 42.84 | 1.60E-03 | rs57088011 | 7.50E-05 |
| 22 | *NHP2L1* | 5 | 42069936 | 42084913 | 24.51 | 1.60E-03 | rs6002436 | 3.20E-04 |
| 2 | *ANKRD53* | 12 | 71205574 | 71212629 | 62.16 | 1.60E-03 | rs113707068 | 6.40E-04 |
| 21 | *LOC100506403* | 90 | 36744804 | 36953062 | 299.9 | 1.70E-03 | rs2835009 | 7.90E-04 |
| 23 | *PTCHD1-AS* | 308 | 22277913 | 23311263 | 612.55 | 1.70E-03 | rs6633657 | 1.20E-08 |
| 2 | *PRKD3* | 19 | 37477645 | 37544222 | 126.6 | 1.70E-03 | rs12469082 | 9.90E-04 |
| 2 | *NIFK-AS1* | 16 | 122407229 | 122486136 | 99.2 | 1.80E-03 | rs11886007 | 4.50E-04 |
| 17 | *GGA3* | 13 | 73232686 | 73258474 | 68.81 | 1.90E-03 | rs60360063 | 7.90E-04 |
| 4 | *TLR1* | 9 | 38797875 | 38806412 | 32.64 | 1.90E-03 | rs4072548 | 9.60E-04 |
| 15 | *LEO1* | 11 | 52230221 | 52263998 | 49.62 | 2.00E-03 | rs2959295 | 4.50E-04 |
| 1 | *WARS2* | 43 | 119573838 | 119683295 | 162.24 | 2.10E-03 | rs12042797 | 2.70E-04 |
| 1 | *KMO* | 53 | 241695433 | 241758949 | 145.87 | 2.10E-03 | rs12139931 | 6.40E-04 |
| 11 | *SART1* | 9 | 65729159 | 65747607 | 44.87 | 2.20E-03 | rs552130 | 9.60E-04 |
| 12 | *GNPTAB* | 27 | 102139274 | 102224645 | 108.89 | 2.20E-03 | rs10860794 | 1.60E-04 |
| 14 | *PCK2* | 9 | 24563339 | 24573341 | 35.96 | 2.20E-03 | rs9783666 | 3.90E-05 |
| 23 | *NHS* | 27 | 17393542 | 17754113 | 91.55 | 2.20E-03 | rs5955543 | 4.10E-04 |
| 3 | *LIMD1-AS1* | 5 | 45719656 | 45730374 | 30.7 | 2.20E-03 | rs2742413 | 7.20E-04 |
| 2 | *EN1* | 2 | 119599746 | 119605759 | 12.32 | 2.50E-03 | rs79126103 | 4.50E-04 |
| 18 | *SETBP1* | 176 | 42260137 | 42648475 | 438.61 | 2.60E-03 | rs687781 | 4.00E-04 |
| 6 | *LOC101927314* | 79 | 97753461 | 98156793 | 317.35 | 2.80E-03 | rs9320518 | 9.30E-04 |
| 15 | *HCN4* | 29 | 73612199 | 73661605 | 105.3 | 3.00E-03 | rs4777599 | 1.10E-04 |
| 3 | *C3orf20* | 58 | 14716605 | 14814543 | 176.78 | 3.00E-03 | rs7636830 | 3.80E-04 |
| 5 | *CEP72* | 16 | 612404 | 653666 | 66.63 | 3.00E-03 | rs76242473 | 9.40E-04 |
| 2 | *SLC30A6* | 12 | 32390909 | 32449181 | 77.48 | 3.00E-03 | rs177083 | 2.40E-04 |
| 12 | *SPPL3* | 32 | 121200312 | 121342155 | 138.63 | 3.30E-03 | rs7307973 | 8.70E-05 |
| 3 | *FYTTD1* | 6 | 197476423 | 197511317 | 40.75 | 3.40E-03 | rs78851703 | 7.70E-04 |
| 18 | *SLMO1* | 14 | 12407894 | 12432236 | 66.83 | 3.50E-03 | rs1785658 | 8.00E-04 |
| 15 | *RASGRP1* | 26 | 38780301 | 38857007 | 95.47 | 3.70E-03 | rs7403047 | 1.80E-04 |
| 3 | *SLC22A14* | 27 | 38347444 | 38359859 | 100.39 | 3.70E-03 | rs2070492 | 6.20E-04 |
| 21 | *IFNAR1* | 12 | 34697213 | 34732128 | 71.72 | 3.80E-03 | rs2243600 | 7.10E-05 |
| 19 | *PEPD* | 85 | 33877854 | 34012799 | 306.57 | 4.20E-03 | rs10425678 | 4.00E-04 |
| 20 | *LOC643406* | 6 | 5451841 | 5457780 | 28.74 | 4.20E-03 | rs6139738 | 4.20E-04 |
| 17 | *STX8* | 180 | 9153787 | 9479275 | 371.54 | 4.40E-03 | rs12945819 | 1.60E-04 |
| 10 | *MIR1256_10* | 49 | 74119697 | 74336541 | 208.86 | 4.50E-03 | rs7917581 | 9.90E-05 |
| 1 | *IBA57* | 8 | 228353428 | 228369958 | 25.74 | 4.50E-03 | rs1934882 | 6.50E-06 |
| 12 | *GALNT9* | 96 | 132680916 | 132905905 | 229.79 | 4.60E-03 | rs10794457 | 2.20E-04 |
| 3 | *FGF12* | 377 | 191857181 | 192445388 | 652.5 | 4.60E-03 | rs1574091 | 5.60E-04 |
| 20 | *C20orf196* | 72 | 5731042 | 5844559 | 178.97 | 4.70E-03 | rs237075 | 2.70E-04 |
| 11 | *OR51B5* | 196 | 5362112 | 5526882 | 541.51 | 4.80E-03 | rs1498482 | 1.10E-06 |
| 20 | *SLX4IP* | 48 | 10415950 | 10604027 | 169.64 | 4.80E-03 | rs13044584 | 3.80E-04 |

**Supplementary Table S3-4**. The genes nominally associated with BEHLs at the frequency of 4.0 kHz from VEGAS2 gene-based analysis (*P* < 0.05)

| Chr | Gene | nSNPs | Start position | Stop position | Gene-based test statistic | P-value | Top-SNP | Top-SNP P-value |
| --- | --- | --- | --- | --- | --- | --- | --- | --- |
| 8 | *LOC101929268* | 58 | 49464126 | 49611069 | 297.37 | 1.20E-05 | rs6983178 | 1.80E-05 |
| 23 | *PIR-FIGF* | 18 | 15363712 | 15509432 | 193.95 | 1.60E-05 | rs2071177 | 1.70E-05 |
| 23 | *PIGA* | 5 | 15337572 | 15353676 | 82.19 | 3.70E-05 | rs3661 | 4.50E-05 |
| 12 | *GLTP* | 11 | 110288747 | 110318293 | 100.54 | 5.70E-05 | rs10850913 | 1.00E-06 |
| 2 | *HNMT* | 23 | 138721807 | 138773934 | 225.72 | 8.50E-05 | rs3828168 | 3.00E-05 |
| 19 | *FUT3* | 7 | 5842898 | 5851485 | 38.74 | 1.00E-04 | rs812936 | 2.10E-03 |
| 23 | *PIR* | 17 | 15402923 | 15511711 | 187.85 | 1.10E-04 | rs2071177 | 1.70E-05 |
| 9 | *GTF3C5* | 9 | 135906061 | 135933890 | 72.65 | 1.40E-04 | rs1541331 | 1.70E-04 |
| 4 | *CCNI* | 8 | 77969176 | 77997125 | 52.91 | 1.70E-04 | rs4252786 | 3.70E-04 |
| 10 | *CRTAC1* | 82 | 99624756 | 99790585 | 329.79 | 1.80E-04 | rs4919154 | 1.20E-04 |
| 2 | *LOC100507006* | 11 | 64455534 | 64479665 | 89.99 | 1.90E-04 | rs1025209 | 1.50E-04 |
| 23 | *ASB11* | 4 | 15299830 | 15333746 | 42.55 | 2.20E-04 | rs5935944 | 4.50E-05 |
| 8 | *KLHL38* | 20 | 124657914 | 124665190 | 146.13 | 2.70E-04 | rs11784192 | 2.40E-04 |
| 23 | *IL1RAPL1* | 151 | 28605680 | 29974017 | 425.67 | 2.90E-04 | rs5972128 | 4.50E-05 |
| 16 | *APRT* | 2 | 88875876 | 88878342 | 18.71 | 3.10E-04 | rs8191483 | 6.80E-04 |
| 1 | *CPT2* | 11 | 53662100 | 53679869 | 47.34 | 3.20E-04 | rs11578832 | 3.00E-04 |
| 12 | *CLEC2A* | 11 | 10065825 | 10084980 | 64.79 | 3.30E-04 | rs644565 | 2.50E-04 |
| 17 | *BZRAP1-AS1* | 17 | 56402810 | 56431088 | 103.8 | 3.90E-04 | rs2632527 | 1.60E-04 |
| 5 | *GRIA1* | 140 | 152870083 | 153193429 | 430.81 | 4.10E-04 | rs549100 | 5.50E-04 |
| 6 | *LOC100130357* | 17 | 13279526 | 13295818 | 103.97 | 4.80E-04 | rs1550526 | 2.60E-04 |
| 23 | *FIGF* | 4 | 15363712 | 15402535 | 27.38 | 5.40E-04 | rs6632521 | 6.00E-04 |
| 18 | *LAMA3* | 67 | 21269561 | 21535029 | 347.65 | 5.50E-04 | rs12185288 | 1.90E-04 |
| 8 | *EFCAB1* | 4 | 49627473 | 49647870 | 35.4 | 5.80E-04 | rs1126259 | 4.10E-04 |
| 9 | *FNBP1* | 36 | 132649465 | 132805473 | 190.8 | 7.20E-04 | rs10739766 | 9.10E-04 |
| 4 | *11-Sep* | 36 | 77870894 | 77959768 | 177.48 | 8.90E-04 | rs10213389 | 3.90E-04 |
| 17 | *C17orf102* | 10 | 32901141 | 32906388 | 53.58 | 9.00E-04 | rs9891306 | 7.80E-05 |
| 17 | *GPATCH8* | 18 | 42472644 | 42580957 | 101.11 | 9.80E-04 | rs3744427 | 3.70E-04 |
| 4 | *NAA11* | 2 | 80238271 | 80247171 | 14.37 | 9.90E-04 | rs13106792 | 3.00E-04 |
| 17 | *BTBD17* | 8 | 72352554 | 72357958 | 46.69 | 1.00E-03 | rs11650943 | 4.40E-04 |
| 9 | *GPR107* | 33 | 132815984 | 132902448 | 142.02 | 1.20E-03 | rs2159640 | 5.20E-04 |
| 4 | *FGB* | 9 | 155484131 | 155493915 | 56.51 | 1.20E-03 | rs2227412 | 7.20E-04 |
| 4 | *LINC01095* | 5 | 147030606 | 147043065 | 37.64 | 1.20E-03 | rs11722991 | 3.20E-04 |
| 1 | *KIAA1804* | 50 | 233463513 | 233520894 | 278.7 | 1.40E-03 | rs4649220 | 6.30E-05 |
| 17 | *RNF43* | 22 | 56431037 | 56494931 | 104.97 | 1.50E-03 | rs2257205 | 7.00E-04 |
| 17 | *CRYBA1* | 2 | 27573874 | 27581502 | 19.36 | 1.50E-03 | rs1047790 | 4.70E-04 |
| 20 | *LOC100270804* | 2 | 18774692 | 18776709 | 13.18 | 1.50E-03 | rs73601851 | 2.90E-04 |
| 3 | *DYNC1LI1* | 12 | 32567462 | 32612366 | 79.89 | 1.50E-03 | rs6782149 | 7.50E-04 |
| 17 | *NUFIP2* | 10 | 27582853 | 27621166 | 48.67 | 1.60E-03 | rs7214290 | 4.70E-04 |
| 1 | *CATSPER4* | 15 | 26517118 | 26529033 | 75.91 | 1.60E-03 | rs12732367 | 7.50E-04 |
| 12 | *MRPL51* | 4 | 6601315 | 6602471 | 31.82 | 1.70E-03 | rs1558334 | 6.30E-04 |
| 18 | *ZNF521* | 162 | 22641887 | 22932214 | 396.33 | 1.70E-03 | rs7235504 | 2.60E-04 |
| 2 | *KYNU* | 67 | 143635194 | 143799885 | 233.29 | 1.70E-03 | rs351685 | 1.20E-05 |
| 8 | *POMK* | 3 | 42948648 | 42978323 | 16.8 | 1.70E-03 | rs7001861 | 7.90E-04 |
| 4 | *FNIP2* | 19 | 159690181 | 159827954 | 96.99 | 1.90E-03 | rs12642474 | 2.00E-04 |
| 17 | *TRIM37* | 21 | 57059999 | 57184266 | 106.74 | 2.00E-03 | rs7214402 | 5.20E-04 |
| 19 | *NOTCH3* | 16 | 15270443 | 15311792 | 83.11 | 2.00E-03 | rs1044009 | 9.50E-04 |
| 2 | *COL4A4* | 90 | 227867426 | 228029275 | 280.51 | 2.00E-03 | rs13411764 | 7.70E-04 |
| 16 | *USP31* | 19 | 23072727 | 23160591 | 115.02 | 2.10E-03 | rs4968044 | 9.40E-04 |
| 14 | *SLC25A21* | 305 | 37147125 | 37641865 | 655.73 | 2.20E-03 | rs10143832 | 1.70E-04 |
| 17 | *RAD51C* | 9 | 56769933 | 56811703 | 50.6 | 2.20E-03 | rs17222733 | 7.00E-04 |
| 1 | *MRPS14* | 7 | 174982093 | 174992591 | 36.38 | 2.20E-03 | rs16847706 | 5.10E-04 |
| 8 | *LRRC6* | 23 | 133584200 | 133687863 | 111.26 | 2.30E-03 | rs7834760 | 3.50E-04 |
| 8 | *POTEA* | 8 | 43147584 | 43218328 | 47.94 | 2.40E-03 | rs1993999 | 9.90E-04 |
| 8 | *FNTA* | 3 | 42911441 | 42940932 | 17.38 | 2.40E-03 | rs10958736 | 8.10E-04 |
| 15 | *LOC101929743* | 9 | 89128801 | 89148341 | 51.95 | 2.50E-03 | rs55652124 | 7.70E-04 |
| 17 | *TEX14* | 28 | 56634037 | 56769416 | 142.31 | 2.50E-03 | rs8071217 | 7.50E-04 |
| 21 | *LOC101927821* | 39 | 23119304 | 23169735 | 150.29 | 2.60E-03 | rs957575 | 1.30E-04 |
| 19 | *ADAMTSL5* | 6 | 1505016 | 1513188 | 25.43 | 2.70E-03 | rs265296 | 5.30E-05 |
| 4 | *MEPE* | 28 | 88742549 | 88767968 | 99.62 | 2.80E-03 | rs11097172 | 8.80E-04 |
| 2 | *KCNJ3* | 91 | 155555092 | 155714864 | 258.18 | 2.90E-03 | rs3111033 | 2.40E-04 |
| 1 | *CD34* | 21 | 208059882 | 208084683 | 87.67 | 3.00E-03 | rs2259397 | 1.60E-04 |
| 6 | *ANKS1A* | 66 | 34857037 | 35059190 | 286.54 | 3.10E-03 | rs2293242 | 6.20E-04 |
| 2 | *TRIP12* | 22 | 230628552 | 230786725 | 90.01 | 3.60E-03 | rs75777414 | 8.50E-04 |
| 12 | *SLCO1B7* | 31 | 21168629 | 21243040 | 144.1 | 3.70E-03 | rs7133651 | 4.90E-04 |
| 17 | *CD300LF* | 15 | 72690446 | 72709139 | 53.84 | 3.90E-03 | rs35489971 | 4.20E-04 |
| 3 | *CCDC80* | 31 | 112323232 | 112359990 | 101.59 | 3.90E-03 | rs13084615 | 8.90E-04 |
| 19 | *ZNF701* | 22 | 53073525 | 53090427 | 68.01 | 4.20E-03 | rs117787021 | 4.80E-04 |
| 4 | *GC* | 38 | 72607410 | 72671237 | 123.26 | 4.20E-03 | rs1491709 | 5.50E-05 |
| 4 | *ASB5* | 45 | 177134825 | 177190373 | 159.68 | 4.30E-03 | rs10032098 | 7.80E-04 |
| 17 | *PPM1E* | 35 | 56833229 | 57062540 | 173.32 | 4.80E-03 | rs6503884 | 9.40E-04 |
| 2 | *HECW2* | 159 | 197063976 | 197457335 | 414.72 | 4.90E-03 | rs1563379 | 7.60E-05 |
| 9 | *GLDC* | 68 | 6532463 | 6645692 | 180.41 | 5.00E-03 | rs11789777 | 6.70E-04 |
| 9 | *MIR548Q* | 57 | 109653504 | 109848716 | 159.12 | 5.10E-03 | rs784658 | 8.10E-04 |
| 9 | *PRDM12* | 12 | 133539980 | 133558384 | 49.35 | 5.30E-03 | rs73551833 | 7.40E-04 |
| 2 | *TYW5* | 9 | 200793633 | 200820459 | 30.67 | 5.30E-03 | rs10497844 | 7.50E-04 |
| 6 | *GMPR* | 31 | 16238810 | 16295780 | 97.81 | 5.80E-03 | rs1042391 | 8.50E-04 |
| 13 | *ATP11AUN* | 39 | 113301357 | 113338811 | 135.85 | 6.20E-03 | rs4907738 | 1.70E-04 |
| 2 | *FAM168B* | 6 | 131805448 | 131851004 | 27.29 | 6.50E-03 | rs2313160 | 5.50E-04 |
| 3 | *DCLK3* | 14 | 36753912 | 36781352 | 53.54 | 6.70E-03 | rs74567016 | 5.60E-04 |
| 12 | *APOLD1* | 52 | 12878850 | 12944399 | 126.34 | 6.80E-03 | rs16908422 | 1.10E-04 |
| 18 | *LINC00907* | 187 | 39766632 | 40271389 | 452.74 | 7.10E-03 | rs7239883 | 1.60E-04 |
| 11 | *SPON1* | 130 | 13984183 | 14289679 | 336.57 | 7.30E-03 | rs10832228 | 2.60E-04 |
| 16 | *PGP* | 3 | 2261602 | 2264822 | 19.83 | 7.40E-03 | rs26848 | 4.10E-04 |
| 17 | *LOC101927688* | 16 | 56597197 | 56634286 | 67.13 | 7.40E-03 | rs9889631 | 9.40E-04 |
| 14 | *TTC7B* | 169 | 91006931 | 91282761 | 375.41 | 7.70E-03 | rs1286335 | 2.20E-04 |
| 16 | *SEC14L5* | 57 | 5008317 | 5069156 | 193.66 | 8.10E-03 | rs7198733 | 5.10E-04 |
| 5 | *FSTL4* | 281 | 132532151 | 132948223 | 501.53 | 8.50E-03 | rs10077572 | 7.50E-04 |
| 5 | *ADAMTS16* | 228 | 5140442 | 5320412 | 438.39 | 8.70E-03 | rs67272384 | 4.10E-04 |
| 2 | *NXPH2* | 52 | 139426726 | 139537811 | 143.93 | 8.90E-03 | rs11680550 | 1.60E-04 |
| 2 | *TTC7A* | 140 | 47143267 | 47303275 | 369.12 | 9.00E-03 | rs13411760 | 1.80E-04 |
| 2 | *DHX57* | 18 | 39024875 | 39103021 | 79.22 | 9.60E-03 | rs3099943 | 1.30E-04 |
| 12 | *TAPBPL* | 11 | 6561176 | 6571488 | 45.54 | 9.70E-03 | rs2532500 | 2.90E-04 |
| 17 | *RAB37* | 36 | 72667255 | 72743474 | 97.74 | 9.80E-03 | rs35489971 | 4.20E-04 |
| 10 | *LOC101926924* | 11 | 91675245 | 91717130 | 55.8 | 1.00E-02 | rs7903627 | 4.70E-05 |
| 12 | *KLRF2* | 8 | 10034087 | 10048432 | 34.03 | 1.00E-02 | rs618819 | 9.70E-04 |
| 13 | *LHFP* | 201 | 39917028 | 40177356 | 392.2 | 1.00E-02 | rs9566425 | 2.40E-04 |
| 12 | *VAMP1* | 14 | 6571403 | 6579843 | 58.7 | 1.10E-02 | rs1045452 | 4.00E-04 |
| 7 | *THSD7A* | 386 | 11410061 | 11871824 | 691.33 | 1.10E-02 | rs47 | 3.10E-04 |
| 12 | *MFAP5* | 10 | 8798539 | 8815433 | 35.51 | 1.20E-02 | rs35742897 | 5.40E-04 |
| 15 | *CHRNA7* | 52 | 32322685 | 32462384 | 146.86 | 1.20E-02 | rs2133965 | 7.80E-04 |
| 17 | *TMEM132E* | 53 | 32907767 | 32966337 | 117.31 | 1.20E-02 | rs10445404 | 1.10E-04 |
| 3 | *C3orf70* | 40 | 184795837 | 184870802 | 110.17 | 1.20E-02 | rs4687083 | 6.60E-04 |
| 2 | *ARHGEF4* | 56 | 131674223 | 131804826 | 141.18 | 1.20E-02 | rs10169932 | 8.10E-04 |
| 1 | *LOC100505768* | 18 | 87819209 | 87837338 | 43.06 | 1.20E-02 | rs7550556 | 5.20E-04 |
| 1 | *ADSS* | 17 | 244571793 | 244615436 | 56.7 | 1.20E-02 | rs12032765 | 6.10E-04 |
| 9 | *LOC101927502* | 54 | 84304627 | 84391814 | 153.17 | 1.30E-02 | rs62576233 | 7.50E-04 |
| 2 | *LINC01237* | 110 | 242823513 | 243020873 | 281.56 | 1.30E-02 | rs3934982 | 8.50E-04 |
| 16 | *C16orf45* | 32 | 15528324 | 15682116 | 96 | 1.40E-02 | rs62036867 | 3.70E-04 |
| 1 | *LOC400794* | 61 | 165446078 | 165551341 | 180.53 | 1.50E-02 | rs2433137 | 2.90E-04 |
| 7 | *VWC2* | 50 | 49813256 | 49952138 | 126.68 | 1.50E-02 | rs17664027 | 6.00E-04 |
| 1 | *SKI* | 52 | 2160133 | 2241652 | 121.7 | 1.50E-02 | rs2279702 | 2.20E-04 |
| 19 | *MYH14* | 84 | 50706884 | 50813801 | 164.8 | 1.60E-02 | rs74612541 | 4.90E-05 |
| 3 | *LOC101928135* | 197 | 34917288 | 35435515 | 411.12 | 1.60E-02 | rs2060661 | 9.10E-04 |
| 7 | *PDE1C* | 357 | 31790792 | 32339016 | 631.7 | 1.60E-02 | rs1117825 | 3.70E-04 |
| 10 | *SORBS1* | 177 | 97071529 | 97321177 | 386.5 | 1.70E-02 | rs1410602 | 3.30E-04 |
| 13 | *LINC00284* | 10 | 44596470 | 44604599 | 38.75 | 1.70E-02 | rs74064708 | 6.30E-04 |
| 14 | *RDH12* | 15 | 68168602 | 68201168 | 43.33 | 1.70E-02 | rs756473 | 9.30E-04 |
| 20 | *ZNF133* | 24 | 18268926 | 18297640 | 70.61 | 1.70E-02 | rs62205465 | 1.90E-04 |
| 12 | *TRPV4* | 23 | 110220891 | 110271212 | 78.7 | 1.80E-02 | rs4565962 | 1.50E-04 |
| 9 | *COL5A1* | 190 | 137533650 | 137736688 | 365.87 | 1.80E-02 | rs28391608 | 8.90E-04 |
| 2 | *CSRNP3* | 92 | 166326156 | 166545917 | 220.93 | 1.80E-02 | rs1007732 | 4.40E-04 |
| 9 | *PALM2-AKAP2* | 450 | 112542576 | 112934791 | 812.15 | 1.80E-02 | rs76540104 | 2.00E-04 |
| 17 | *BZRAP1* | 27 | 56378587 | 56406152 | 78.5 | 1.90E-02 | rs2526378 | 3.90E-04 |
| 3 | *FGF12* | 377 | 191857181 | 192445388 | 578.33 | 1.90E-02 | rs6784434 | 1.20E-04 |
| 5 | *ATG10* | 36 | 81267843 | 81551216 | 112.18 | 1.90E-02 | rs6870406 | 6.80E-04 |
| 1 | *ZYG11A* | 14 | 53308182 | 53360247 | 44.38 | 1.90E-02 | rs537822 | 6.90E-04 |
| 4 | *STOX2* | 80 | 184826508 | 184938875 | 156.59 | 1.90E-02 | rs12644266 | 8.20E-05 |
| 4 | *C4orf45* | 62 | 159814683 | 159956333 | 167.66 | 1.90E-02 | rs7693264 | 2.50E-04 |
| 19 | *GNA11* | 28 | 3094407 | 3124000 | 79.52 | 2.00E-02 | rs74389426 | 8.80E-04 |
| 5 | *ADAMTS12* | 281 | 33527286 | 33892124 | 488.23 | 2.00E-02 | rs12522957 | 6.10E-04 |
| 8 | *LOXL2* | 128 | 23154409 | 23261722 | 264.55 | 2.10E-02 | rs3808521 | 2.30E-04 |
| 2 | *RTP5* | 4 | 242811885 | 242815482 | 13.17 | 2.20E-02 | rs74000570 | 8.90E-04 |
| 2 | *LCLAT1* | 66 | 30670122 | 30867091 | 174.5 | 2.20E-02 | rs71444465 | 5.10E-04 |
| 10 | *TBATA* | 32 | 72530994 | 72545157 | 80.96 | 2.30E-02 | rs2254433 | 4.50E-04 |
| 15 | *TRPM1* | 107 | 31293263 | 31453476 | 269.19 | 2.30E-02 | rs28451564 | 4.20E-04 |
| 23 | *PTCHD1-AS* | 308 | 22277913 | 23311263 | 477.57 | 2.30E-02 | rs5951567 | 1.70E-04 |
| 3 | *ERICH6* | 32 | 150377674 | 150421742 | 78.78 | 2.40E-02 | rs72616691 | 7.20E-04 |
| 13 | *ZC3H13* | 21 | 46536313 | 46626896 | 56.23 | 2.50E-02 | rs78114150 | 3.50E-04 |
| 23 | *ELF4* | 5 | 129198894 | 129244688 | 17.55 | 2.50E-02 | rs3788848 | 7.60E-04 |
| 1 | *SPSB1* | 80 | 9352940 | 9429590 | 181.29 | 2.50E-02 | rs9435207 | 8.90E-04 |
| 11 | *ARHGAP32* | 44 | 128834954 | 129062093 | 107.38 | 2.70E-02 | rs2439805 | 6.40E-04 |
| 1 | *RABGAP1L_1* | 159 | 174128551 | 174964445 | 459.83 | 2.70E-02 | rs12565430 | 3.00E-04 |
| 4 | *AMTN* | 7 | 71384288 | 71398460 | 20.9 | 2.80E-02 | rs35286445 | 5.70E-04 |
| 2 | *TBC1D8* | 77 | 101623689 | 101767846 | 172.81 | 2.80E-02 | rs6543017 | 2.30E-04 |
| 9 | *GCNT1* | 46 | 79056581 | 79122332 | 106.62 | 2.80E-02 | rs56166450 | 1.90E-04 |
| 15 | *DET1* | 9 | 89055713 | 89089912 | 28.67 | 2.90E-02 | rs13835 | 4.30E-04 |
| 8 | *LINC01111* | 45 | 77318888 | 77436567 | 100.91 | 2.90E-02 | rs13281210 | 8.60E-05 |
| 14 | *CHD8* | 7 | 21853352 | 21905457 | 22.49 | 3.10E-02 | rs77545594 | 4.90E-04 |
| 7 | *HIP1* | 120 | 75162618 | 75368290 | 216.63 | 3.40E-02 | rs1179640 | 8.20E-04 |
| 1 | *NCMAP* | 50 | 24882566 | 24935818 | 114.7 | 3.50E-02 | rs9728914 | 4.20E-04 |
| 4 | *MUC7* | 16 | 71296208 | 71348714 | 42.37 | 3.50E-02 | rs3733492 | 4.10E-04 |
| 2 | *RMDN2* | 90 | 38152461 | 38294285 | 196.92 | 3.60E-02 | rs336036 | 3.90E-04 |
| 10 | *PPAPDC1A* | 101 | 122216465 | 122349367 | 183.64 | 3.70E-02 | rs2901245 | 8.60E-04 |
| 17 | *LOC100507351* | 30 | 75543022 | 75561103 | 68.05 | 3.70E-02 | rs7226077 | 7.30E-04 |
| 6 | *DNAH8* | 214 | 38683116 | 38998574 | 385.86 | 3.70E-02 | rs6919590 | 6.80E-04 |
| 1 | *MAGOH* | 7 | 53692563 | 53704282 | 20.25 | 3.70E-02 | rs13375749 | 7.30E-04 |
| 7 | *LOC349160* | 99 | 136583519 | 136849088 | 198.06 | 3.90E-02 | rs17506977 | 1.40E-04 |
| 1 | *KCNH1* | 216 | 210851656 | 211307457 | 388.75 | 3.90E-02 | rs4951487 | 1.30E-04 |
| 12 | *TMTC1* | 233 | 29653745 | 29937692 | 379.39 | 4.00E-02 | rs34082635 | 2.60E-04 |
| 6 | *PHACTR1* | 232 | 12717036 | 13288075 | 391.31 | 4.00E-02 | rs445591 | 7.10E-04 |
| 10 | *IL2RA* | 58 | 6052656 | 6104333 | 117.83 | 4.10E-02 | rs12722602 | 3.50E-04 |
| 8 | *C8orf37-AS1* | 359 | 96281063 | 96822371 | 548.03 | 4.10E-02 | rs16917906 | 4.40E-04 |
| 4 | *RPL34-AS1* | 27 | 109459345 | 109541613 | 72.16 | 4.10E-02 | rs6854331 | 2.80E-04 |
| 3 | *SCHIP1* | 217 | 158991035 | 159615155 | 356.23 | 4.10E-02 | rs78601513 | 8.30E-04 |
| 14 | *SLC7A8* | 47 | 23594503 | 23652869 | 94.07 | 4.20E-02 | rs999165 | 1.30E-04 |
| 17 | *PRKAR1A* | 88 | 66409763 | 66547457 | 179.15 | 4.30E-02 | rs10852738 | 9.10E-04 |
| 10 | *PIK3AP1* | 72 | 98353068 | 98480279 | 144.97 | 4.40E-02 | rs880348 | 8.50E-04 |
| 10 | *TCERG1L* | 258 | 132890654 | 133109984 | 406.22 | 4.50E-02 | rs2918108 | 1.20E-04 |
| 1 | *TRAF3IP3* | 27 | 209929376 | 209955668 | 56.88 | 4.50E-02 | rs17015183 | 9.20E-04 |
| 5 | *PPARGC1B* | 103 | 149109814 | 149234585 | 196.04 | 4.50E-02 | rs73267734 | 3.90E-04 |
| 2 | *TRPM8* | 102 | 234826042 | 234928166 | 171.66 | 4.60E-02 | rs2362295 | 3.30E-04 |
| 13 | *CENPJ* | 32 | 25456411 | 25497027 | 65.61 | 4.70E-02 | rs1530876 | 9.90E-04 |
| 19 | *NFIC* | 68 | 3359560 | 3469215 | 125.84 | 4.80E-02 | rs74967471 | 1.20E-04 |
| 9 | *COL15A1* | 111 | 101705994 | 101833074 | 200.2 | 4.80E-02 | rs2050257 | 7.50E-04 |
| 15 | *ACAN* | 92 | 89346673 | 89418585 | 146.37 | 4.90E-02 | rs8023285 | 7.90E-04 |
| 16 | *GALNS* | 41 | 88880141 | 88923374 | 94.1 | 4.90E-02 | rs12935469 | 4.70E-04 |
| 5 | *SERINC5* | 92 | 79407049 | 79551898 | 169.84 | 4.90E-02 | rs35085860 | 2.90E-04 |
| 5 | *GALNT10* | 158 | 153570294 | 153800543 | 266.04 | 4.90E-02 | rs12520590 | 7.60E-04 |

**Supplementary Table S3-5**. The genes nominally associated with BEHLs at the frequency of 8.0 kHz from VEGAS2 gene-based analysis (*P* < 0.05)

| Chr | Gene | nSNPs | Start position | Stop position | Gene-based test statistic | P-value | Top-SNP | Top-SNP P-value |
| --- | --- | --- | --- | --- | --- | --- | --- | --- |
| 6 | *FAM184A* | 67 | 119280993 | 119470358 | 454.4 | 5.70E-05 | rs7745460 | 7.80E-06 |
| 5 | *NR3C1* | 78 | 142657495 | 142815077 | 478.96 | 6.00E-05 | rs13306588 | 1.60E-04 |
| 3 | *OTOL1* | 4 | 161214595 | 161221730 | 30.55 | 7.50E-05 | rs3921595 | 6.80E-04 |
| 3 | *LPP-AS1* | 4 | 188280024 | 188286454 | 41.65 | 1.10E-04 | rs13092374 | 1.00E-04 |
| 6 | *SLC22A7* | 5 | 43265997 | 43273276 | 45.34 | 1.50E-04 | rs2270860 | 4.40E-04 |
| 3 | *PSMD6-AS2* | 5 | 63989697 | 63997917 | 48.45 | 1.70E-04 | rs1046025 | 3.00E-04 |
| 4 | *KLHL2* | 16 | 166128769 | 166244308 | 120.74 | 1.80E-04 | rs11726531 | 1.50E-04 |
| 23 | *AMMECR1* | 2 | 109437413 | 109683461 | 28.19 | 1.90E-04 | rs3788769 | 1.70E-04 |
| 22 | *LOC101929664* | 20 | 30404730 | 30476469 | 111.77 | 2.10E-04 | rs41162 | 4.90E-04 |
| 17 | *CD300LD* | 8 | 72576110 | 72588370 | 47.78 | 2.80E-04 | rs1699585 | 6.70E-04 |
| 1 | *SLC35E2B* | 15 | 1592938 | 1624243 | 59.39 | 3.10E-04 | rs4074196 | 2.00E-03 |
| 4 | *LEF1* | 28 | 108968700 | 109090112 | 187.33 | 3.40E-04 | rs17038688 | 4.20E-05 |
| 6 | *NCOA7* | 51 | 126102306 | 126253176 | 265.06 | 3.60E-04 | rs584032 | 4.90E-05 |
| 10 | *PPAPDC1A* | 101 | 122216465 | 122349367 | 316.58 | 4.10E-04 | rs2901245 | 2.80E-04 |
| 14 | *BCL11B* | 96 | 99635624 | 99738050 | 329.31 | 4.10E-04 | rs2793321 | 3.00E-05 |
| 1 | *CDK11B* | 16 | 1570602 | 1655859 | 64.43 | 4.10E-04 | rs4074196 | 2.00E-03 |
| 5 | *G3BP1* | 11 | 151151475 | 151184915 | 68.53 | 4.40E-04 | rs10477000 | 3.80E-04 |
| 2 | *TBC1D8* | 77 | 101623689 | 101767846 | 311.3 | 5.10E-04 | rs2309938 | 1.00E-06 |
| 3 | *EAF2* | 17 | 121554033 | 121605373 | 106.47 | 5.30E-04 | rs10511407 | 5.30E-04 |
| 7 | *TAS2R4* | 6 | 141478288 | 141479188 | 36.19 | 5.50E-04 | rs2233998 | 1.10E-03 |
| 20 | *OGFR-AS1* | 2 | 61431978 | 61436939 | 14.86 | 5.70E-04 | rs6090117 | 3.20E-04 |
| 17 | *C17orf77* | 11 | 72581056 | 72590348 | 56.74 | 5.80E-04 | rs524536 | 4.80E-04 |
| 11 | *RRP8* | 9 | 6621143 | 6624880 | 36.46 | 5.90E-04 | rs12420597 | 4.00E-03 |
| 12 | *LOH12CR1* | 54 | 12510019 | 12619838 | 253.24 | 6.30E-04 | rs9645752 | 1.60E-05 |
| 17 | *MYO18A* | 31 | 27400527 | 27507407 | 174.4 | 6.70E-04 | rs11080090 | 3.70E-06 |
| 3 | *PSMD6* | 7 | 63996224 | 64009686 | 45 | 6.70E-04 | rs1046025 | 3.00E-04 |
| 6 | *ZNF318* | 12 | 43303807 | 43337181 | 70.26 | 7.20E-04 | rs7692 | 2.30E-04 |
| 23 | *IL1RAPL1* | 151 | 28605680 | 29974017 | 393.1 | 7.60E-04 | rs1015927 | 9.60E-05 |
| 1 | *NCMAP* | 50 | 24882566 | 24935818 | 211.36 | 7.60E-04 | rs12354378 | 2.00E-05 |
| 5 | *SREK1* | 10 | 65440045 | 65479444 | 54.18 | 8.00E-04 | rs27079 | 6.90E-04 |
| 5 | *PPARGC1B* | 103 | 149109814 | 149234585 | 343.35 | 9.70E-04 | rs32581 | 1.80E-04 |
| 19 | *DLL3* | 7 | 39989556 | 39999121 | 60.84 | 1.00E-03 | rs3212276 | 3.30E-04 |
| 20 | *OGFR* | 10 | 61436176 | 61445352 | 50.25 | 1.00E-03 | rs7275041 | 3.10E-04 |
| 3 | *ATXN7* | 28 | 63850232 | 63989136 | 141.58 | 1.00E-03 | rs56165216 | 2.30E-04 |
| 12 | *GLIPR1L2* | 12 | 75784849 | 75826177 | 70.38 | 1.10E-03 | rs7300308 | 3.00E-04 |
| 22 | *MCAT* | 8 | 43528211 | 43539403 | 54.86 | 1.10E-03 | rs2072852 | 9.10E-04 |
| 3 | *C3orf49* | 7 | 63805040 | 63834312 | 41.05 | 1.10E-03 | rs2087704 | 3.20E-04 |
| 3 | *B3GALNT1* | 14 | 160801670 | 160823160 | 72.54 | 1.10E-03 | rs11717272 | 7.80E-04 |
| 1 | *TAF5L* | 12 | 229728865 | 229761794 | 69.15 | 1.20E-03 | rs7547607 | 4.10E-04 |
| 22 | *HORMAD2* | 23 | 30476452 | 30573062 | 131.78 | 1.30E-03 | rs718772 | 2.60E-06 |
| 1 | *LRIF1* | 7 | 111489811 | 111506566 | 38.24 | 1.30E-03 | rs2232041 | 5.20E-04 |
| 8 | *LRRC6* | 23 | 133584200 | 133687863 | 120.17 | 1.40E-03 | rs16904707 | 1.50E-04 |
| 19 | *NKG7* | 2 | 51874873 | 51875960 | 13 | 1.60E-03 | rs3009 | 3.20E-04 |
| 3 | *LPP* | 410 | 187871662 | 188608460 | 754.52 | 1.70E-03 | rs9283657 | 3.80E-05 |
| 7 | *PRPS1L1* | 2 | 18066399 | 18067486 | 13.11 | 1.70E-03 | rs3800961 | 5.90E-04 |
| 12 | *RILPL2* | 7 | 123899935 | 123921264 | 37.65 | 1.80E-03 | rs1707730 | 2.20E-05 |
| 9 | *GLDC* | 68 | 6532463 | 6645692 | 207.47 | 1.80E-03 | rs4512434 | 4.40E-05 |
| 6 | *PSORS1C2* | 22 | 31105310 | 31107127 | 59.62 | 1.80E-03 | rs2072108 | 9.70E-04 |
| 15 | *LDHAL6B* | 12 | 59499014 | 59500785 | 62.53 | 1.90E-03 | rs3809530 | 3.70E-04 |
| 6 | *ASF1A* | 6 | 119215240 | 119230335 | 33.55 | 1.90E-03 | rs9489536 | 8.30E-04 |
| 4 | *MAP9* | 24 | 156263811 | 156298122 | 99.08 | 1.90E-03 | rs41280487 | 2.30E-04 |
| 17 | *FAM20A* | 42 | 66531256 | 66597095 | 128.69 | 2.00E-03 | rs12603503 | 3.60E-05 |
| 19 | *COPE* | 5 | 19010322 | 19030199 | 30.29 | 2.00E-03 | rs1468460 | 5.80E-04 |
| 3 | *CLDN1* | 32 | 190023489 | 190040235 | 135.26 | 2.00E-03 | rs9869263 | 3.40E-04 |
| 2 | *CSRNP3* | 92 | 166326156 | 166545917 | 312.25 | 2.00E-03 | rs1007732 | 1.00E-04 |
| 11 | *IFITM10* | 6 | 1753639 | 1771824 | 40.42 | 2.10E-03 | rs3740621 | 1.70E-04 |
| 11 | *CTSD* | 11 | 1773981 | 1785222 | 60.24 | 2.10E-03 | rs55923455 | 3.10E-04 |
| 22 | *MTMR3* | 49 | 30279157 | 30426857 | 184.67 | 2.20E-03 | rs41162 | 4.90E-04 |
| 6 | *CDKAL1* | 330 | 20534687 | 21232634 | 867.47 | 2.20E-03 | rs59800273 | 6.10E-04 |
| 9 | *PTPRD-AS2* | 7 | 10613205 | 10620420 | 38.8 | 2.20E-03 | rs10959201 | 5.70E-04 |
| 3 | *GOLGB1* | 25 | 121382045 | 121468614 | 101.83 | 2.30E-03 | rs1919554 | 2.20E-04 |
| 18 | *DSG1* | 39 | 28898051 | 28937393 | 137.37 | 2.40E-03 | rs16961689 | 8.90E-04 |
| 11 | *GUCY1A2* | 143 | 106544737 | 106889171 | 425.15 | 2.50E-03 | rs1455588 | 2.00E-04 |
| 11 | *LOC100132078* | 5 | 111284966 | 111288911 | 28.92 | 2.50E-03 | rs1944927 | 6.30E-04 |
| 17 | *NOL11* | 10 | 65714060 | 65740266 | 46.91 | 2.50E-03 | rs12601677 | 8.20E-05 |
| 7 | *TMEM196* | 34 | 19758937 | 19812404 | 167 | 2.50E-03 | rs7791038 | 7.80E-04 |
| 6 | *MCM9* | 21 | 119134611 | 119256327 | 103.78 | 2.60E-03 | rs12197931 | 8.30E-04 |
| 2 | *XRCC5* | 39 | 216974019 | 217071016 | 166.86 | 2.60E-03 | rs207906 | 1.10E-04 |
| 10 | *BAG3* | 27 | 121410881 | 121437329 | 114.32 | 2.90E-03 | rs196336 | 9.40E-04 |
| 7 | *PTPN12* | 26 | 77166772 | 77269388 | 110.95 | 3.00E-03 | rs2302474 | 7.90E-04 |
| 17 | *RAB37* | 36 | 72667255 | 72743474 | 114.05 | 3.30E-03 | rs35489971 | 1.20E-04 |
| 2 | *DNPEP* | 11 | 220238179 | 220252662 | 59.43 | 3.30E-03 | rs4672926 | 4.10E-04 |
| 9 | *TTC39B* | 109 | 15170841 | 15307358 | 286.02 | 3.30E-03 | rs4008004 | 3.50E-04 |
| 22 | *NFAM1* | 47 | 42776413 | 42828401 | 220.72 | 3.40E-03 | rs2072863 | 3.00E-04 |
| 10 | *FAM160B1* | 29 | 116581502 | 116659586 | 119.61 | 3.50E-03 | rs10885625 | 8.20E-04 |
| 17 | *CD300LF* | 15 | 72690446 | 72709139 | 55.02 | 3.50E-03 | rs35489971 | 1.20E-04 |
| 12 | *GLTP* | 11 | 110288747 | 110318293 | 57.72 | 3.70E-03 | rs10850913 | 5.00E-04 |
| 23 | *GABRA3* | 33 | 151335633 | 151619831 | 113.16 | 3.80E-03 | rs11796556 | 4.40E-04 |
| 18 | *LAMA3* | 67 | 21269561 | 21535029 | 261.72 | 4.10E-03 | rs12185288 | 1.70E-04 |
| 8 | *JPH1* | 46 | 75146938 | 75233562 | 155.79 | 4.10E-03 | rs12334848 | 9.40E-04 |
| 7 | *C7orf63* | 21 | 89874487 | 89940377 | 89.38 | 4.30E-03 | rs916648 | 7.40E-04 |
| 1 | *KIAA1804* | 50 | 233463513 | 233520894 | 227.75 | 4.40E-03 | rs4649220 | 5.30E-04 |
| 20 | *MMP9* | 13 | 44637546 | 44645200 | 43.67 | 4.50E-03 | rs3918254 | 7.80E-04 |
| 17 | *GIT1* | 7 | 27900486 | 27916610 | 30.74 | 4.70E-03 | rs563976 | 1.50E-04 |
| 5 | *CCNJL* | 44 | 159678670 | 159739573 | 154.81 | 4.70E-03 | rs12658758 | 1.50E-04 |
| 1 | *CD34* | 21 | 208059882 | 208084683 | 80.53 | 4.70E-03 | rs2745952 | 1.20E-04 |
| 6 | *PDE10A* | 246 | 165740775 | 166075588 | 509.51 | 4.90E-03 | rs75796379 | 5.50E-04 |
| 11 | *TTC17* | 30 | 43380434 | 43516483 | 100.05 | 5.00E-03 | rs79494239 | 2.90E-04 |
| 13 | *GPC6* | 625 | 93879077 | 95060273 | 1075.87 | 5.20E-03 | rs1933779 | 3.10E-04 |
| 13 | *NUFIP1* | 11 | 45513383 | 45563613 | 50.19 | 5.30E-03 | rs3783151 | 2.60E-04 |
| 19 | *TCF3* | 17 | 1609288 | 1652328 | 63.42 | 5.40E-03 | rs740436 | 3.00E-04 |
| 19 | *SUPT5H* | 12 | 39936185 | 39967308 | 48.42 | 5.40E-03 | rs12985456 | 9.30E-04 |
| 4 | *MIR548AJ2* | 129 | 23199716 | 23464726 | 328.96 | 5.40E-03 | rs59183220 | 6.50E-04 |
| 3 | *CHRD* | 9 | 184097860 | 184107617 | 40.16 | 5.60E-03 | rs885650 | 3.60E-04 |
| 7 | *DAGLB* | 27 | 6448746 | 6487643 | 94.02 | 5.70E-03 | rs836540 | 5.00E-04 |
| 6 | *PTCHD4* | 65 | 47845763 | 48036425 | 202.64 | 6.20E-03 | rs332591 | 6.30E-04 |
| 4 | *GPR125* | 101 | 22388996 | 22517677 | 306.32 | 6.40E-03 | rs6818597 | 1.60E-04 |
| 3 | *C3orf70* | 40 | 184795837 | 184870802 | 120.56 | 6.50E-03 | rs2132240 | 4.50E-04 |
| 17 | *ENO3* | 3 | 4854383 | 4860426 | 13.88 | 6.60E-03 | rs143945974 | 2.20E-04 |
| 18 | *CPLX4* | 23 | 56962635 | 56985881 | 83.37 | 6.60E-03 | rs609209 | 3.00E-04 |
| 3 | *THOC7* | 8 | 63819545 | 63849599 | 32.34 | 7.20E-03 | rs2087704 | 3.20E-04 |
| 16 | *FAM92B* | 18 | 85131964 | 85146114 | 61.86 | 7.30E-03 | rs16975240 | 9.40E-04 |
| 7 | *LOC401324* | 53 | 35353465 | 35416086 | 149.82 | 7.40E-03 | rs9969168 | 2.70E-04 |
| 17 | *CTNS* | 29 | 3539761 | 3566397 | 116.76 | 7.70E-03 | rs1800528 | 7.60E-04 |
| 14 | *CLMN* | 94 | 95648275 | 95786245 | 246.6 | 8.20E-03 | rs7158880 | 6.50E-04 |
| 1 | *PIK3CD* | 31 | 9711789 | 9789172 | 94.06 | 8.80E-03 | rs12039250 | 8.80E-04 |
| 20 | *COL9A3* | 21 | 61448413 | 61472511 | 57.69 | 9.10E-03 | rs34689750 | 6.60E-05 |
| 10 | *IL2RA* | 58 | 6052656 | 6104333 | 153.8 | 9.30E-03 | rs12722602 | 5.20E-04 |
| 6 | *PARK2* | 944 | 161768589 | 163148834 | 1426.78 | 9.40E-03 | rs2096982 | 5.40E-07 |
| 8 | *MATN2* | 110 | 98881310 | 99048946 | 275.58 | 9.80E-03 | rs11559201 | 6.40E-04 |
| 13 | *PSPC1* | 17 | 20248891 | 20357159 | 61.26 | 1.00E-02 | rs3742148 | 9.20E-04 |
| 14 | *LINC00648* | 10 | 48234155 | 48264217 | 36.05 | 1.00E-02 | rs74664365 | 2.40E-04 |
| 17 | *MYO1C* | 24 | 1367479 | 1396001 | 68.69 | 1.10E-02 | rs11654497 | 5.50E-04 |
| 7 | *IL6* | 5 | 22766765 | 22771621 | 18.95 | 1.10E-02 | rs2069840 | 5.60E-04 |
| 4 | *PRDM5* | 98 | 121615928 | 121844013 | 279.64 | 1.10E-02 | rs10857072 | 2.80E-04 |
| 9 | *PIP5K1B* | 178 | 71320329 | 71624092 | 442.96 | 1.20E-02 | rs77317374 | 3.30E-05 |
| 1 | *RASAL2* | 94 | 178062863 | 178448648 | 325.3 | 1.20E-02 | rs12143791 | 6.50E-06 |
| 16 | *CMIP* | 308 | 81478774 | 81745367 | 548.42 | 1.30E-02 | rs60963566 | 5.00E-04 |
| 9 | *PAPPA* | 123 | 118916070 | 119164600 | 235.87 | 1.30E-02 | rs620395 | 3.60E-04 |
| 8 | *MIR548O2* | 58 | 29920257 | 30108213 | 187.08 | 1.30E-02 | rs1995947 | 1.50E-04 |
| 3 | *EIF2A* | 13 | 150264573 | 150303803 | 48.61 | 1.30E-02 | rs12629958 | 8.30E-04 |
| 5 | *BRD9* | 8 | 863849 | 892939 | 33.52 | 1.30E-02 | rs6555556 | 9.20E-04 |
| 2 | *LYPD6* | 59 | 150186498 | 150330659 | 172.47 | 1.30E-02 | rs1196683 | 3.10E-04 |
| 17 | *MGAT5B* | 144 | 74864797 | 74946471 | 280.11 | 1.40E-02 | rs571264 | 7.50E-04 |
| 15 | *MYO1E* | 156 | 59428167 | 59665071 | 309.86 | 1.60E-02 | rs3809530 | 3.70E-04 |
| 16 | *TOX3* | 46 | 52471917 | 52581714 | 108.42 | 1.60E-02 | rs9935437 | 1.20E-04 |
| 16 | *MAP1LC3B* | 4 | 87425800 | 87438380 | 16.57 | 1.70E-02 | rs8044820 | 3.90E-04 |
| 8 | *FDFT1* | 38 | 11653081 | 11696818 | 103.77 | 1.70E-02 | rs3779663 | 6.90E-04 |
| 10 | *BTBD16* | 49 | 124030820 | 124097676 | 123.37 | 1.80E-02 | rs10887135 | 6.10E-05 |
| 7 | *LHFPL3* | 328 | 103969103 | 104549003 | 580.44 | 1.80E-02 | rs9648932 | 2.70E-04 |
| 6 | *IFNGR1* | 9 | 137518620 | 137540567 | 25.92 | 1.80E-02 | rs3799488 | 9.60E-04 |
| 22 | *NF2* | 32 | 29999544 | 30094589 | 97.52 | 1.90E-02 | rs756053 | 7.10E-04 |
| 8 | *LEPROTL1* | 18 | 29952921 | 29995222 | 58.78 | 2.00E-02 | rs1995947 | 1.50E-04 |
| 10 | *H2AFY2* | 40 | 71812356 | 71872040 | 99.82 | 2.10E-02 | rs10999124 | 6.60E-04 |
| 8 | *ZFHX4* | 65 | 77593514 | 77779521 | 148.94 | 2.10E-02 | rs17363324 | 2.60E-04 |
| 6 | *GFOD1* | 58 | 13363586 | 13487869 | 135.75 | 2.10E-02 | rs9357728 | 3.90E-04 |
| 2 | *LOC101927619* | 27 | 199164086 | 199239821 | 78.4 | 2.10E-02 | rs12996222 | 6.80E-04 |
| 1 | *SRRM1* | 9 | 24969593 | 24999772 | 36.82 | 2.10E-02 | rs7546441 | 1.10E-04 |
| 3 | *PPM1L* | 95 | 160473995 | 160788817 | 244.99 | 2.20E-02 | rs1580254 | 1.00E-03 |
| 1 | *EPHA8* | 35 | 22890003 | 22930087 | 95.75 | 2.50E-02 | rs79717304 | 7.00E-04 |
| 2 | *KYNU* | 67 | 143635194 | 143799885 | 155.26 | 2.60E-02 | rs4609990 | 7.30E-04 |
| 14 | *PPP1R13B* | 25 | 104200087 | 104313927 | 70.1 | 2.70E-02 | rs12436676 | 6.10E-04 |
| 8 | *LINC01111* | 45 | 77318888 | 77436567 | 104.41 | 2.70E-02 | rs13281210 | 3.50E-04 |
| 13 | *FRY* | 151 | 32605436 | 32870776 | 268.22 | 2.80E-02 | rs456705 | 2.80E-05 |
| 14 | *VRTN* | 7 | 74815165 | 74826711 | 19.46 | 2.80E-02 | rs2232035 | 4.80E-04 |
| 6 | *DNAH8* | 214 | 38683116 | 38998574 | 401.9 | 2.80E-02 | rs6927413 | 6.30E-04 |
| 20 | *JAG1* | 39 | 10618331 | 10654694 | 84.22 | 2.90E-02 | rs6074164 | 2.90E-06 |
| 6 | *ARHGAP18* | 106 | 129898239 | 130031370 | 233.38 | 2.90E-02 | rs763132 | 5.60E-04 |
| 1 | *LDLRAP1* | 16 | 25870075 | 25895377 | 48.24 | 2.90E-02 | rs35432405 | 7.50E-04 |
| 9 | *IL33* | 22 | 6215785 | 6257983 | 85.17 | 2.90E-02 | rs76864631 | 2.90E-04 |
| 22 | *THOC5* | 27 | 29904155 | 29949736 | 72.93 | 3.00E-02 | rs1049536 | 2.80E-04 |
| 3 | *ERC2* | 470 | 55542335 | 56502391 | 757.17 | 3.00E-02 | rs11920153 | 1.00E-05 |
| 1 | *KCND3* | 174 | 112318453 | 112531777 | 321.59 | 3.20E-02 | rs17028939 | 9.60E-04 |
| 17 | *HS3ST3B1* | 57 | 14204505 | 14249492 | 103.4 | 3.50E-02 | rs79974538 | 1.80E-04 |
| 16 | *CLEC16A* | 107 | 11038344 | 11276046 | 246.7 | 3.60E-02 | rs78455166 | 7.00E-05 |
| 15 | *LOC283710* | 9 | 31514970 | 31523050 | 23.24 | 3.70E-02 | rs3743235 | 8.20E-04 |
| 15 | *KLF13* | 26 | 31619082 | 31670102 | 58.28 | 3.90E-02 | rs66501706 | 3.80E-04 |
| 17 | *SLC39A11* | 394 | 70642084 | 71088853 | 563.76 | 3.90E-02 | rs60898162 | 7.10E-04 |
| 6 | *AKAP12* | 112 | 151561133 | 151679694 | 199.08 | 3.90E-02 | rs2294793 | 8.00E-05 |
| 7 | *HDAC9* | 514 | 18126571 | 19036992 | 758.53 | 4.00E-02 | rs17419156 | 1.70E-05 |
| 3 | *IQCB1* | 14 | 121488609 | 121553926 | 37.98 | 4.00E-02 | rs12492861 | 5.30E-04 |
| 2 | *METTL8* | 30 | 172173912 | 172291312 | 65.56 | 4.00E-02 | rs77436825 | 6.50E-04 |
| 1 | *LOC100505768* | 18 | 87819209 | 87837338 | 35.32 | 4.10E-02 | rs7550556 | 8.90E-04 |
| 2 | *SERPINE2* | 62 | 224839764 | 224904036 | 136.51 | 4.10E-02 | rs4674854 | 5.30E-04 |
| 4 | *LOC101927282* | 27 | 130645325 | 130692633 | 64.58 | 4.20E-02 | rs2637592 | 5.60E-04 |
| 12 | *LTA4H* | 26 | 96394530 | 96437298 | 67.82 | 4.30E-02 | rs2247304 | 4.40E-06 |
| 9 | *GRIN3A* | 95 | 104331633 | 104500862 | 203.42 | 4.30E-02 | rs4743477 | 3.00E-04 |
| 5 | *FAM196B* | 84 | 169290718 | 169407744 | 152.02 | 4.50E-02 | rs1363576 | 8.30E-04 |
| 9 | *LINC00587* | 49 | 105281918 | 105419791 | 125.59 | 4.90E-02 | rs4743564 | 5.70E-04 |
| 4 | *MAML3* | 260 | 140637545 | 141075233 | 389.14 | 4.90E-02 | rs10002458 | 3.50E-04 |

**Supplementary Table S3-6**. The genes nominally associated with PTA from VEGAS2 gene-based analysis (*P* < 0.05)

| **Chr** | **Gene** | **nSNPs** | **Start position** | **Stop position** | **Gene-based test statistic** | **P-value** | **Top-SNP** | **Top-SNP P-value** |
| --- | --- | --- | --- | --- | --- | --- | --- | --- |
| 11 | *RRP8* | 9 | 6621143 | 6624880 | 55.09 | 9.00E-06 | rs17834692 | 4.20E-04 |
| 12 | *GLTP* | 11 | 110288747 | 110318293 | 106.95 | 2.30E-05 | rs10850913 | 2.30E-06 |
| 1 | *ADAMTS4* | 9 | 161159537 | 161168845 | 56.79 | 5.20E-05 | rs4233367 | 1.80E-04 |
| 12 | *GLIPR1L2* | 12 | 75784849 | 75826177 | 102.32 | 5.30E-05 | rs7300308 | 1.20E-05 |
| 23 | *PIR-FIGF* | 18 | 15363712 | 15509432 | 171.28 | 7.80E-05 | rs170905 | 4.90E-05 |
| 23 | *PIR* | 17 | 15402923 | 15511711 | 186.64 | 1.10E-04 | rs170905 | 4.90E-05 |
| 3 | *C3orf70* | 40 | 184795837 | 184870802 | 190.09 | 1.50E-04 | rs2132240 | 1.50E-05 |
| 4 | *KLHL2* | 16 | 166128769 | 166244308 | 123.2 | 1.50E-04 | rs117045966 | 3.80E-05 |
| 3 | *VPS8* | 49 | 184529930 | 184770402 | 308.62 | 1.70E-04 | rs2271257 | 1.60E-04 |
| 23 | *PIGA* | 5 | 15337572 | 15353676 | 66.6 | 2.20E-04 | rs3661 | 2.30E-04 |
| 22 | *MCAT* | 8 | 43528211 | 43539403 | 67.42 | 2.40E-04 | rs2072852 | 7.40E-04 |
| 3 | *OTOL1* | 4 | 161214595 | 161221730 | 26.02 | 2.60E-04 | rs3921595 | 2.50E-03 |
| 16 | *ZNF23* | 4 | 71481502 | 71496117 | 29.49 | 2.90E-04 | rs1609869 | 1.00E-03 |
| 19 | *PSG11* | 3 | 43511808 | 43530631 | 17.07 | 3.30E-04 | rs111493043 | 2.70E-03 |
| 7 | *PDE1C* | 357 | 31790792 | 32339016 | 862.51 | 4.40E-04 | rs6968484 | 7.90E-05 |
| 6 | *PSORS1C2* | 22 | 31105310 | 31107127 | 71.11 | 4.60E-04 | rs1265094 | 2.90E-03 |
| 20 | *POFUT1* | 4 | 30795695 | 30826467 | 18.55 | 4.80E-04 | rs79984831 | 4.70E-03 |
| 7 | *FAM131B* | 5 | 143050492 | 143059840 | 28.08 | 5.10E-04 | rs4236482 | 2.40E-03 |
| 1 | *RC3H1* | 9 | 173900351 | 173962210 | 69.02 | 5.20E-04 | rs9425780 | 2.10E-04 |
| 1 | *RHBG* | 21 | 156338979 | 156355013 | 97.68 | 6.30E-04 | rs11585523 | 3.00E-04 |
| 5 | *LINC00461* | 34 | 87836596 | 87980620 | 186.27 | 6.50E-04 | rs3814424 | 2.60E-04 |
| 10 | *SGPL1* | 21 | 72575703 | 72640932 | 100.82 | 7.50E-04 | rs10509327 | 5.10E-04 |
| 2 | *ATP6V1E2* | 7 | 46738985 | 46747096 | 35.19 | 8.20E-04 | rs4952830 | 2.30E-03 |
| 23 | *ASB11* | 4 | 15299830 | 15333746 | 34.8 | 8.70E-04 | rs5935944 | 2.30E-04 |
| 14 | *LINC00520* | 9 | 56247852 | 56263392 | 42.7 | 9.10E-04 | rs8008130 | 2.00E-04 |
| 14 | *LINC00637* | 2 | 104314057 | 104324386 | 14.37 | 1.00E-03 | rs7140323 | 5.50E-04 |
| 18 | *LAMA3* | 67 | 21269561 | 21535029 | 325.13 | 1.00E-03 | rs12185288 | 1.50E-04 |
| 5 | *NR3C1* | 78 | 142657495 | 142815077 | 340.66 | 1.00E-03 | rs33383 | 4.40E-04 |
| 1 | *MRPS14* | 7 | 174982093 | 174992591 | 41.08 | 1.00E-03 | rs16847706 | 1.80E-04 |
| 2 | *TBC1D8* | 77 | 101623689 | 101767846 | 281.1 | 1.10E-03 | rs2309938 | 9.60E-06 |
| 8 | *TNFRSF10B* | 40 | 22877647 | 22926700 | 144.98 | 1.20E-03 | rs7817787 | 2.70E-04 |
| 9 | *COL5A1* | 190 | 137533650 | 137736688 | 507.92 | 1.30E-03 | rs3811158 | 9.30E-05 |
| 10 | *CRTAC1* | 82 | 99624756 | 99790585 | 262.99 | 1.50E-03 | rs4919154 | 3.20E-04 |
| 12 | *GALNT9* | 96 | 132680916 | 132905905 | 257.75 | 1.50E-03 | rs28690866 | 1.30E-05 |
| 17 | *PDK2* | 9 | 48172100 | 48188733 | 59.18 | 1.50E-03 | rs705966 | 2.40E-04 |
| 4 | *CCKAR* | 11 | 26483017 | 26492042 | 51.97 | 1.60E-03 | rs2725301 | 9.60E-05 |
| 12 | *TRPV4* | 23 | 110220891 | 110271212 | 119.45 | 2.00E-03 | rs4565962 | 2.60E-06 |
| 17 | *C17orf77* | 11 | 72581056 | 72590348 | 46.59 | 2.00E-03 | rs524536 | 6.40E-04 |
| 16 | *MARVELD3* | 8 | 71660055 | 71675868 | 38.15 | 2.10E-03 | rs12932850 | 9.30E-04 |
| 20 | *SLX4IP* | 48 | 10415950 | 10604027 | 194.01 | 2.10E-03 | rs66499922 | 4.60E-04 |
| 21 | *C21orf15* | 4 | 15215453 | 15220685 | 25.3 | 2.10E-03 | rs149481432 | 5.40E-04 |
| 3 | *FNDC3B* | 176 | 171757417 | 172118492 | 423.14 | 2.10E-03 | rs16845257 | 4.60E-04 |
| 18 | *SLMO1* | 14 | 12407894 | 12432236 | 73.07 | 2.20E-03 | rs1785658 | 2.60E-04 |
| 8 | *LRRC6* | 23 | 133584200 | 133687863 | 112.66 | 2.30E-03 | rs7834760 | 4.30E-04 |
| 2 | *MARCO* | 29 | 119699744 | 119752236 | 126.89 | 2.40E-03 | rs7559955 | 8.80E-04 |
| 17 | *TSR1* | 6 | 2225981 | 2240678 | 25.18 | 2.60E-03 | rs413016 | 4.00E-04 |
| 16 | *SRL* | 42 | 4239374 | 4292081 | 135.57 | 2.90E-03 | rs879150 | 1.50E-04 |
| 23 | *GABRA3* | 33 | 151335633 | 151619831 | 116.08 | 3.00E-03 | rs1109840 | 4.70E-04 |
| 20 | *C20orf196* | 72 | 5731042 | 5844559 | 190.55 | 3.10E-03 | rs13037060 | 6.40E-04 |
| 11 | *PNPLA2* | 5 | 818900 | 825571 | 28.98 | 3.20E-03 | rs61876744 | 5.60E-04 |
| 23 | *IL1RAPL1* | 151 | 28605680 | 29974017 | 343.22 | 3.30E-03 | rs17282598 | 3.30E-04 |
| 8 | *LOC101929268* | 58 | 49464126 | 49611069 | 168.41 | 3.60E-03 | rs10097910 | 7.70E-04 |
| 7 | *DAGLB* | 27 | 6448746 | 6487643 | 102.22 | 3.60E-03 | rs836540 | 2.60E-04 |
| 17 | *CRHR1* | 11 | 43697709 | 43913194 | 42.57 | 3.70E-03 | rs12950522 | 1.50E-04 |
| 6 | *L3MBTL3* | 56 | 130339727 | 130462594 | 200.81 | 3.80E-03 | rs6899976 | 1.60E-04 |
| 5 | *ITGA1* | 144 | 52084135 | 52249485 | 307.51 | 3.90E-03 | rs12520591 | 1.70E-04 |
| 12 | *GLIPR1* | 7 | 75874512 | 75895716 | 33.56 | 4.00E-03 | rs12422952 | 3.00E-04 |
| 19 | *NLRP13* | 47 | 56407310 | 56443702 | 148.48 | 4.40E-03 | rs2903727 | 7.10E-04 |
| 20 | *ZSWIM3* | 15 | 44486219 | 44507769 | 62.03 | 4.40E-03 | rs113563736 | 6.80E-04 |
| 4 | *CXCL13* | 29 | 78432906 | 78532988 | 98.53 | 4.60E-03 | rs355687 | 7.40E-04 |
| 12 | *UNC119B* | 8 | 121148237 | 121161443 | 31.5 | 4.70E-03 | rs61229807 | 7.10E-04 |
| 21 | *B3GALT5* | 40 | 40984866 | 41034816 | 129.33 | 4.70E-03 | rs634486 | 2.40E-04 |
| 16 | *PHLPP2* | 18 | 71678828 | 71758604 | 83.23 | 4.80E-03 | rs1058747 | 7.40E-04 |
| 4 | *CCNI* | 8 | 77969176 | 77997125 | 32.34 | 5.00E-03 | rs4252786 | 9.10E-04 |
| 18 | *ANKRD30B* | 15 | 14748238 | 14852737 | 58.32 | 5.50E-03 | rs9676211 | 5.80E-04 |
| 16 | *TAT* | 4 | 71600753 | 71610998 | 18.11 | 5.70E-03 | rs2432520 | 7.90E-04 |
| 19 | *FCER2* | 26 | 7753642 | 7767032 | 78 | 5.70E-03 | rs2277991 | 6.10E-04 |
| 8 | *PTP4A3* | 6 | 142431487 | 142442554 | 31.09 | 5.70E-03 | rs9987318 | 3.50E-04 |
| 2 | *LYPD6* | 59 | 150186498 | 150330659 | 198.3 | 5.90E-03 | rs1420356 | 7.70E-04 |
| 2 | *LCLAT1* | 66 | 30670122 | 30867091 | 226.85 | 5.90E-03 | rs71444465 | 5.60E-04 |
| 10 | *C10orf99* | 6 | 85933553 | 85945050 | 31.82 | 6.00E-03 | rs4348833 | 6.70E-04 |
| 2 | *TTC7A* | 140 | 47143267 | 47303275 | 391.9 | 6.30E-03 | rs13432506 | 1.30E-04 |
| 9 | *BRINP1* | 80 | 121928907 | 122131739 | 242.94 | 6.50E-03 | rs10118663 | 6.30E-04 |
| 3 | *LOC101929694* | 7 | 112455295 | 112468166 | 35.04 | 6.80E-03 | rs1387020 | 2.60E-04 |
| 7 | *DNAH11* | 417 | 21582832 | 21941186 | 731.54 | 7.10E-03 | rs17145742 | 4.90E-04 |
| 21 | *COL18A1* | 102 | 46825096 | 46933634 | 257.33 | 8.60E-03 | rs4819115 | 6.20E-04 |
| 2 | *NCK2* | 77 | 106361519 | 106510730 | 238.25 | 9.00E-03 | rs6745887 | 8.50E-04 |
| 16 | *SEC14L5* | 57 | 5008317 | 5069156 | 189.61 | 9.10E-03 | rs12444698 | 3.40E-04 |
| 14 | *EDDM3B* | 11 | 21236585 | 21239107 | 45.56 | 9.40E-03 | rs3827905 | 9.70E-05 |
| 18 | *LOXHD1* | 123 | 44056934 | 44236996 | 279.46 | 9.70E-03 | rs80220231 | 3.70E-04 |
| 6 | *DTNBP1* | 37 | 15523031 | 15663289 | 120.91 | 9.90E-03 | rs9358063 | 9.90E-04 |
| 13 | *LHFP* | 201 | 39917028 | 40177356 | 380.5 | 1.00E-02 | rs9566425 | 4.70E-05 |
| 22 | *MAFF* | 10 | 38597938 | 38612517 | 36.93 | 1.00E-02 | rs73154441 | 9.80E-04 |
| 2 | *FARP2* | 39 | 242295663 | 242434257 | 112.59 | 1.00E-02 | rs1476698 | 9.80E-04 |
| 6 | *E2F3* | 41 | 20402136 | 20493945 | 119.02 | 1.00E-02 | rs4142184 | 8.80E-04 |
| 2 | *CSRNP3* | 92 | 166326156 | 166545917 | 244.78 | 1.00E-02 | rs1007732 | 6.30E-04 |
| 5 | *STK32A* | 106 | 146614578 | 146767418 | 244.58 | 1.00E-02 | rs987651 | 7.20E-04 |
| 23 | *DMD* | 580 | 31137344 | 33357726 | 887.9 | 1.10E-02 | rs1555255 | 3.40E-04 |
| 6 | *AMD1* | 15 | 111135823 | 111216915 | 53.04 | 1.10E-02 | rs1007274 | 1.90E-04 |
| 14 | *RHOJ* | 60 | 63671101 | 63760230 | 172.36 | 1.20E-02 | rs10140212 | 3.30E-04 |
| 19 | *HPN-AS1* | 39 | 35549962 | 35597208 | 98.74 | 1.20E-02 | rs77115335 | 9.70E-04 |
| 21 | *BACH1* | 21 | 30671219 | 30734217 | 71.59 | 1.20E-02 | rs74351375 | 8.70E-04 |
| 3 | *SUCLG2-AS1* | 83 | 67705120 | 67998136 | 204.17 | 1.20E-02 | rs2362446 | 6.40E-04 |
| 12 | *ARNTL2* | 39 | 27485786 | 27578746 | 110.49 | 1.30E-02 | rs12300289 | 6.90E-04 |
| 5 | *FAM196B* | 84 | 169290718 | 169407744 | 186.16 | 1.30E-02 | rs17738017 | 2.20E-04 |
| 12 | *EMP1* | 18 | 13349601 | 13369708 | 51.04 | 1.40E-02 | rs4763327 | 4.20E-04 |
| 8 | *MROH5* | 79 | 142443928 | 142517330 | 205.05 | 1.40E-02 | rs11777061 | 3.90E-04 |
| 5 | *KLHL3* | 33 | 136953188 | 137071779 | 104.45 | 1.50E-02 | rs3813314 | 6.30E-04 |
| 9 | *GPR107* | 33 | 132815984 | 132902448 | 94.53 | 1.50E-02 | rs4837467 | 7.30E-04 |
| 2 | *SP140* | 32 | 231090444 | 231177930 | 97.57 | 1.50E-02 | rs3769847 | 6.30E-04 |
| 23 | *ELF4* | 5 | 129198894 | 129244688 | 19.37 | 1.60E-02 | rs3788848 | 7.70E-04 |
| 7 | *THSD7A* | 386 | 11410061 | 11871824 | 658.62 | 1.60E-02 | rs6962022 | 5.80E-04 |
| 2 | *COL5A2* | 31 | 189896640 | 190044605 | 93.21 | 1.60E-02 | rs4666770 | 5.40E-04 |
| 6 | *COL19A1* | 145 | 70576447 | 70922157 | 314.06 | 1.70E-02 | rs75515009 | 8.90E-05 |
| 7 | *STEAP1B* | 53 | 22459062 | 22539901 | 125.71 | 1.80E-02 | rs73083161 | 7.20E-04 |
| 2 | *LINC00607* | 134 | 216476285 | 216708259 | 287.56 | 1.90E-02 | rs608527 | 3.40E-04 |
| 10 | *ARHGAP22* | 136 | 49654067 | 49864310 | 317.15 | 2.00E-02 | rs7085335 | 7.00E-05 |
| 15 | *TRPM1* | 107 | 31293263 | 31453476 | 280.38 | 2.00E-02 | rs11070796 | 7.90E-04 |
| 10 | *MIR1256_10* | 49 | 74119697 | 74336541 | 152.13 | 2.10E-02 | rs7917581 | 1.10E-04 |
| 10 | *LINC00858* | 7 | 86039735 | 86054415 | 22.52 | 2.10E-02 | rs4933997 | 3.90E-04 |
| 12 | *TMTC1* | 233 | 29653745 | 29937692 | 408.31 | 2.10E-02 | rs34082635 | 2.80E-04 |
| 17 | *SAMD14* | 11 | 48188672 | 48207246 | 33.4 | 2.10E-02 | rs2239953 | 5.30E-04 |
| 9 | *LOC101927502* | 54 | 84304627 | 84391814 | 145.5 | 2.10E-02 | rs4877223 | 3.50E-05 |
| 10 | *TBATA* | 32 | 72530994 | 72545157 | 80.95 | 2.30E-02 | rs34343228 | 3.00E-04 |
| 3 | *LSAMP* | 251 | 115521209 | 116164385 | 487.01 | 2.30E-02 | rs7619638 | 9.70E-05 |
| 15 | *C15orf54* | 6 | 39542884 | 39547048 | 17.19 | 2.40E-02 | rs13329154 | 8.20E-04 |
| 12 | *PTMS* | 4 | 6875540 | 6880118 | 13.93 | 2.50E-02 | rs12313899 | 4.50E-04 |
| 18 | *ZNF521* | 162 | 22641887 | 22932214 | 293.16 | 2.50E-02 | rs59098069 | 3.60E-04 |
| 6 | *LINC01276* | 15 | 41470181 | 41487590 | 36.36 | 2.50E-02 | rs1853833 | 3.20E-04 |
| 4 | *TBC1D1* | 174 | 37892704 | 38140796 | 310.63 | 2.60E-02 | rs3890061 | 8.30E-04 |
| 1 | *RABGAP1L_1* | 159 | 174128551 | 174964445 | 458.57 | 2.60E-02 | rs12565430 | 5.00E-05 |
| 5 | *DOCK2* | 332 | 169064250 | 169510386 | 531.26 | 2.70E-02 | rs17738017 | 2.20E-04 |
| 3 | *FGF12* | 377 | 191857181 | 192445388 | 563.51 | 2.70E-02 | rs76638323 | 2.20E-04 |
| 6 | *KCNQ5* | 265 | 73331570 | 73908573 | 433.63 | 2.80E-02 | rs1328857 | 4.90E-04 |
| 20 | *BCAS1* | 110 | 52560078 | 52687304 | 210.59 | 3.00E-02 | rs158551 | 6.30E-04 |
| 22 | *SYN3* | 421 | 32908539 | 33454377 | 694.43 | 3.10E-02 | rs743742 | 1.30E-04 |
| 9 | *AQP3* | 6 | 33441151 | 33447631 | 15.91 | 3.10E-02 | rs2231235 | 9.60E-04 |
| 19 | *SLC17A7* | 11 | 49932654 | 49944808 | 31.47 | 3.20E-02 | rs11671544 | 4.70E-04 |
| 4 | *STOX2* | 80 | 184826508 | 184938875 | 149.39 | 3.20E-02 | rs12644266 | 6.40E-04 |
| 3 | *ARHGEF3* | 231 | 56761445 | 57113336 | 400.94 | 3.30E-02 | rs1110866 | 1.40E-05 |
| 10 | *CCSER2* | 40 | 86088344 | 86278277 | 93.58 | 3.40E-02 | rs1188793 | 5.20E-04 |
| 14 | *FOXN3* | 313 | 89622515 | 90085494 | 480.47 | 3.40E-02 | rs428839 | 3.80E-04 |
| 9 | *PALM2-AKAP2* | 450 | 112542576 | 112934791 | 752.24 | 3.40E-02 | rs2182815 | 4.80E-04 |
| 19 | *PIAS4* | 11 | 4007748 | 4038067 | 32.66 | 3.50E-02 | rs4806967 | 5.10E-04 |
| 9 | *TLE1* | 95 | 84198597 | 84303596 | 161.07 | 3.50E-02 | rs72747288 | 7.60E-04 |
| 8 | *ZFAT* | 145 | 135490030 | 135725292 | 329.57 | 3.60E-02 | rs10089756 | 3.50E-04 |
| 7 | *DGKI* | 169 | 137074384 | 137531609 | 306.73 | 3.70E-02 | rs4728417 | 9.60E-05 |
| 5 | *RASA1* | 19 | 86564069 | 86687743 | 57.8 | 3.80E-02 | rs3804239 | 4.30E-04 |
| 2 | *LOC101929532* | 20 | 162970950 | 163029243 | 50.8 | 3.80E-02 | rs41368446 | 8.50E-04 |
| 3 | *LOC101928135* | 197 | 34917288 | 35435515 | 358.9 | 3.90E-02 | rs2060661 | 6.20E-04 |
| 2 | *C2orf71* | 28 | 29284555 | 29297127 | 67.83 | 4.00E-02 | rs17744093 | 4.60E-04 |
| 7 | *POU6F2* | 232 | 39017608 | 39504390 | 415.03 | 4.10E-02 | rs7793509 | 9.00E-04 |
| 7 | *ACN9* | 14 | 96745904 | 96811075 | 36.3 | 4.10E-02 | rs7837 | 2.50E-04 |
| 10 | *PTPRE* | 177 | 129705324 | 129884164 | 307.66 | 4.30E-02 | rs7088062 | 4.70E-04 |
| 2 | *KYNU* | 67 | 143635194 | 143799885 | 135.81 | 4.70E-02 | rs7561260 | 6.70E-04 |
