# Supplementary material for: A Genome-Wide Association Study of Age-Related Hearing Impairment in Middle- and Old-Aged Chinese Twins
Source: Biomed Res Int. 2021 Jul 17;2021:3629624. doi: 10.1155/2021/3629624 (PMC8314043; doi:10.1155/2021/3629624)
Supplement: Supplementary 4 — Additional file 4: top 20 genes from VEGAS2 gene-based analysis showing the strongest association with BEHL1.0. [file 3629624.f4.docx]

**Additional file 3**. Top 20 genes from VEGAS2 gene-based analysis showing the strongest association with BEHL_1.0_.

| **Chr** | **Gene** | **nSNPs** | **Start position** | **Stop position** | **Gene-based test statistic** | ***P*-value** | **Top-SNP** | **Top-SNP *P*-value** |
| --- | --- | --- | --- | --- | --- | --- | --- | --- |
| 12 | *OR6C68* | 4 | 55886161 | 55887100 | 36.29 | 1.00E-05 | rs12579181 | 2.60E-04 |
| 12 | *OR6C70* | 6 | 55862983 | 55863922 | 29.97 | 3.00E-05 | rs58920821 | 5.50E-04 |
| 1 | *ADAMTS4* | 9 | 161159537 | 161168845 | 54.23 | 6.30E-05 | rs4233367 | 2.00E-04 |
| 20 | *LOC284801* | 3 | 26167654 | 26189869 | 23.09 | 7.00E-05 | rs12625395 | 3.70E-05 |
| 5 | *LOC101927488* | 10 | 125608209 | 125620867 | 70.66 | 1.30E-04 | rs62391798 | 2.30E-05 |
| 6 | *TRMT11* | 18 | 126307575 | 126360420 | 228.45 | 1.40E-04 | rs9375421 | 3.70E-06 |
| 6 | *HINT3* | 9 | 126277860 | 126301389 | 128.47 | 1.50E-04 | rs9375419 | 9.40E-05 |
| 9 | *RABEPK* | 6 | 127962820 | 127996438 | 41.53 | 2.00E-04 | rs599063 | 4.60E-04 |
| 21 | *COL18A1* | 102 | 46825096 | 46933634 | 391.21 | 2.40E-04 | rs8126757 | 8.70E-05 |
| 12 | *TAS2R9* | 5 | 10961692 | 10962767 | 19.92 | 2.50E-04 | rs3741845 | 2.90E-03 |
| 2 | *PSMD14* | 27 | 162164785 | 162268228 | 214.13 | 2.80E-04 | rs3769968 | 3.40E-04 |
| 4 | *PRMT9* | 5 | 148559533 | 148605280 | 18.86 | 3.10E-04 | rs77293186 | 7.50E-03 |
| 3 | *IQCF6* | 3 | 51812576 | 51813203 | 16.05 | 3.20E-04 | rs60093028 | 4.40E-03 |
| 1 | *AQP10* | 3 | 154293591 | 154297801 | 16.79 | 3.30E-04 | rs6685323 | 3.60E-03 |
| 12 | *TAS2R8* | 2 | 10958649 | 10959579 | 17.61 | 4.00E-04 | rs1548803 | 3.30E-04 |
| 19 | *NLRP13* | 47 | 56407310 | 56443702 | 208.52 | 4.20E-04 | rs12610617 | 3.40E-04 |
| 17 | *PDK2* | 9 | 48172100 | 48188733 | 72.68 | 4.50E-04 | rs705966 | 8.00E-05 |
| 11 | *MRPL16* | 3 | 59573607 | 59578345 | 18.12 | 4.60E-04 | rs2298589 | 4.80E-04 |
| 9 | *SHC3* | 37 | 91620685 | 91793682 | 168.14 | 4.70E-04 | rs944482 | 2.40E-03 |
| 9 | *PPAPDC3* | 10 | 134165080 | 134184649 | 66.66 | 5.50E-04 | rs1541122 | 1.50E-03 |
