# Supplementary material for: A Genome-Wide Association Study of Age-Related Hearing Impairment in Middle- and Old-Aged Chinese Twins
Source: Biomed Res Int. 2021 Jul 17;2021:3629624. doi: 10.1155/2021/3629624 (PMC8314043; doi:10.1155/2021/3629624)
Supplement: Supplementary 5 — Additional file 5: top 20 genes from VEGAS2 gene-based analysis showing the strongest association with BEHL2.0. [file 3629624.f5.docx]

**Additional file 4**. Top 20 genes from VEGAS2 gene-based analysis showing the strongest association with BEHL_2.0_.

| **Chr** | **Gene** | **nSNPs** | **Start position** | **Stop position** | **Gene-based test statistic** | ***P*-value** | **Top-SNP** | **Top-SNP *P*-value** |
| --- | --- | --- | --- | --- | --- | --- | --- | --- |
| 10 | ***SLC16A9*** | 34 | 61410521 | 61469649 | 333.71 | 1.20E-05 | rs3763747 | 1.90E-06 |
| 11 | ***UBQLN3*** | 7 | 5528529 | 5531153 | 44.17 | 1.20E-05 | rs2234456 | 9.80E-07 |
| 15 | *ARID3B* | 12 | 74833547 | 74890472 | 111.9 | 4.40E-05 | rs10851873 | 3.00E-05 |
| 11 | *MYBPC3* | 14 | 47352956 | 47374253 | 82.19 | 5.80E-05 | rs11570058 | 1.40E-07 |
| 15 | *UBL7* | 5 | 74738317 | 74753529 | 76.07 | 1.10E-04 | rs11854461 | 9.50E-05 |
| 21 | *LTN1* | 26 | 30300465 | 30365277 | 146.62 | 1.20E-04 | rs2248903 | 1.20E-03 |
| 15 | *SEMA7A* | 20 | 74701629 | 74726299 | 119.02 | 1.40E-04 | rs11857558 | 2.60E-05 |
| 15 | *CLK3* | 7 | 74900712 | 74922542 | 56.58 | 2.00E-04 | rs2068982 | 7.50E-05 |
| 17 | *LLGL2* | 42 | 73521782 | 73571290 | 230.79 | 2.60E-04 | rs11652563 | 4.00E-04 |
| 15 | *EDC3* | 8 | 74922898 | 74988386 | 62.22 | 2.90E-04 | rs11072498 | 1.10E-04 |
| 18 | *LAMA3* | 67 | 21269561 | 21535029 | 375.48 | 3.20E-04 | rs12608087 | 1.60E-04 |
| 11 | ***OR51I2*** | 6 | 5474637 | 5475707 | 31.88 | 4.00E-04 | rs11037503 | 1.20E-06 |
| 2 | *CEBPZ* | 10 | 37428774 | 37458740 | 64.05 | 4.50E-04 | rs3213746 | 9.80E-04 |
| 21 | *MAP3K7CL* | 45 | 30449791 | 30548210 | 219.65 | 4.70E-04 | rs2832231 | 8.00E-05 |
| 1 | *TTF2* | 25 | 117602948 | 117645491 | 126.14 | 4.80E-04 | rs2274253 | 1.00E-03 |
| 3 | *CLSTN2-AS1* | 2 | 140224459 | 140227631 | 15.77 | 5.00E-04 | rs7615158 | 4.80E-03 |
| 5 | *ZDHHC11* | 5 | 795719 | 851101 | 24.71 | 5.60E-04 | rs67757774 | 3.20E-03 |
| 15 | *TMOD3* | 16 | 52121824 | 52204331 | 125.71 | 6.10E-04 | rs7163541 | 2.20E-04 |
| 1 | *FAM212B* | 28 | 112264685 | 112298419 | 124.86 | 6.10E-04 | rs41314013 | 1.40E-04 |
| 1 | *ADAMTS4* | 9 | 161159537 | 161168845 | 41.94 | 6.10E-04 | rs7512012 | 1.10E-03 |
