# Supplementary material for: A Genome-Wide Association Study of Age-Related Hearing Impairment in Middle- and Old-Aged Chinese Twins
Source: Biomed Res Int. 2021 Jul 17;2021:3629624. doi: 10.1155/2021/3629624 (PMC8314043; doi:10.1155/2021/3629624)
Supplement: Supplementary 6 — Additional file 6: top 20 genes from VEGAS2 gene-based analysis showing the strongest association with BEHL4.0. [file 3629624.f6.docx]

**Additional file 5**. Top 20 genes from VEGAS2 gene-based analysis showing the strongest association with BEHL_4.0_.

| **Chr** | **Gene** | **nSNPs** | **Start position** | **Stop position** | **Gene-based test statistic** | ***P*-value** | **Top-SNP** | **Top-SNP *P*-value** |
| --- | --- | --- | --- | --- | --- | --- | --- | --- |
| 8 | *LOC101929268* | 58 | 49464126 | 49611069 | 297.37 | 1.20E-05 | rs6983178 | 1.80E-05 |
| 23 | *PIR-FIGF* | 18 | 15363712 | 15509432 | 193.95 | 1.60E-05 | rs2071177 | 1.70E-05 |
| 23 | *PIGA* | 5 | 15337572 | 15353676 | 82.19 | 3.70E-05 | rs3661 | 4.50E-05 |
| 12 | *GLTP* | 11 | 110288747 | 110318293 | 100.54 | 5.70E-05 | rs10850913 | 1.00E-06 |
| 2 | *HNMT* | 23 | 138721807 | 138773934 | 225.72 | 8.50E-05 | rs3828168 | 3.00E-05 |
| 19 | *FUT3* | 7 | 5842898 | 5851485 | 38.74 | 1.00E-04 | rs812936 | 2.10E-03 |
| 23 | *PIR* | 17 | 15402923 | 15511711 | 187.85 | 1.10E-04 | rs2071177 | 1.70E-05 |
| 9 | *GTF3C5* | 9 | 135906061 | 135933890 | 72.65 | 1.40E-04 | rs1541331 | 1.70E-04 |
| 4 | *CCNI* | 8 | 77969176 | 77997125 | 52.91 | 1.70E-04 | rs4252786 | 3.70E-04 |
| 10 | *CRTAC1* | 82 | 99624756 | 99790585 | 329.79 | 1.80E-04 | rs4919154 | 1.20E-04 |
| 2 | *LOC100507006* | 11 | 64455534 | 64479665 | 89.99 | 1.90E-04 | rs1025209 | 1.50E-04 |
| 23 | *ASB11* | 4 | 15299830 | 15333746 | 42.55 | 2.20E-04 | rs5935944 | 4.50E-05 |
| 8 | *KLHL38* | 20 | 124657914 | 124665190 | 146.13 | 2.70E-04 | rs11784192 | 2.40E-04 |
| 23 | *IL1RAPL1* | 151 | 28605680 | 29974017 | 425.67 | 2.90E-04 | rs5972128 | 4.50E-05 |
| 16 | *APRT* | 2 | 88875876 | 88878342 | 18.71 | 3.10E-04 | rs8191483 | 6.80E-04 |
| 1 | *CPT2* | 11 | 53662100 | 53679869 | 47.34 | 3.20E-04 | rs11578832 | 3.00E-04 |
| 12 | *CLEC2A* | 11 | 10065825 | 10084980 | 64.79 | 3.30E-04 | rs644565 | 2.50E-04 |
| 17 | *BZRAP1-AS1* | 17 | 56402810 | 56431088 | 103.8 | 3.90E-04 | rs2632527 | 1.60E-04 |
| 5 | *GRIA1* | 140 | 152870083 | 153193429 | 430.81 | 4.10E-04 | rs549100 | 5.50E-04 |
| 6 | *LOC100130357* | 17 | 13279526 | 13295818 | 103.97 | 4.80E-04 | rs1550526 | 2.60E-04 |
