# Supplementary material for: A Genome-Wide Association Study of Age-Related Hearing Impairment in Middle- and Old-Aged Chinese Twins
Source: Biomed Res Int. 2021 Jul 17;2021:3629624. doi: 10.1155/2021/3629624 (PMC8314043; doi:10.1155/2021/3629624)
Supplement: Supplementary 7 — Additional file 7: top 20 genes from VEGAS2 gene-based analysis showing the strongest association with BEHL8.0. [file 3629624.f7.docx]

**Additional file 6**. Top 20 genes from VEGAS2 gene-based analysis showing the strongest association with BEHL_8.0_.

| **Chr** | **Gene** | **nSNPs** | **Start position** | **Stop position** | **Gene-based test statistic** | ***P*-value** | **Top-SNP** | **Top-SNP *P*-value** |
| --- | --- | --- | --- | --- | --- | --- | --- | --- |
| 6 | *FAM184A* | 67 | 119280993 | 119470358 | 454.4 | 5.70E-05 | rs7745460 | 7.80E-06 |
| 5 | *NR3C1* | 78 | 142657495 | 142815077 | 478.96 | 6.00E-05 | rs13306588 | 1.60E-04 |
| 3 | *OTOL1* | 4 | 161214595 | 161221730 | 30.55 | 7.50E-05 | rs3921595 | 6.80E-04 |
| 3 | *LPP-AS1* | 4 | 188280024 | 188286454 | 41.65 | 1.10E-04 | rs13092374 | 1.00E-04 |
| 6 | *SLC22A7* | 5 | 43265997 | 43273276 | 45.34 | 1.50E-04 | rs2270860 | 4.40E-04 |
| 3 | *PSMD6-AS2* | 5 | 63989697 | 63997917 | 48.45 | 1.70E-04 | rs1046025 | 3.00E-04 |
| 4 | *KLHL2* | 16 | 166128769 | 166244308 | 120.74 | 1.80E-04 | rs11726531 | 1.50E-04 |
| 23 | *AMMECR1* | 2 | 109437413 | 109683461 | 28.19 | 1.90E-04 | rs3788769 | 1.70E-04 |
| 22 | *LOC101929664* | 20 | 30404730 | 30476469 | 111.77 | 2.10E-04 | rs41162 | 4.90E-04 |
| 17 | *CD300LD* | 8 | 72576110 | 72588370 | 47.78 | 2.80E-04 | rs1699585 | 6.70E-04 |
| 1 | *SLC35E2B* | 15 | 1592938 | 1624243 | 59.39 | 3.10E-04 | rs4074196 | 2.00E-03 |
| 4 | *LEF1* | 28 | 108968700 | 109090112 | 187.33 | 3.40E-04 | rs17038688 | 4.20E-05 |
| 6 | *NCOA7* | 51 | 126102306 | 126253176 | 265.06 | 3.60E-04 | rs584032 | 4.90E-05 |
| 10 | *PPAPDC1A* | 101 | 122216465 | 122349367 | 316.58 | 4.10E-04 | rs2901245 | 2.80E-04 |
| 14 | *BCL11B* | 96 | 99635624 | 99738050 | 329.31 | 4.10E-04 | rs2793321 | 3.00E-05 |
| 1 | *CDK11B* | 16 | 1570602 | 1655859 | 64.43 | 4.10E-04 | rs4074196 | 2.00E-03 |
| 5 | *G3BP1* | 11 | 151151475 | 151184915 | 68.53 | 4.40E-04 | rs10477000 | 3.80E-04 |
| 2 | *TBC1D8* | 77 | 101623689 | 101767846 | 311.3 | 5.10E-04 | rs2309938 | 1.00E-06 |
| 3 | *EAF2* | 17 | 121554033 | 121605373 | 106.47 | 5.30E-04 | rs10511407 | 5.30E-04 |
| 7 | *TAS2R4* | 6 | 141478288 | 141479188 | 36.19 | 5.50E-04 | rs2233998 | 1.10E-03 |
