# Supplementary material for: A Genome-Wide Association Study of Age-Related Hearing Impairment in Middle- and Old-Aged Chinese Twins
Source: Biomed Res Int. 2021 Jul 17;2021:3629624. doi: 10.1155/2021/3629624 (PMC8314043; doi:10.1155/2021/3629624)
Supplement: Supplementary 8 — Additional file 8: top 20 genes from VEGAS2 gene-based analysis showing the strongest association with PTA. [file 3629624.f8.docx]

**Additional file 7**. Top 20 genes from VEGAS2 gene-based analysis showing the strongest association with PTA.

| **Chr** | **Gene** | **nSNPs** | **Start position** | **Stop position** | **Gene-based test statistic** | ***P*-value** | **Top-SNP** | **Top-SNP *P*-value** |
| --- | --- | --- | --- | --- | --- | --- | --- | --- |
| 11 | *RRP8* | 9 | 6621143 | 6624880 | 55.09 | 9.00E-06 | rs17834692 | 4.20E-04 |
| 12 | *GLTP* | 11 | 110288747 | 110318293 | 106.95 | 2.30E-05 | rs10850913 | 2.30E-06 |
| 1 | *ADAMTS4* | 9 | 161159537 | 161168845 | 56.79 | 5.20E-05 | rs4233367 | 1.80E-04 |
| 12 | *GLIPR1L2* | 12 | 75784849 | 75826177 | 102.32 | 5.30E-05 | rs7300308 | 1.20E-05 |
| 23 | *PIR-FIGF* | 18 | 15363712 | 15509432 | 171.28 | 7.80E-05 | rs170905 | 4.90E-05 |
| 23 | *PIR* | 17 | 15402923 | 15511711 | 186.64 | 1.10E-04 | rs170905 | 4.90E-05 |
| 3 | *C3orf70* | 40 | 184795837 | 184870802 | 190.09 | 1.50E-04 | rs2132240 | 1.50E-05 |
| 4 | *KLHL2* | 16 | 166128769 | 166244308 | 123.2 | 1.50E-04 | rs117045966 | 3.80E-05 |
| 3 | *VPS8* | 49 | 184529930 | 184770402 | 308.62 | 1.70E-04 | rs2271257 | 1.60E-04 |
| 23 | *PIGA* | 5 | 15337572 | 15353676 | 66.6 | 2.20E-04 | rs3661 | 2.30E-04 |
| 22 | *MCAT* | 8 | 43528211 | 43539403 | 67.42 | 2.40E-04 | rs2072852 | 7.40E-04 |
| 3 | *OTOL1* | 4 | 161214595 | 161221730 | 26.02 | 2.60E-04 | rs3921595 | 2.50E-03 |
| 16 | *ZNF23* | 4 | 71481502 | 71496117 | 29.49 | 2.90E-04 | rs1609869 | 1.00E-03 |
| 19 | *PSG11* | 3 | 43511808 | 43530631 | 17.07 | 3.30E-04 | rs111493043 | 2.70E-03 |
| 7 | *PDE1C* | 357 | 31790792 | 32339016 | 862.51 | 4.40E-04 | rs6968484 | 7.90E-05 |
| 6 | *PSORS1C2* | 22 | 31105310 | 31107127 | 71.11 | 4.60E-04 | rs1265094 | 2.90E-03 |
| 20 | *POFUT1* | 4 | 30795695 | 30826467 | 18.55 | 4.80E-04 | rs79984831 | 4.70E-03 |
| 7 | *FAM131B* | 5 | 143050492 | 143059840 | 28.08 | 5.10E-04 | rs4236482 | 2.40E-03 |
| 1 | *RC3H1* | 9 | 173900351 | 173962210 | 69.02 | 5.20E-04 | rs9425780 | 2.10E-04 |
| 1 | *RHBG* | 21 | 156338979 | 156355013 | 97.68 | 6.30E-04 | rs11585523 | 3.00E-04 |
