# Supplementary material for: A Genome-Wide Association Study of Age-Related Hearing Impairment in Middle- and Old-Aged Chinese Twins
Source: Biomed Res Int. 2021 Jul 17;2021:3629624. doi: 10.1155/2021/3629624 (PMC8314043; doi:10.1155/2021/3629624)
Supplement: Supplementary 9 — Additional file 9: top 20 KEGG, Reactome, and Biocarta pathway results for BEHL1.0 in the typed GWAS data. [file 3629624.f9.docx]

**Additional file 8.** Top 20 KEGG, Reactome, and Biocarta (emp-*P* < 0.05) pathway results for BEHL_1.0_ in the typed GWAS data.

| Pathway | chisq-*P* | emp-*P* | log(chisq*P*) | log(emp*P*) |
| --- | --- | --- | --- | --- |
| KEGG_WNT_SIGNALING_PATHWAY | 3.52E-04 | 1.47E-04 | 3.45324 | 3.83268 |
| REACTOME_TCA_CYCLE_AND_RESPIRATORY_ELECTRON_TRANSPORT | 4.75E-04 | 1.79E-04 | 3.32286 | 3.74715 |
| KEGG_PROTEASOME | 6.53E-04 | 6.10E-04 | 3.18489 | 3.21467 |
| REACTOME_SYNTHESIS_OF_PIPS_AT_THE_EARLY_ENDOSOME_MEMBRANE | 1.96E-03 | 6.20E-04 | 2.70732 | 3.20761 |
| REACTOME_SYNTHESIS_OF_PIPS_AT_THE_PLASMA_MEMBRANE | 1.96E-03 | 6.70E-04 | 2.70732 | 3.17393 |
| BIOCARTA_PROTEASOME_PATHWAY | 6.95E-04 | 6.90E-04 | 3.15776 | 3.16115 |
| REACTOME_PI_METABOLISM | 1.96E-03 | 7.10E-04 | 2.70732 | 3.14874 |
| BIOCARTA_STATHMIN_PATHWAY | 2.73E-03 | 9.00E-04 | 2.56316 | 3.04576 |
| REACTOME_AMINE_COMPOUND_SLC_TRANSPORTERS | 1.02E-03 | 1.00E-03 | 2.99078 | 3.00000 |
| REACTOME_NA_CL_DEPENDENT_NEUROTRANSMITTER_TRANSPORTERS | 1.02E-03 | 1.09E-03 | 2.99078 | 2.96257 |
| REACTOME_PYRUVATE_METABOLISM | 2.55E-03 | 1.13E-03 | 2.59414 | 2.94692 |
| REACTOME_PYRUVATE_METABOLISM_AND_CITRIC_ACID_TCA_CYCLE | 2.55E-03 | 1.15E-03 | 2.59414 | 2.93930 |
| KEGG_O_GLYCAN_BIOSYNTHESIS | 1.31E-03 | 1.16E-03 | 2.88366 | 2.93554 |
| KEGG_SMALL_CELL_LUNG_CANCER | 3.58E-03 | 1.22E-03 | 2.44580 | 2.91364 |
| REACTOME_O_LINKED_GLYCOSYLATION_OF_MUCINS | 1.31E-03 | 1.26E-03 | 2.88366 | 2.89963 |
| REACTOME_INTERFERON_ALPHA_BETA_SIGNALING | 1.34E-03 | 1.39E-03 | 2.87157 | 2.85699 |
| REACTOME_REGULATION_OF_ORNITHINE_DECARBOXYLASE_ODC | 1.09E-03 | 1.45E-03 | 2.96078 | 2.83863 |
| REACTOME_CTNNB1_PHOSPHORYLATION_CASCADE | 4.53E-03 | 1.75E-03 | 2.34391 | 2.75696 |
| BIOCARTA_WNT_PATHWAY | 4.53E-03 | 1.76E-03 | 2.34391 | 2.75449 |
| REACTOME_SIGNALING_BYWNT | 4.70E-03 | 1.83E-03 | 2.32810 | 2.73755 |
