# Supplementary material for: A Genome-Wide Association Study of Age-Related Hearing Impairment in Middle- and Old-Aged Chinese Twins
Source: Biomed Res Int. 2021 Jul 17;2021:3629624. doi: 10.1155/2021/3629624 (PMC8314043; doi:10.1155/2021/3629624)
Supplement: Supplementary 10 — Additional file 10: top 20 KEGG, Reactome, and Biocarta pathway results for BEHL2.0 in the typed GWAS data. [file 3629624.f10.docx]

**Additional file 9.** Top 20 KEGG, Reactome, and Biocarta (emp-*P* < 0.05) pathway results for BEHL_2.0_ in the typed GWAS data.

| Pathway | chisq-*P* | | emp-*P* | log(chisq*P*) | log(emp*P*) |
| --- | --- | --- | --- | --- | --- |
| KEGG_PHOSPHATIDYLINOSITOL_SIGNALING_SYSTEM | | 5.33E-04 | 9.20E-05 | 3.27347 | 4.03621 |
| KEGG_O_GLYCAN_BIOSYNTHESIS | | 7.16E-04 | 1.20E-04 | 3.14494 | 3.92082 |
| REACTOME_METABOLISM_OF_PROTEINS | | 9.12E-04 | 3.25E-04 | 3.04019 | 3.48812 |
| REACTOME_POST_TRANSLATIONAL_PROTEIN_MODIFICATION | | 1.62E-03 | 5.30E-04 | 2.79142 | 3.27572 |
| BIOCARTA_EIF_PATHWAY 0.5 | | 1.61E-03 | 7.10E-04 | 2.79264 | 3.14874 |
| REACTOME_SYNTHESIS_OF_PIPS_AT_THE_EARLY_ENDOSOME_MEMBRANE | | 2.83E-03 | 8.80E-04 | 2.54842 | 3.05552 |
| REACTOME_SYNTHESIS_OF_PIPS_AT_THE_PLASMA_MEMBRANE | | 2.83E-03 | 8.80E-04 | 2.54842 | 3.05552 |
| BIOCARTA_HIVNEF_PATHWAY PTA | | 1.10E-03 | 9.30E-04 | 2.95761 | 3.03152 |
| REACTOME_SLC_MEDIATEDTRANSMEMBRANE_TRANSPORT | | 1.39E-03 | 9.30E-04 | 2.85804 | 3.03152 |
| REACTOME_PI_METABOLISM | | 2.83E-03 | 9.50E-04 | 2.54842 | 3.02228 |
| BIOCARTA_PTEN_PATHWAY | | 3.51E-03 | 9.60E-04 | 2.45482 | 3.01773 |
| REACTOME_TRANSPORT_OFGLUCOSE_AND_OTHER_SUGARS_BILE_SALTS_AND_ORGANIC_ACIDS_METAL_IONS_AND_AMINE_COMPOUNDS | | 9.52E-04 | 9.60E-04 | 3.02119 | 3.01773 |
| REACTOME_BILE_SALT_AND_ORGANIC_ANION_SLC_TRANSPORTERS | | 9.52E-04 | 9.70E-04 | 3.02119 | 3.01323 |
| REACTOME_ACTIVATION_OF_THE_MRNA_UPON_BINDING_OF_THE_CAP_BINDING_COMPLEX_AND_EIFS_AND_SUBSEQUENT_BINDING_TO_43S | | 1.86E-03 | 1.02E-03 | 2.73125 | 2.99140 |
| REACTOME_INTERFERON_SIGNALING | | 1.58E-03 | 1.08E-03 | 2.80139 | 2.96658 |
| REACTOME_DEADENYLATION_OF_MRNA | | 2.00E-03 | 1.12E-03 | 2.69811 | 2.95078 |
| KEGG_INOSITOL_PHOSPHATE_METABOLISM | | 1.27E-03 | 1.24E-03 | 2.89708 | 2.90658 |
| REACTOME_METABOLISM_OF_PROTEINS | | 1.27E-03 | 1.32E-03 | 2.89510 | 2.87943 |
| REACTOME_COSTIMULATION_BY_THE_CD28_FAMILY | | 4.84E-03 | 1.51E-03 | 2.31475 | 2.82102 |
| BIOCARTA_PYK2_PATHWAY | | 2.59E-03 | 1.71E-03 | 2.58753 | 2.76700 |
