# Supplementary material for: A Genome-Wide Association Study of Age-Related Hearing Impairment in Middle- and Old-Aged Chinese Twins
Source: Biomed Res Int. 2021 Jul 17;2021:3629624. doi: 10.1155/2021/3629624 (PMC8314043; doi:10.1155/2021/3629624)
Supplement: Supplementary 11 — Additional file 11: top 20 KEGG, Reactome, and Biocarta pathway results for BEHL4.0 in the typed GWAS data. [file 3629624.f11.docx]

**Additional file 10.** Top 20 KEGG, Reactome, and Biocarta (emp-*P* < 0.05) pathway results for BEHL_4.0_ in the typed GWAS data.

| Pathway | chisq-*P* | emp-*P* | log(chisq*P*) | log(emp*P*) |
| --- | --- | --- | --- | --- |
| REACTOME_SULFUR_AMINOACID_METABOLISM | 5.17E-04 | 1.62E-04 | 3.28625 | 3.79048 |
| KEGG_CYSTEINE_AND_METHIONINE_METABOLISM | 5.17E-04 | 1.71E-04 | 3.28625 | 3.76700 |
| REACTOME_METABOLISM_OF_AMINO_ACIDS_AND_DERIVATIVES | 4.42E-04 | 2.21E-04 | 3.35491 | 3.65561 |
| REACTOME_METABOLISM_OF_AMINO_ACIDS_AND_DERIVATIVES | 2.47E-03 | 3.34E-04 | 2.60808 | 3.47625 |
| KEGG_HISTIDINE_METABOLISM | 6.95E-04 | 5.50E-04 | 3.15776 | 3.25964 |
| KEGG_PATHOGENIC_ESCHERICHIA_COLI_INFECTION | 3.46E-03 | 1.04E-03 | 2.46127 | 2.98297 |
| KEGG_REGULATION_OF_AUTOPHAGY | 2.52E-03 | 1.10E-03 | 2.59943 | 2.95861 |
| KEGG_ADHERENS_JUNCTION | 2.69E-03 | 1.40E-03 | 2.57068 | 2.85387 |
| KEGG_PHENYLALANINE_METABOLISM | 1.34E-03 | 1.54E-03 | 2.87157 | 2.81248 |
| REACTOME_SMOOTH_MUSCLE_CONTRACTION | 7.23E-03 | 1.80E-03 | 2.14114 | 2.74473 |
| KEGG_TAURINE_AND_HYPOTAURINE_METABOLISM | 2.04E-03 | 1.82E-03 | 2.68975 | 2.73993 |
| REACTOME_PACKAGING_OFTELOMERE_ENDS | 2.28E-03 | 1.89E-03 | 2.64260 | 2.72354 |
| BIOCARTA_AMI_PATHWAY | 2.19E-03 | 1.99E-03 | 2.65954 | 2.70115 |
| KEGG_RIBOSOME | 5.31E-03 | 2.27E-03 | 2.27530 | 2.64397 |
| BIOCARTA_PML_PATHWAY | 2.44E-03 | 2.30E-03 | 2.61309 | 2.63827 |
| KEGG_HISTIDINE_METABOLISM | 2.68E-03 | 2.73E-03 | 2.57195 | 2.56384 |
| KEGG_TRYPTOPHAN_METABOLISM | 3.34E-03 | 2.93E-03 | 2.47667 | 2.53313 |
| KEGG_TYROSINE_METABOLISM PTA | 2.73E-03 | 2.97E-03 | 2.56337 | 2.52724 |
| REACTOME_TELOMERE_MAINTENANCE | 3.30E-03 | 3.31E-03 | 2.48208 | 2.48017 |
| REACTOME_G_ALPHA_S_SIGNALLING_EVENTS | 4.46E-03 | 3.36E-03 | 2.35041 | 2.47366 |
