# Supplementary material for: A Genome-Wide Association Study of Age-Related Hearing Impairment in Middle- and Old-Aged Chinese Twins
Source: Biomed Res Int. 2021 Jul 17;2021:3629624. doi: 10.1155/2021/3629624 (PMC8314043; doi:10.1155/2021/3629624)
Supplement: Supplementary 12 — Additional file 12: top 20 KEGG, Reactome, and Biocarta pathway results for BEHL8.0 in the typed GWAS data. [file 3629624.f12.docx]

**Additional file 11.** Top 20 KEGG, Reactome, and Biocarta (emp-*P* < 0.05) pathway results for BEHL_8.0_ in the typed GWAS data.

| Pathway | chisq-*P* | emp-*P* | log(chisq*P*) | log(emp*P*) |
| --- | --- | --- | --- | --- |
| REACTOME_NUCLEOTIDE_EXCISION_REPAIR | 1.50E-03 | 5.80E-04 | 2.82333 | 3.23657 |
| REACTOME_TRANSCRIPTION_COUPLED_NER_TC_NER | 1.50E-03 | 5.90E-04 | 2.82333 | 3.22915 |
| REACTOME_FORMATION_OFTRANSCRIPTION_COUPLED_NER_TC_NER_REPAIR_COMPLEX | 1.50E-03 | 6.00E-04 | 2.82333 | 3.22185 |
| KEGG_NUCLEOTIDE_EXCISION_REPAIR | 1.50E-03 | 6.50E-04 | 2.82333 | 3.18709 |
| KEGG_ECM_RECEPTOR_INTERACTION | 2.34E-03 | 7.80E-04 | 2.63069 | 3.10791 |
| REACTOME_MRNA_3_END_PROCESSING | 1.08E-03 | 1.16E-03 | 2.96531 | 2.93554 |
| KEGG_MELANOGENESIS | 2.50E-03 | 1.21E-03 | 2.60236 | 2.91721 |
| KEGG_LONG_TERM_DEPRESSION | 4.32E-03 | 1.55E-03 | 2.36468 | 2.80967 |
| KEGG_BASAL_CELL_CARCINOMA | 3.93E-03 | 1.56E-03 | 2.40608 | 2.80688 |
| BIOCARTA_TCR_PATHWAY | 4.02E-03 | 1.60E-03 | 2.39529 | 2.79588 |
| BIOCARTA_PDGF_PATHWAY | 4.02E-03 | 1.65E-03 | 2.39529 | 2.78252 |
| KEGG_LEISHMANIA_INFECTION | 1.90E-03 | 1.74E-03 | 2.72016 | 2.75945 |
| REACTOME_SIGNALING_BYFGFR_IN_DISEASE | 1.98E-03 | 1.76E-03 | 2.70434 | 2.75449 |
| BIOCARTA_EGF_PATHWAY | 4.02E-03 | 1.84E-03 | 2.39529 | 2.73518 |
| REACTOME_A_TETRASACCHARIDE_LINKER_SEQUENCE_IS_REQUIRED_FOR_GAG_SYNTHESIS | 2.09E-03 | 1.88E-03 | 2.68034 | 2.72584 |
| KEGG_BLADDER_CANCER | 4.18E-03 | 1.95E-03 | 2.37853 | 2.70997 |
| REACTOME_MHC_CLASS_II_ANTIGEN_PRESENTATION | 2.77E-03 | 2.04E-03 | 2.55678 | 2.69037 |
| REACTOME_APC_C_CDC20_MEDIATED_DEGRADATION_OF_MITOTIC_PROTEINS | 5.41E-03 | 2.11E-03 | 2.26640 | 2.67572 |
| BIOCARTA_P38MAPK_PATHWAY | 5.64E-03 | 2.22E-03 | 2.24852 | 2.65365 |
| KEGG_ADHERENS_JUNCTION | 2.53E-03 | 2.32E-03 | 2.59605 | 2.63451 |
