# Supplementary material for: A Genome-Wide Association Study of Age-Related Hearing Impairment in Middle- and Old-Aged Chinese Twins
Source: Biomed Res Int. 2021 Jul 17;2021:3629624. doi: 10.1155/2021/3629624 (PMC8314043; doi:10.1155/2021/3629624)
Supplement: Supplementary 13 — Additional file 13: top 20 KEGG, Reactome, and Biocarta pathway results for PTA in the typed GWAS data. [file 3629624.f13.docx]

**Additional file 12.** Top 20 KEGG, Reactome, and Biocarta (emp-*P* < 0.05) pathway results for PTA in the typed GWAS data.

| Pathway | chisq-*P* | emp-*P* | log(chisq*P*) | log(emp*P*) |
| --- | --- | --- | --- | --- |
| REACTOME_SIGNALING_BYRHO_GTPASES | 1.68E-03 | 4.01E-04 | 2.77407 | 3.39686 |
| REACTOME_APOPTOSIS | 1.50E-03 | 6.50E-04 | 2.82489 | 3.18709 |
| REACTOME_RNA_POL_I_RNA_POL_III_AND_MITOCHONDRIAL_TRANSCRIPTION | 1.53E-03 | 9.30E-04 | 2.81593 | 3.03152 |
| BIOCARTA_HIVNEF_PATHWAY | 6.89E-04 | 1.06E-03 | 3.16174 | 2.97469 |
| REACTOME_NCAM1_INTERACTIONS | 1.12E-03 | 1.20E-03 | 2.95085 | 2.92082 |
| BIOCARTA_AMI_PATHWAY | 1.31E-03 | 1.23E-03 | 2.88138 | 2.91009 |
| KEGG_ECM_RECEPTOR_INTERACTION | 6.88E-03 | 1.56E-03 | 2.16228 | 2.80688 |
| REACTOME_AXON_GUIDANCE | 4.26E-03 | 1.64E-03 | 2.37029 | 2.78516 |
| KEGG_TYROSINE_METABOLISM | 3.40E-03 | 1.98E-03 | 2.46815 | 2.70333 |
| REACTOME_RNA_POL_I_TRANSCRIPTION | 3.74E-03 | 2.02E-03 | 2.42740 | 2.69465 |
| REACTOME_DEVELOPMENTAL_BIOLOGY | 2.70E-03 | 2.09E-03 | 2.56817 | 2.67985 |
| REACTOME_PRE_NOTCH_EXPRESSION_AND_PROCESSING | 2.09E-03 | 2.14E-03 | 2.68034 | 2.66959 |
| REACTOME_PRE_NOTCH_TRANSCRIPTION_AND_TRANSLATION | 2.09E-03 | 2.17E-03 | 2.68034 | 2.66354 |
| REACTOME_MITOTIC_G2_G2M_PHASES | 2.09E-03 | 2.26E-03 | 2.68034 | 2.64589 |
| REACTOME_DEVELOPMENTAL_BIOLOGY | 3.22E-03 | 2.37E-03 | 2.49214 | 2.62525 |
| REACTOME_NCAM_SIGNALING_FOR_NEURITE_OUT_GROWTH | 4.90E-03 | 2.45E-03 | 2.31001 | 2.61083 |
| REACTOME_EXTRINSIC_PATHWAY_FOR_APOPTOSIS | 2.55E-03 | 2.48E-03 | 2.59329 | 2.60555 |
| BIOCARTA_ARF_PATHWAY | 1.55E-03 | 2.61E-03 | 2.81023 | 2.58336 |
| BIOCARTA_G1_PATHWAY | 1.11E-03 | 2.62E-03 | 2.95475 | 2.58170 |
| REACTOME_APOPTOTIC_CLEAVAGE_OF_CELLULAR_PROTEINS | 2.20E-03 | 2.66E-03 | 2.65702 | 2.57512 |
